# Supplementary material for: Unexpected predicted length variation for the coding sequence of the sleep related gene, BHLHE41 in gorilla amidst strong purifying selection across mammals
Source: PLoS One. 2020 Apr 14;15(4):e0223203. doi: 10.1371/journal.pone.0223203 (PMC7156063; doi:10.1371/journal.pone.0223203)
Supplement: S1 Fig — (DOCX) [file pone.0223203.s001.docx]

**
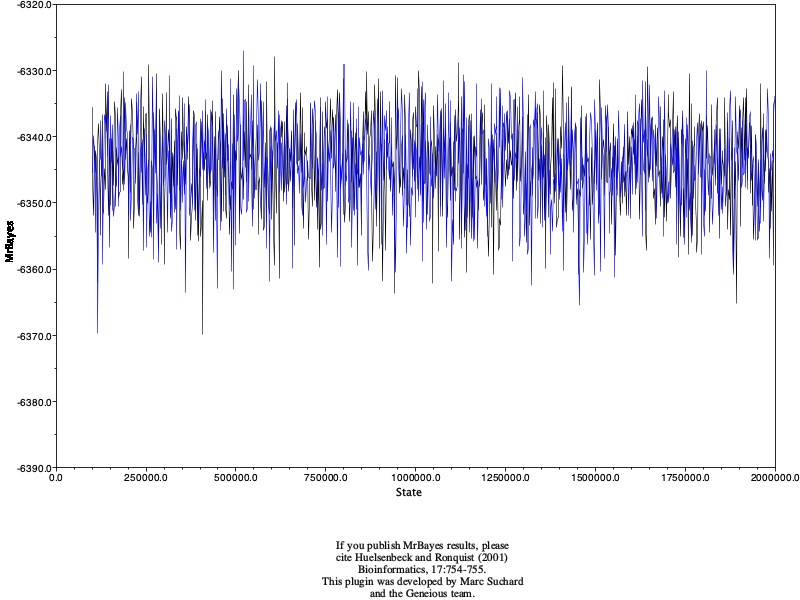
**

**S1 Fig. Verification that the Bayesian MCMC phylogenetic search reached stationarity.** Since there is no trending slope in log likelihood, the two runs have converged. We used one million generations as the burnin just to be safe.
