# Supplementary material for: Unexpected predicted length variation for the coding sequence of the sleep related gene, BHLHE41 in gorilla amidst strong purifying selection across mammals
Source: PLoS One. 2020 Apr 14;15(4):e0223203. doi: 10.1371/journal.pone.0223203 (PMC7156063; doi:10.1371/journal.pone.0223203)
Supplement: S2 Fig — (DOCX) [file pone.0223203.s002.docx]

**
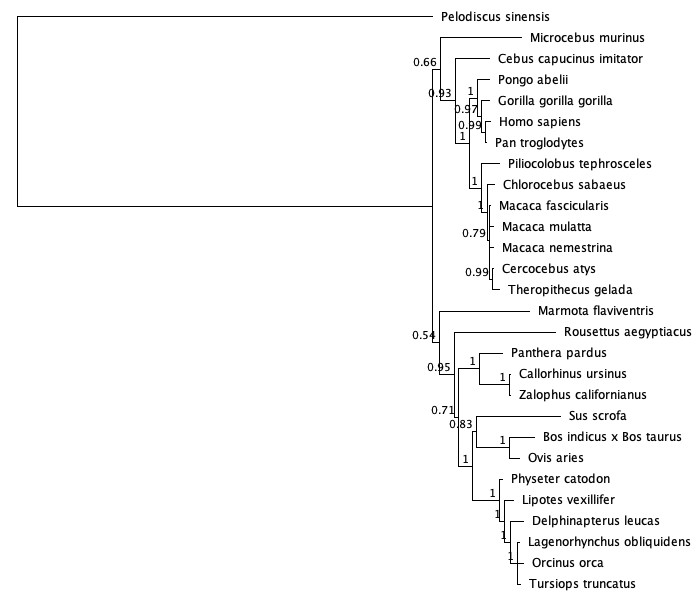
**

**S2 Fig. Bayesian phylogenetic analysis of mammalian *BHLHE41* coding sequence.** The GTR model was used with the “invgamma” Rate Variation. 2,000,000 generations were used, subsampling 1000 trees after 1,000,000. A heated chain temperature of 0.2 was used for two runs with four chains each. The values along the branches indicate the posterior probabilities of 1000 trees. The tree is rooted with the reptilian outgroup, *Pelodiscus sinensis*.
