# Supplementary material for: Unexpected predicted length variation for the coding sequence of the sleep related gene, BHLHE41 in gorilla amidst strong purifying selection across mammals
Source: PLoS One. 2020 Apr 14;15(4):e0223203. doi: 10.1371/journal.pone.0223203 (PMC7156063; doi:10.1371/journal.pone.0223203)
Supplement: S3 Fig — (DOCX) [file pone.0223203.s003.docx]

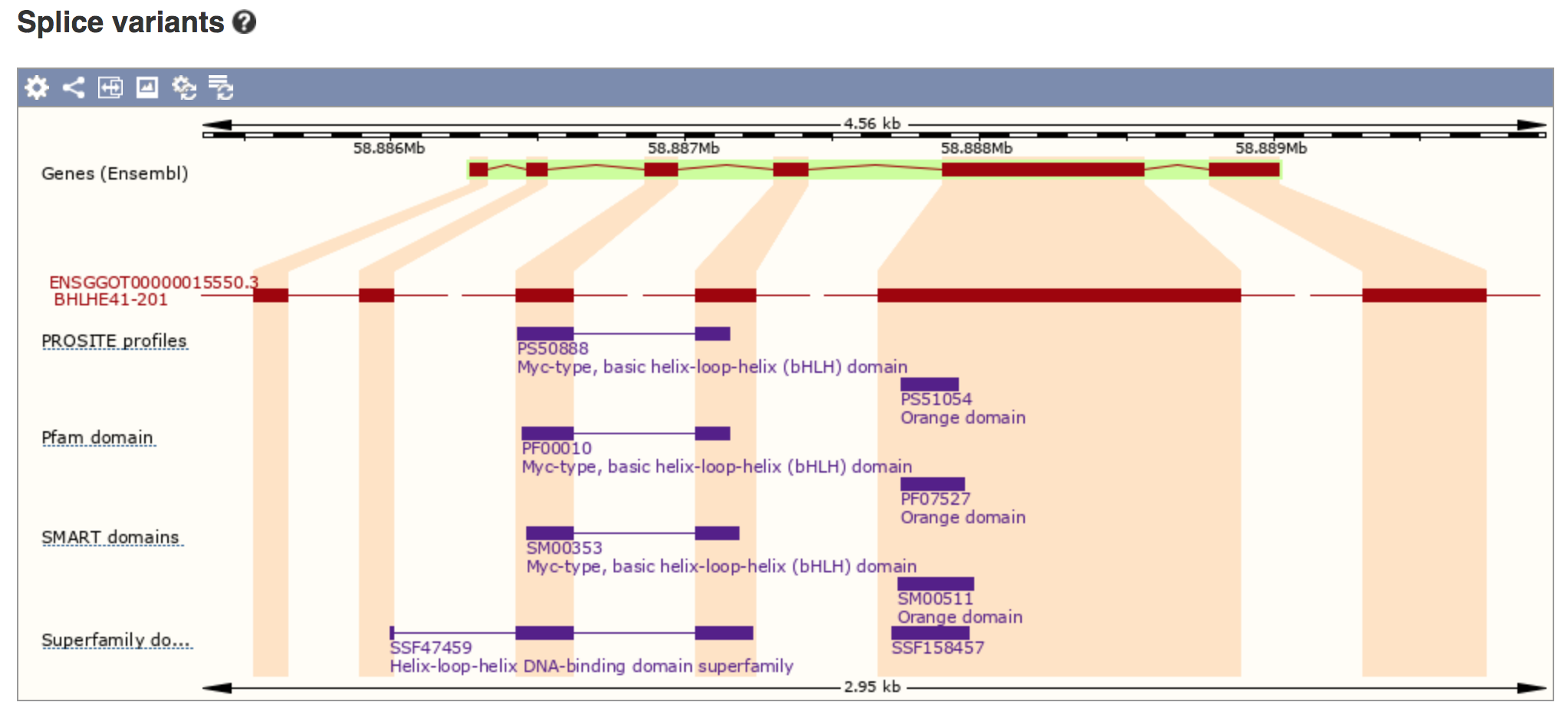


**S3 Fig. EMBL structure of the transcript for *BHLHE41* from *Gorilla gorilla gorilla* with conserved domains indicated.** There are six exons and five introns. EMBL single transcript length is 1260bp. This annotation is different from Genbank XM_019037881 which contains a 318bp insertion on the 5’ end and a 195bp deletion in the fifth exon.
