# Supplementary material for: Unexpected predicted length variation for the coding sequence of the sleep related gene, BHLHE41 in gorilla amidst strong purifying selection across mammals
Source: PLoS One. 2020 Apr 14;15(4):e0223203. doi: 10.1371/journal.pone.0223203 (PMC7156063; doi:10.1371/journal.pone.0223203)
Supplement: S1 Table — (DOCX) [file pone.0223203.s004.docx]

S1 Table. Pairwise codon-based test of purifying selection for mammalian BHLHE41. The p-value is the probability of rejecting the null hypothesis of strict-neutrality (dN = dS) in favor of the alternative hypothesis (dN < dS). dN and dS are the nonsynonymous and synonymous substitution rates, respectively. The variance of the difference was computed using the analytical method. Analyses were conducted using the Nei-Gojobori method (1986) in Mega7 (Kumar et al. 2016). This analysis involved 28 nucleotide sequences and 597 codons.

| Species 1 | Species 2 | p-value | dN-dS |
| --- | --- | --- | --- |
| NM 030762 Homo sapiens | XM 520805: Pan troglodytes | 0.042 | -1.739 |
| NM 030762 Homo sapiens | XM 019037881: Gorilla gorilla gorilla | 0.002 | -2.865 |
| XM 520805: Pan troglodytes | XM 019037881: Gorilla gorilla gorilla | 0.013 | -2.254 |
| NM 030762 Homo sapiens | XM 002823045: Pongo abelii | 0.000 | -3.553 |
| XM 520805: Pan troglodytes | XM 002823045: Pongo abelii | 0.001 | -3.073 |
| XM 019037881: Gorilla gorilla gorilla | XM 002823045: Pongo abelii | 0.001 | -3.253 |
| NM 030762 Homo sapiens | XM 005570417: Macaca fascicularis | 0.000 | -4.622 |
| XM 520805: Pan troglodytes | XM 005570417: Macaca fascicularis | 0.000 | -4.247 |
| XM 019037881: Gorilla gorilla gorilla | XM 005570417: Macaca fascicularis | 0.000 | -3.862 |
| XM 002823045: Pongo abelii | XM 005570417: Macaca fascicularis | 0.000 | -4.406 |
| NM 030762 Homo sapiens | XM 012093655: Cercocebus atys | 0.000 | -4.742 |
| XM 520805: Pan troglodytes | XM 012093655: Cercocebus atys | 0.000 | -4.375 |
| XM 019037881: Gorilla gorilla gorilla | XM 012093655: Cercocebus atys | 0.000 | -4.002 |
| XM 002823045: Pongo abelii | XM 012093655: Cercocebus atys | 0.000 | -4.530 |
| XM 005570417: Macaca fascicularis | XM 012093655: Cercocebus atys | 0.159 | -1.001 |
| NM 030762 Homo sapiens | XM 015151321: Macaca mulatta | 0.000 | -4.651 |
| XM 520805: Pan troglodytes | XM 015151321: Macaca mulatta | 0.000 | -4.278 |
| XM 019037881: Gorilla gorilla gorilla | XM 015151321: Macaca mulatta | 0.000 | -3.899 |
| XM 002823045: Pongo abelii | XM 015151321: Macaca mulatta | 0.000 | -4.438 |
| XM 005570417: Macaca fascicularis | XM 015151321: Macaca mulatta | 0.271 | -0.611 |
| XM 012093655: Cercocebus atys | XM 015151321: Macaca mulatta | 0.130 | -1.133 |
| NM 030762 Homo sapiens | XM 011759130: Macaca nemestrina | 0.000 | -4.860 |
| XM 520805: Pan troglodytes | XM 011759130: Macaca nemestrina | 0.000 | -4.500 |
| XM 019037881: Gorilla gorilla gorilla | XM 011759130: Macaca nemestrina | 0.000 | -4.138 |
| XM 002823045: Pongo abelii | XM 011759130: Macaca nemestrina | 0.000 | -4.652 |
| XM 005570417: Macaca fascicularis | XM 011759130: Macaca nemestrina | 0.079 | -1.418 |
| XM 012093655: Cercocebus atys | XM 011759130: Macaca nemestrina | 0.042 | -1.739 |
| XM 015151321: Macaca mulatta | XM 011759130: Macaca nemestrina | 0.068 | -1.503 |
| NM 030762 Homo sapiens | XM 007967990: Chlorocebus sabaeus | 0.000 | -4.561 |
| XM 520805: Pan troglodytes | XM 007967990: Chlorocebus sabaeus | 0.000 | -4.181 |
| XM 019037881: Gorilla gorilla gorilla | XM 007967990: Chlorocebus sabaeus | 0.000 | -3.756 |
| XM 002823045: Pongo abelii | XM 007967990: Chlorocebus sabaeus | 0.000 | -4.346 |
| XM 005570417: Macaca fascicularis | XM 007967990: Chlorocebus sabaeus | 0.102 | -1.277 |
| XM 012093655: Cercocebus atys | XM 007967990: Chlorocebus sabaeus | 0.055 | -1.606 |
| XM 015151321: Macaca mulatta | XM 007967990: Chlorocebus sabaeus | 0.080 | -1.413 |
| XM 011759130: Macaca nemestrina | XM 007967990: Chlorocebus sabaeus | 0.031 | -1.886 |
| NM 030762 Homo sapiens | XM 023209042: Piliocolobus tephrosceles | 0.000 | -4.510 |
| XM 520805: Pan troglodytes | XM 023209042: Piliocolobus tephrosceles | 0.000 | -4.127 |
| XM 019037881: Gorilla gorilla gorilla | XM 023209042: Piliocolobus tephrosceles | 0.000 | -3.698 |
| XM 002823045: Pongo abelii | XM 023209042: Piliocolobus tephrosceles | 0.000 | -4.547 |
| XM 005570417: Macaca fascicularis | XM 023209042: Piliocolobus tephrosceles | 0.002 | -2.870 |
| XM 012093655: Cercocebus atys | XM 023209042: Piliocolobus tephrosceles | 0.001 | -3.044 |
| XM 015151321: Macaca mulatta | XM 023209042: Piliocolobus tephrosceles | 0.002 | -2.928 |
| XM 011759130: Macaca nemestrina | XM 023209042: Piliocolobus tephrosceles | 0.001 | -3.210 |
| XM 007967990: Chlorocebus sabaeus | XM 023209042: Piliocolobus tephrosceles | 0.003 | -2.813 |
| NM 030762 Homo sapiens | XM 025402281: Theropithecus gelada | 0.000 | -5.203 |
| XM 520805: Pan troglodytes | XM 025402281: Theropithecus gelada | 0.000 | -4.862 |
| XM 019037881: Gorilla gorilla gorilla | XM 025402281: Theropithecus gelada | 0.000 | -4.401 |
| XM 002823045: Pongo abelii | XM 025402281: Theropithecus gelada | 0.000 | -5.006 |
| XM 005570417: Macaca fascicularis | XM 025402281: Theropithecus gelada | 0.013 | -2.251 |
| XM 012093655: Cercocebus atys | XM 025402281: Theropithecus gelada | 0.023 | -2.011 |
| XM 015151321: Macaca mulatta | XM 025402281: Theropithecus gelada | 0.012 | -2.301 |
| XM 011759130: Macaca nemestrina | XM 025402281: Theropithecus gelada | 0.004 | -2.671 |
| XM 007967990: Chlorocebus sabaeus | XM 025402281: Theropithecus gelada | 0.006 | -2.568 |
| XM 023209042: Piliocolobus tephrosceles | XM 025402281: Theropithecus gelada | 0.000 | -3.672 |
| NM 030762 Homo sapiens | XM 017507035: Cebus capucinus imitator | 0.000 | -5.701 |
| XM 520805: Pan troglodytes | XM 017507035: Cebus capucinus imitator | 0.000 | -5.382 |
| XM 019037881: Gorilla gorilla gorilla | XM 017507035: Cebus capucinus imitator | 0.000 | -4.589 |
| XM 002823045: Pongo abelii | XM 017507035: Cebus capucinus imitator | 0.000 | -5.309 |
| XM 005570417: Macaca fascicularis | XM 017507035: Cebus capucinus imitator | 0.000 | -5.414 |
| XM 012093655: Cercocebus atys | XM 017507035: Cebus capucinus imitator | 0.000 | -5.521 |
| XM 015151321: Macaca mulatta | XM 017507035: Cebus capucinus imitator | 0.000 | -5.448 |
| XM 011759130: Macaca nemestrina | XM 017507035: Cebus capucinus imitator | 0.000 | -5.627 |
| XM 007967990: Chlorocebus sabaeus | XM 017507035: Cebus capucinus imitator | 0.000 | -5.376 |
| XM 023209042: Piliocolobus tephrosceles | XM 017507035: Cebus capucinus imitator | 0.000 | -5.126 |
| XM 025402281: Theropithecus gelada | XM 017507035: Cebus capucinus imitator | 0.000 | -5.840 |
| NM 030762 Homo sapiens | XM 012739537: Microcebus murinus | 0.000 | -6.070 |
| XM 520805: Pan troglodytes | XM 012739537: Microcebus murinus | 0.000 | -5.770 |
| XM 019037881: Gorilla gorilla gorilla | XM 012739537: Microcebus murinus | 0.000 | -4.892 |
| XM 002823045: Pongo abelii | XM 012739537: Microcebus murinus | 0.000 | -5.712 |
| XM 005570417: Macaca fascicularis | XM 012739537: Microcebus murinus | 0.000 | -5.807 |
| XM 012093655: Cercocebus atys | XM 012739537: Microcebus murinus | 0.000 | -5.908 |
| XM 015151321: Macaca mulatta | XM 012739537: Microcebus murinus | 0.000 | -5.848 |
| XM 011759130: Macaca nemestrina | XM 012739537: Microcebus murinus | 0.000 | -5.928 |
| XM 007967990: Chlorocebus sabaeus | XM 012739537: Microcebus murinus | 0.000 | -5.988 |
| XM 023209042: Piliocolobus tephrosceles | XM 012739537: Microcebus murinus | 0.000 | -6.015 |
| XM 025402281: Theropithecus gelada | XM 012739537: Microcebus murinus | 0.000 | -6.254 |
| XM 017507035: Cebus capucinus imitator | XM 012739537: Microcebus murinus | 0.000 | -6.269 |
| NM 030762 Homo sapiens | XM 007446307: Lipotes vexillifer | 0.000 | -6.273 |
| XM 520805: Pan troglodytes | XM 007446307: Lipotes vexillifer | 0.000 | -5.977 |
| XM 019037881: Gorilla gorilla gorilla | XM 007446307: Lipotes vexillifer | 0.000 | -5.149 |
| XM 002823045: Pongo abelii | XM 007446307: Lipotes vexillifer | 0.000 | -6.118 |
| XM 005570417: Macaca fascicularis | XM 007446307: Lipotes vexillifer | 0.000 | -5.504 |
| XM 012093655: Cercocebus atys | XM 007446307: Lipotes vexillifer | 0.000 | -5.607 |
| XM 015151321: Macaca mulatta | XM 007446307: Lipotes vexillifer | 0.000 | -5.546 |
| XM 011759130: Macaca nemestrina | XM 007446307: Lipotes vexillifer | 0.000 | -5.710 |
| XM 007967990: Chlorocebus sabaeus | XM 007446307: Lipotes vexillifer | 0.000 | -5.691 |
| XM 023209042: Piliocolobus tephrosceles | XM 007446307: Lipotes vexillifer | 0.000 | -5.739 |
| XM 025402281: Theropithecus gelada | XM 007446307: Lipotes vexillifer | 0.000 | -6.081 |
| XM 017507035: Cebus capucinus imitator | XM 007446307: Lipotes vexillifer | 0.000 | -6.428 |
| XM 012739537: Microcebus murinus | XM 007446307: Lipotes vexillifer | 0.000 | -5.357 |
| NM 030762 Homo sapiens | XM 024128992: Physeter catodon | 0.000 | -6.320 |
| XM 520805: Pan troglodytes | XM 024128992: Physeter catodon | 0.000 | -6.024 |
| XM 019037881: Gorilla gorilla gorilla | XM 024128992: Physeter catodon | 0.000 | -5.233 |
| XM 002823045: Pongo abelii | XM 024128992: Physeter catodon | 0.000 | -6.163 |
| XM 005570417: Macaca fascicularis | XM 024128992: Physeter catodon | 0.000 | -5.549 |
| XM 012093655: Cercocebus atys | XM 024128992: Physeter catodon | 0.000 | -5.653 |
| XM 015151321: Macaca mulatta | XM 024128992: Physeter catodon | 0.000 | -5.589 |
| XM 011759130: Macaca nemestrina | XM 024128992: Physeter catodon | 0.000 | -5.756 |
| XM 007967990: Chlorocebus sabaeus | XM 024128992: Physeter catodon | 0.000 | -5.733 |
| XM 023209042: Piliocolobus tephrosceles | XM 024128992: Physeter catodon | 0.000 | -5.715 |
| XM 025402281: Theropithecus gelada | XM 024128992: Physeter catodon | 0.000 | -6.063 |
| XM 017507035: Cebus capucinus imitator | XM 024128992: Physeter catodon | 0.000 | -6.474 |
| XM 012739537: Microcebus murinus | XM 024128992: Physeter catodon | 0.000 | -5.585 |
| XM 007446307: Lipotes vexillifer | XM 024128992: Physeter catodon | 0.194 | -0.867 |
| NM 030762 Homo sapiens | XM 027129408: Lagenorhynchus obliquidens | 0.000 | -6.547 |
| XM 520805: Pan troglodytes | XM 027129408: Lagenorhynchus obliquidens | 0.000 | -6.257 |
| XM 019037881: Gorilla gorilla gorilla | XM 027129408: Lagenorhynchus obliquidens | 0.000 | -5.276 |
| XM 002823045: Pongo abelii | XM 027129408: Lagenorhynchus obliquidens | 0.000 | -6.453 |
| XM 005570417: Macaca fascicularis | XM 027129408: Lagenorhynchus obliquidens | 0.000 | -5.995 |
| XM 012093655: Cercocebus atys | XM 027129408: Lagenorhynchus obliquidens | 0.000 | -6.094 |
| XM 015151321: Macaca mulatta | XM 027129408: Lagenorhynchus obliquidens | 0.000 | -6.033 |
| XM 011759130: Macaca nemestrina | XM 027129408: Lagenorhynchus obliquidens | 0.000 | -6.193 |
| XM 007967990: Chlorocebus sabaeus | XM 027129408: Lagenorhynchus obliquidens | 0.000 | -6.171 |
| XM 023209042: Piliocolobus tephrosceles | XM 027129408: Lagenorhynchus obliquidens | 0.000 | -6.154 |
| XM 025402281: Theropithecus gelada | XM 027129408: Lagenorhynchus obliquidens | 0.000 | -6.490 |
| XM 017507035: Cebus capucinus imitator | XM 027129408: Lagenorhynchus obliquidens | 0.000 | -6.698 |
| XM 012739537: Microcebus murinus | XM 027129408: Lagenorhynchus obliquidens | 0.000 | -5.869 |
| XM 007446307: Lipotes vexillifer | XM 027129408: Lagenorhynchus obliquidens | 0.040 | -1.767 |
| XM 024128992: Physeter catodon | XM 027129408: Lagenorhynchus obliquidens | 0.007 | -2.490 |
| NM 030762 Homo sapiens | XM 025879601: Callorhinus ursinus | 0.000 | -6.643 |
| XM 520805: Pan troglodytes | XM 025879601: Callorhinus ursinus | 0.000 | -6.354 |
| XM 019037881: Gorilla gorilla gorilla | XM 025879601: Callorhinus ursinus | 0.000 | -5.267 |
| XM 002823045: Pongo abelii | XM 025879601: Callorhinus ursinus | 0.000 | -6.197 |
| XM 005570417: Macaca fascicularis | XM 025879601: Callorhinus ursinus | 0.000 | -6.192 |
| XM 012093655: Cercocebus atys | XM 025879601: Callorhinus ursinus | 0.000 | -6.290 |
| XM 015151321: Macaca mulatta | XM 025879601: Callorhinus ursinus | 0.000 | -6.229 |
| XM 011759130: Macaca nemestrina | XM 025879601: Callorhinus ursinus | 0.000 | -6.388 |
| XM 007967990: Chlorocebus sabaeus | XM 025879601: Callorhinus ursinus | 0.000 | -6.069 |
| XM 023209042: Piliocolobus tephrosceles | XM 025879601: Callorhinus ursinus | 0.000 | -6.154 |
| XM 025402281: Theropithecus gelada | XM 025879601: Callorhinus ursinus | 0.000 | -6.396 |
| XM 017507035: Cebus capucinus imitator | XM 025879601: Callorhinus ursinus | 0.000 | -6.702 |
| XM 012739537: Microcebus murinus | XM 025879601: Callorhinus ursinus | 0.000 | -6.711 |
| XM 007446307: Lipotes vexillifer | XM 025879601: Callorhinus ursinus | 0.000 | -4.637 |
| XM 024128992: Physeter catodon | XM 025879601: Callorhinus ursinus | 0.000 | -4.745 |
| XM 027129408: Lagenorhynchus obliquidens | XM 025879601: Callorhinus ursinus | 0.000 | -5.383 |
| NM 030762 Homo sapiens | XM 022577811: Delphinapterus leucas | 0.000 | -6.929 |
| XM 520805: Pan troglodytes | XM 022577811: Delphinapterus leucas | 0.000 | -6.647 |
| XM 019037881: Gorilla gorilla gorilla | XM 022577811: Delphinapterus leucas | 0.000 | -5.826 |
| XM 002823045: Pongo abelii | XM 022577811: Delphinapterus leucas | 0.000 | -6.780 |
| XM 005570417: Macaca fascicularis | XM 022577811: Delphinapterus leucas | 0.000 | -6.198 |
| XM 012093655: Cercocebus atys | XM 022577811: Delphinapterus leucas | 0.000 | -6.296 |
| XM 015151321: Macaca mulatta | XM 022577811: Delphinapterus leucas | 0.000 | -6.236 |
| XM 011759130: Macaca nemestrina | XM 022577811: Delphinapterus leucas | 0.000 | -6.393 |
| XM 007967990: Chlorocebus sabaeus | XM 022577811: Delphinapterus leucas | 0.000 | -6.371 |
| XM 023209042: Piliocolobus tephrosceles | XM 022577811: Delphinapterus leucas | 0.000 | -6.355 |
| XM 025402281: Theropithecus gelada | XM 022577811: Delphinapterus leucas | 0.000 | -6.685 |
| XM 017507035: Cebus capucinus imitator | XM 022577811: Delphinapterus leucas | 0.000 | -7.171 |
| XM 012739537: Microcebus murinus | XM 022577811: Delphinapterus leucas | 0.000 | -6.272 |
| XM 007446307: Lipotes vexillifer | XM 022577811: Delphinapterus leucas | 0.008 | -2.437 |
| XM 024128992: Physeter catodon | XM 022577811: Delphinapterus leucas | 0.001 | -3.044 |
| XM 027129408: Lagenorhynchus obliquidens | XM 022577811: Delphinapterus leucas | 0.007 | -2.492 |
| XM 025879601: Callorhinus ursinus | XM 022577811: Delphinapterus leucas | 0.000 | -5.198 |
| NM 030762 Homo sapiens | XM 019936346: Tursiops truncatus | 0.000 | -6.796 |
| XM 520805: Pan troglodytes | XM 019936346: Tursiops truncatus | 0.000 | -6.511 |
| XM 019037881: Gorilla gorilla gorilla | XM 019936346: Tursiops truncatus | 0.000 | -5.452 |
| XM 002823045: Pongo abelii | XM 019936346: Tursiops truncatus | 0.000 | -6.645 |
| XM 005570417: Macaca fascicularis | XM 019936346: Tursiops truncatus | 0.000 | -6.254 |
| XM 012093655: Cercocebus atys | XM 019936346: Tursiops truncatus | 0.000 | -6.351 |
| XM 015151321: Macaca mulatta | XM 019936346: Tursiops truncatus | 0.000 | -6.291 |
| XM 011759130: Macaca nemestrina | XM 019936346: Tursiops truncatus | 0.000 | -6.448 |
| XM 007967990: Chlorocebus sabaeus | XM 019936346: Tursiops truncatus | 0.000 | -6.425 |
| XM 023209042: Piliocolobus tephrosceles | XM 019936346: Tursiops truncatus | 0.000 | -6.408 |
| XM 025402281: Theropithecus gelada | XM 019936346: Tursiops truncatus | 0.000 | -6.739 |
| XM 017507035: Cebus capucinus imitator | XM 019936346: Tursiops truncatus | 0.000 | -6.945 |
| XM 012739537: Microcebus murinus | XM 019936346: Tursiops truncatus | 0.000 | -6.125 |
| XM 007446307: Lipotes vexillifer | XM 019936346: Tursiops truncatus | 0.010 | -2.358 |
| XM 024128992: Physeter catodon | XM 019936346: Tursiops truncatus | 0.002 | -2.990 |
| XM 027129408: Lagenorhynchus obliquidens | XM 019936346: Tursiops truncatus | 0.130 | -1.132 |
| XM 025879601: Callorhinus ursinus | XM 019936346: Tursiops truncatus | 0.000 | -5.657 |
| XM 022577811: Delphinapterus leucas | XM 019936346: Tursiops truncatus | 0.002 | -2.991 |
| NM 030762 Homo sapiens | XM 027593397: Zalophus californianus | 0.000 | -6.643 |
| XM 520805: Pan troglodytes | XM 027593397: Zalophus californianus | 0.000 | -6.354 |
| XM 019037881: Gorilla gorilla gorilla | XM 027593397: Zalophus californianus | 0.000 | -5.267 |
| XM 002823045: Pongo abelii | XM 027593397: Zalophus californianus | 0.000 | -6.197 |
| XM 005570417: Macaca fascicularis | XM 027593397: Zalophus californianus | 0.000 | -6.192 |
| XM 012093655: Cercocebus atys | XM 027593397: Zalophus californianus | 0.000 | -6.290 |
| XM 015151321: Macaca mulatta | XM 027593397: Zalophus californianus | 0.000 | -6.229 |
| XM 011759130: Macaca nemestrina | XM 027593397: Zalophus californianus | 0.000 | -6.388 |
| XM 007967990: Chlorocebus sabaeus | XM 027593397: Zalophus californianus | 0.000 | -6.069 |
| XM 023209042: Piliocolobus tephrosceles | XM 027593397: Zalophus californianus | 0.000 | -6.154 |
| XM 025402281: Theropithecus gelada | XM 027593397: Zalophus californianus | 0.000 | -6.396 |
| XM 017507035: Cebus capucinus imitator | XM 027593397: Zalophus californianus | 0.000 | -6.702 |
| XM 012739537: Microcebus murinus | XM 027593397: Zalophus californianus | 0.000 | -6.711 |
| XM 007446307: Lipotes vexillifer | XM 027593397: Zalophus californianus | 0.000 | -4.637 |
| XM 024128992: Physeter catodon | XM 027593397: Zalophus californianus | 0.000 | -4.745 |
| XM 027129408: Lagenorhynchus obliquidens | XM 027593397: Zalophus californianus | 0.000 | -5.383 |
| XM 025879601: Callorhinus ursinus | XM 027593397: Zalophus californianus | 1.000 | 0.000 |
| XM 022577811: Delphinapterus leucas | XM 027593397: Zalophus californianus | 0.000 | -5.198 |
| XM 019936346: Tursiops truncatus | XM 027593397: Zalophus californianus | 0.000 | -5.657 |
| NM 030762 Homo sapiens | XM 015093964: Ovis aries | 0.000 | -6.581 |
| XM 520805: Pan troglodytes | XM 015093964: Ovis aries | 0.000 | -6.292 |
| XM 019037881: Gorilla gorilla gorilla | XM 015093964: Ovis aries | 0.000 | -5.434 |
| XM 002823045: Pongo abelii | XM 015093964: Ovis aries | 0.000 | -6.332 |
| XM 005570417: Macaca fascicularis | XM 015093964: Ovis aries | 0.000 | -5.954 |
| XM 012093655: Cercocebus atys | XM 015093964: Ovis aries | 0.000 | -6.054 |
| XM 015151321: Macaca mulatta | XM 015093964: Ovis aries | 0.000 | -5.993 |
| XM 011759130: Macaca nemestrina | XM 015093964: Ovis aries | 0.000 | -6.153 |
| XM 007967990: Chlorocebus sabaeus | XM 015093964: Ovis aries | 0.000 | -6.054 |
| XM 023209042: Piliocolobus tephrosceles | XM 015093964: Ovis aries | 0.000 | -6.218 |
| XM 025402281: Theropithecus gelada | XM 015093964: Ovis aries | 0.000 | -6.457 |
| XM 017507035: Cebus capucinus imitator | XM 015093964: Ovis aries | 0.000 | -6.829 |
| XM 012739537: Microcebus murinus | XM 015093964: Ovis aries | 0.000 | -6.466 |
| XM 007446307: Lipotes vexillifer | XM 015093964: Ovis aries | 0.000 | -4.380 |
| XM 024128992: Physeter catodon | XM 015093964: Ovis aries | 0.000 | -4.355 |
| XM 027129408: Lagenorhynchus obliquidens | XM 015093964: Ovis aries | 0.000 | -4.914 |
| XM 025879601: Callorhinus ursinus | XM 015093964: Ovis aries | 0.000 | -5.683 |
| XM 022577811: Delphinapterus leucas | XM 015093964: Ovis aries | 0.000 | -5.279 |
| XM 019936346: Tursiops truncatus | XM 015093964: Ovis aries | 0.000 | -5.205 |
| XM 027593397: Zalophus californianus | XM 015093964: Ovis aries | 0.000 | -5.683 |
| NM 030762 Homo sapiens | XM 019452268: Panthera pardus | 0.000 | -7.220 |
| XM 520805: Pan troglodytes | XM 019452268: Panthera pardus | 0.000 | -6.944 |
| XM 019037881: Gorilla gorilla gorilla | XM 019452268: Panthera pardus | 0.000 | -6.027 |
| XM 002823045: Pongo abelii | XM 019452268: Panthera pardus | 0.000 | -7.071 |
| XM 005570417: Macaca fascicularis | XM 019452268: Panthera pardus | 0.000 | -6.787 |
| XM 012093655: Cercocebus atys | XM 019452268: Panthera pardus | 0.000 | -6.880 |
| XM 015151321: Macaca mulatta | XM 019452268: Panthera pardus | 0.000 | -6.819 |
| XM 011759130: Macaca nemestrina | XM 019452268: Panthera pardus | 0.000 | -6.974 |
| XM 007967990: Chlorocebus sabaeus | XM 019452268: Panthera pardus | 0.000 | -6.664 |
| XM 023209042: Piliocolobus tephrosceles | XM 019452268: Panthera pardus | 0.000 | -6.738 |
| XM 025402281: Theropithecus gelada | XM 019452268: Panthera pardus | 0.000 | -7.256 |
| XM 017507035: Cebus capucinus imitator | XM 019452268: Panthera pardus | 0.000 | -7.460 |
| XM 012739537: Microcebus murinus | XM 019452268: Panthera pardus | 0.000 | -7.237 |
| XM 007446307: Lipotes vexillifer | XM 019452268: Panthera pardus | 0.000 | -5.214 |
| XM 024128992: Physeter catodon | XM 019452268: Panthera pardus | 0.000 | -5.488 |
| XM 027129408: Lagenorhynchus obliquidens | XM 019452268: Panthera pardus | 0.000 | -5.871 |
| XM 025879601: Callorhinus ursinus | XM 019452268: Panthera pardus | 0.000 | -5.016 |
| XM 022577811: Delphinapterus leucas | XM 019452268: Panthera pardus | 0.000 | -5.877 |
| XM 019936346: Tursiops truncatus | XM 019452268: Panthera pardus | 0.000 | -6.137 |
| XM 027593397: Zalophus californianus | XM 019452268: Panthera pardus | 0.000 | -5.016 |
| XM 015093964: Ovis aries | XM 019452268: Panthera pardus | 0.000 | -5.976 |
| NM 030762 Homo sapiens | XM 004270956: Orcinus orca | 0.000 | -6.554 |
| XM 520805: Pan troglodytes | XM 004270956: Orcinus orca | 0.000 | -6.264 |
| XM 019037881: Gorilla gorilla gorilla | XM 004270956: Orcinus orca | 0.000 | -5.452 |
| XM 002823045: Pongo abelii | XM 004270956: Orcinus orca | 0.000 | -6.401 |
| XM 005570417: Macaca fascicularis | XM 004270956: Orcinus orca | 0.000 | -6.101 |
| XM 012093655: Cercocebus atys | XM 004270956: Orcinus orca | 0.000 | -6.200 |
| XM 015151321: Macaca mulatta | XM 004270956: Orcinus orca | 0.000 | -6.139 |
| XM 011759130: Macaca nemestrina | XM 004270956: Orcinus orca | 0.000 | -6.298 |
| XM 007967990: Chlorocebus sabaeus | XM 004270956: Orcinus orca | 0.000 | -6.276 |
| XM 023209042: Piliocolobus tephrosceles | XM 004270956: Orcinus orca | 0.000 | -6.260 |
| XM 025402281: Theropithecus gelada | XM 004270956: Orcinus orca | 0.000 | -6.593 |
| XM 017507035: Cebus capucinus imitator | XM 004270956: Orcinus orca | 0.000 | -6.893 |
| XM 012739537: Microcebus murinus | XM 004270956: Orcinus orca | 0.000 | -6.075 |
| XM 007446307: Lipotes vexillifer | XM 004270956: Orcinus orca | 0.008 | -2.436 |
| XM 024128992: Physeter catodon | XM 004270956: Orcinus orca | 0.001 | -3.043 |
| XM 027129408: Lagenorhynchus obliquidens | XM 004270956: Orcinus orca | 0.102 | -1.278 |
| XM 025879601: Callorhinus ursinus | XM 004270956: Orcinus orca | 0.000 | -5.493 |
| XM 022577811: Delphinapterus leucas | XM 004270956: Orcinus orca | 0.004 | -2.688 |
| XM 019936346: Tursiops truncatus | XM 004270956: Orcinus orca | 0.037 | -1.805 |
| XM 027593397: Zalophus californianus | XM 004270956: Orcinus orca | 0.000 | -5.493 |
| XM 015093964: Ovis aries | XM 004270956: Orcinus orca | 0.000 | -5.142 |
| XM 019452268: Panthera pardus | XM 004270956: Orcinus orca | 0.000 | -5.976 |
| NM 030762 Homo sapiens | XM 027541573: Bos indicus x Bos taurus | 0.000 | -7.211 |
| XM 520805: Pan troglodytes | XM 027541573: Bos indicus x Bos taurus | 0.000 | -6.935 |
| XM 019037881: Gorilla gorilla gorilla | XM 027541573: Bos indicus x Bos taurus | 0.000 | -5.586 |
| XM 002823045: Pongo abelii | XM 027541573: Bos indicus x Bos taurus | 0.000 | -6.974 |
| XM 005570417: Macaca fascicularis | XM 027541573: Bos indicus x Bos taurus | 0.000 | -6.402 |
| XM 012093655: Cercocebus atys | XM 027541573: Bos indicus x Bos taurus | 0.000 | -6.498 |
| XM 015151321: Macaca mulatta | XM 027541573: Bos indicus x Bos taurus | 0.000 | -6.440 |
| XM 011759130: Macaca nemestrina | XM 027541573: Bos indicus x Bos taurus | 0.000 | -6.593 |
| XM 007967990: Chlorocebus sabaeus | XM 027541573: Bos indicus x Bos taurus | 0.000 | -6.498 |
| XM 023209042: Piliocolobus tephrosceles | XM 027541573: Bos indicus x Bos taurus | 0.000 | -6.716 |
| XM 025402281: Theropithecus gelada | XM 027541573: Bos indicus x Bos taurus | 0.000 | -6.944 |
| XM 017507035: Cebus capucinus imitator | XM 027541573: Bos indicus x Bos taurus | 0.000 | -7.503 |
| XM 012739537: Microcebus murinus | XM 027541573: Bos indicus x Bos taurus | 0.000 | -6.843 |
| XM 007446307: Lipotes vexillifer | XM 027541573: Bos indicus x Bos taurus | 0.000 | -5.003 |
| XM 024128992: Physeter catodon | XM 027541573: Bos indicus x Bos taurus | 0.000 | -4.983 |
| XM 027129408: Lagenorhynchus obliquidens | XM 027541573: Bos indicus x Bos taurus | 0.000 | -5.392 |
| XM 025879601: Callorhinus ursinus | XM 027541573: Bos indicus x Bos taurus | 0.000 | -6.298 |
| XM 022577811: Delphinapterus leucas | XM 027541573: Bos indicus x Bos taurus | 0.000 | -5.609 |
| XM 019936346: Tursiops truncatus | XM 027541573: Bos indicus x Bos taurus | 0.000 | -5.563 |
| XM 027593397: Zalophus californianus | XM 027541573: Bos indicus x Bos taurus | 0.000 | -6.298 |
| XM 015093964: Ovis aries | XM 027541573: Bos indicus x Bos taurus | 0.000 | -3.610 |
| XM 019452268: Panthera pardus | XM 027541573: Bos indicus x Bos taurus | 0.000 | -6.693 |
| XM 004270956: Orcinus orca | XM 027541573: Bos indicus x Bos taurus | 0.000 | -5.504 |
| NM 030762 Homo sapiens | XM 027934162: Marmota flaviventris | 0.000 | -8.201 |
| XM 520805: Pan troglodytes | XM 027934162: Marmota flaviventris | 0.000 | -7.939 |
| XM 019037881: Gorilla gorilla gorilla | XM 027934162: Marmota flaviventris | 0.000 | -6.800 |
| XM 002823045: Pongo abelii | XM 027934162: Marmota flaviventris | 0.000 | -8.204 |
| XM 005570417: Macaca fascicularis | XM 027934162: Marmota flaviventris | 0.000 | -8.143 |
| XM 012093655: Cercocebus atys | XM 027934162: Marmota flaviventris | 0.000 | -8.230 |
| XM 015151321: Macaca mulatta | XM 027934162: Marmota flaviventris | 0.000 | -8.175 |
| XM 011759130: Macaca nemestrina | XM 027934162: Marmota flaviventris | 0.000 | -8.246 |
| XM 007967990: Chlorocebus sabaeus | XM 027934162: Marmota flaviventris | 0.000 | -8.121 |
| XM 023209042: Piliocolobus tephrosceles | XM 027934162: Marmota flaviventris | 0.000 | -7.303 |
| XM 025402281: Theropithecus gelada | XM 027934162: Marmota flaviventris | 0.000 | -7.973 |
| XM 017507035: Cebus capucinus imitator | XM 027934162: Marmota flaviventris | 0.000 | -8.341 |
| XM 012739537: Microcebus murinus | XM 027934162: Marmota flaviventris | 0.000 | -8.809 |
| XM 007446307: Lipotes vexillifer | XM 027934162: Marmota flaviventris | 0.000 | -8.176 |
| XM 024128992: Physeter catodon | XM 027934162: Marmota flaviventris | 0.000 | -8.215 |
| XM 027129408: Lagenorhynchus obliquidens | XM 027934162: Marmota flaviventris | 0.000 | -8.472 |
| XM 025879601: Callorhinus ursinus | XM 027934162: Marmota flaviventris | 0.000 | -7.797 |
| XM 022577811: Delphinapterus leucas | XM 027934162: Marmota flaviventris | 0.000 | -8.717 |
| XM 019936346: Tursiops truncatus | XM 027934162: Marmota flaviventris | 0.000 | -8.590 |
| XM 027593397: Zalophus californianus | XM 027934162: Marmota flaviventris | 0.000 | -7.797 |
| XM 015093964: Ovis aries | XM 027934162: Marmota flaviventris | 0.000 | -8.628 |
| XM 019452268: Panthera pardus | XM 027934162: Marmota flaviventris | 0.000 | -8.750 |
| XM 004270956: Orcinus orca | XM 027934162: Marmota flaviventris | 0.000 | -8.544 |
| XM 027541573: Bos indicus x Bos taurus | XM 027934162: Marmota flaviventris | 0.000 | -9.254 |
| NM 030762 Homo sapiens | XM 003355541: Sus scrofa | 0.000 | -8.612 |
| XM 520805: Pan troglodytes | XM 003355541: Sus scrofa | 0.000 | -8.354 |
| XM 019037881: Gorilla gorilla gorilla | XM 003355541: Sus scrofa | 0.000 | -7.409 |
| XM 002823045: Pongo abelii | XM 003355541: Sus scrofa | 0.000 | -8.130 |
| XM 005570417: Macaca fascicularis | XM 003355541: Sus scrofa | 0.000 | -7.900 |
| XM 012093655: Cercocebus atys | XM 003355541: Sus scrofa | 0.000 | -7.988 |
| XM 015151321: Macaca mulatta | XM 003355541: Sus scrofa | 0.000 | -7.933 |
| XM 011759130: Macaca nemestrina | XM 003355541: Sus scrofa | 0.000 | -8.076 |
| XM 007967990: Chlorocebus sabaeus | XM 003355541: Sus scrofa | 0.000 | -8.055 |
| XM 023209042: Piliocolobus tephrosceles | XM 003355541: Sus scrofa | 0.000 | -7.828 |
| XM 025402281: Theropithecus gelada | XM 003355541: Sus scrofa | 0.000 | -8.172 |
| XM 017507035: Cebus capucinus imitator | XM 003355541: Sus scrofa | 0.000 | -8.833 |
| XM 012739537: Microcebus murinus | XM 003355541: Sus scrofa | 0.000 | -8.607 |
| XM 007446307: Lipotes vexillifer | XM 003355541: Sus scrofa | 0.000 | -6.802 |
| XM 024128992: Physeter catodon | XM 003355541: Sus scrofa | 0.000 | -6.943 |
| XM 027129408: Lagenorhynchus obliquidens | XM 003355541: Sus scrofa | 0.000 | -7.343 |
| XM 025879601: Callorhinus ursinus | XM 003355541: Sus scrofa | 0.000 | -7.652 |
| XM 022577811: Delphinapterus leucas | XM 003355541: Sus scrofa | 0.000 | -7.367 |
| XM 019936346: Tursiops truncatus | XM 003355541: Sus scrofa | 0.000 | -7.401 |
| XM 027593397: Zalophus californianus | XM 003355541: Sus scrofa | 0.000 | -7.652 |
| XM 015093964: Ovis aries | XM 003355541: Sus scrofa | 0.000 | -7.579 |
| XM 019452268: Panthera pardus | XM 003355541: Sus scrofa | 0.000 | -8.071 |
| XM 004270956: Orcinus orca | XM 003355541: Sus scrofa | 0.000 | -7.351 |
| XM 027541573: Bos indicus x Bos taurus | XM 003355541: Sus scrofa | 0.000 | -7.788 |
| XM 027934162: Marmota flaviventris | XM 003355541: Sus scrofa | 0.000 | -9.777 |
| NM 030762 Homo sapiens | XM 016119294: Rousettus aegyptiacus | 0.000 | -7.992 |
| XM 520805: Pan troglodytes | XM 016119294: Rousettus aegyptiacus | 0.000 | -7.904 |
| XM 019037881: Gorilla gorilla gorilla | XM 016119294: Rousettus aegyptiacus | 0.000 | -7.153 |
| XM 002823045: Pongo abelii | XM 016119294: Rousettus aegyptiacus | 0.000 | -7.995 |
| XM 005570417: Macaca fascicularis | XM 016119294: Rousettus aegyptiacus | 0.000 | -7.934 |
| XM 012093655: Cercocebus atys | XM 016119294: Rousettus aegyptiacus | 0.000 | -8.022 |
| XM 015151321: Macaca mulatta | XM 016119294: Rousettus aegyptiacus | 0.000 | -8.004 |
| XM 011759130: Macaca nemestrina | XM 016119294: Rousettus aegyptiacus | 0.000 | -8.110 |
| XM 007967990: Chlorocebus sabaeus | XM 016119294: Rousettus aegyptiacus | 0.000 | -8.022 |
| XM 023209042: Piliocolobus tephrosceles | XM 016119294: Rousettus aegyptiacus | 0.000 | -7.899 |
| XM 025402281: Theropithecus gelada | XM 016119294: Rousettus aegyptiacus | 0.000 | -8.116 |
| XM 017507035: Cebus capucinus imitator | XM 016119294: Rousettus aegyptiacus | 0.000 | -8.028 |
| XM 012739537: Microcebus murinus | XM 016119294: Rousettus aegyptiacus | 0.000 | -8.919 |
| XM 007446307: Lipotes vexillifer | XM 016119294: Rousettus aegyptiacus | 0.000 | -7.781 |
| XM 024128992: Physeter catodon | XM 016119294: Rousettus aegyptiacus | 0.000 | -7.820 |
| XM 027129408: Lagenorhynchus obliquidens | XM 016119294: Rousettus aegyptiacus | 0.000 | -8.201 |
| XM 025879601: Callorhinus ursinus | XM 016119294: Rousettus aegyptiacus | 0.000 | -7.905 |
| XM 022577811: Delphinapterus leucas | XM 016119294: Rousettus aegyptiacus | 0.000 | -7.981 |
| XM 019936346: Tursiops truncatus | XM 016119294: Rousettus aegyptiacus | 0.000 | -8.375 |
| XM 027593397: Zalophus californianus | XM 016119294: Rousettus aegyptiacus | 0.000 | -7.905 |
| XM 015093964: Ovis aries | XM 016119294: Rousettus aegyptiacus | 0.000 | -8.502 |
| XM 019452268: Panthera pardus | XM 016119294: Rousettus aegyptiacus | 0.000 | -8.488 |
| XM 004270956: Orcinus orca | XM 016119294: Rousettus aegyptiacus | 0.000 | -8.241 |
| XM 027541573: Bos indicus x Bos taurus | XM 016119294: Rousettus aegyptiacus | 0.000 | -9.044 |
| XM 027934162: Marmota flaviventris | XM 016119294: Rousettus aegyptiacus | 0.000 | -10.044 |
| XM 003355541: Sus scrofa | XM 016119294: Rousettus aegyptiacus | 0.000 | -10.189 |
| NM 030762 Homo sapiens | XM 006127674: Pelodiscus sinensis | 0.000 | -8.791 |
| XM 520805: Pan troglodytes | XM 006127674: Pelodiscus sinensis | 0.000 | -8.698 |
| XM 019037881: Gorilla gorilla gorilla | XM 006127674: Pelodiscus sinensis | 0.000 | -8.428 |
| XM 002823045: Pongo abelii | XM 006127674: Pelodiscus sinensis | 0.000 | -8.769 |
| XM 005570417: Macaca fascicularis | XM 006127674: Pelodiscus sinensis | 0.000 | -8.908 |
| XM 012093655: Cercocebus atys | XM 006127674: Pelodiscus sinensis | 0.000 | -9.002 |
| XM 015151321: Macaca mulatta | XM 006127674: Pelodiscus sinensis | 0.000 | -8.908 |
| XM 011759130: Macaca nemestrina | XM 006127674: Pelodiscus sinensis | 0.000 | -9.096 |
| XM 007967990: Chlorocebus sabaeus | XM 006127674: Pelodiscus sinensis | 0.000 | -9.002 |
| XM 023209042: Piliocolobus tephrosceles | XM 006127674: Pelodiscus sinensis | 0.000 | -9.093 |
| XM 025402281: Theropithecus gelada | XM 006127674: Pelodiscus sinensis | 0.000 | -9.408 |
| XM 017507035: Cebus capucinus imitator | XM 006127674: Pelodiscus sinensis | 0.000 | -8.459 |
| XM 012739537: Microcebus murinus | XM 006127674: Pelodiscus sinensis | 0.000 | -8.924 |
| XM 007446307: Lipotes vexillifer | XM 006127674: Pelodiscus sinensis | 0.000 | -9.133 |
| XM 024128992: Physeter catodon | XM 006127674: Pelodiscus sinensis | 0.000 | -9.139 |
| XM 027129408: Lagenorhynchus obliquidens | XM 006127674: Pelodiscus sinensis | 0.000 | -9.375 |
| XM 025879601: Callorhinus ursinus | XM 006127674: Pelodiscus sinensis | 0.000 | -9.307 |
| XM 022577811: Delphinapterus leucas | XM 006127674: Pelodiscus sinensis | 0.000 | -9.222 |
| XM 019936346: Tursiops truncatus | XM 006127674: Pelodiscus sinensis | 0.000 | -9.507 |
| XM 027593397: Zalophus californianus | XM 006127674: Pelodiscus sinensis | 0.000 | -9.307 |
| XM 015093964: Ovis aries | XM 006127674: Pelodiscus sinensis | 0.000 | -9.615 |
| XM 019452268: Panthera pardus | XM 006127674: Pelodiscus sinensis | 0.000 | -10.083 |
| XM 004270956: Orcinus orca | XM 006127674: Pelodiscus sinensis | 0.000 | -9.387 |
| XM 027541573: Bos indicus x Bos taurus | XM 006127674: Pelodiscus sinensis | 0.000 | -9.817 |
| XM 027934162: Marmota flaviventris | XM 006127674: Pelodiscus sinensis | 0.000 | -10.341 |
| XM 003355541: Sus scrofa | XM 006127674: Pelodiscus sinensis | 0.000 | -10.254 |
| XM 016119294: Rousettus aegyptiacus | XM 006127674: Pelodiscus sinensis | 0.000 | -10.190 |
