# Supplementary material for: Unexpected predicted length variation for the coding sequence of the sleep related gene, BHLHE41 in gorilla amidst strong purifying selection across mammals
Source: PLoS One. 2020 Apr 14;15(4):e0223203. doi: 10.1371/journal.pone.0223203 (PMC7156063; doi:10.1371/journal.pone.0223203)
Supplement: S2 Table — (DOCX) [file pone.0223203.s005.docx]

S2 Table. Codon-by-codon test for selection. P-value is reported as significance to reject the null hypothesis of neutrality (dN=dS). When no synonymous (s) and non-synonymous (n) substitutions are observed, the p-value cannot be calculated (N/A). Codons where >70% of the sequences have gaps have been removed (e.g., codons starting at bp site 1-318 are only present in gorilla and have been removed).

| Codon | Codon start (bp) | Triplet | Syn (s) | Nonsyn (n) | Syn sites (S) | Nonsyn sites (N) | dS | dN | dN-dS | P-value |
| --- | --- | --- | --- | --- | --- | --- | --- | --- | --- | --- |
| 1 | 319 | AUG | 0 | 0 | 0.00 | 3.00 | 0.00 | 0.00 | 0.00 | N/A |
| 2 | 322 | GAC | 1 | 0 | 0.49 | 2.51 | 2.02 | 0.00 | -2.02 | 0.00 |
| 3 | 325 | GAA | 0 | 0 | 0.64 | 2.18 | 0.00 | 0.00 | 0.00 | N/A |
| 4 | 328 | GGA | 0 | 0 | 1.00 | 1.82 | 0.00 | 0.00 | 0.00 | N/A |
| 5 | 331 | AUU | 1 | 0 | 0.67 | 2.33 | 1.50 | 0.00 | -1.50 | 0.00 |
| 6 | 334 | CCU | 0 | 0 | 1.00 | 2.00 | 0.00 | 0.00 | 0.00 | N/A |
| 7 | 337 | CAU | 0 | 1 | 0.70 | 2.30 | 0.00 | 0.43 | 0.43 | 0.23 |
| 8 | 340 | UUG | 0 | 0 | 1.15 | 1.79 | 0.00 | 0.00 | 0.00 | N/A |
| 9 | 343 | CAA | 0 | 1 | 0.71 | 1.93 | 0.00 | 0.52 | 0.52 | 0.27 |
| 10 | 346 | GAG | 1 | 1 | 0.52 | 2.34 | 1.92 | 0.43 | -1.49 | 0.03 |
| 11 | 349 | AGA | 1 | 0 | 0.93 | 2.03 | 1.07 | 0.00 | -1.07 | 0.00 |
| 12 | 352 | CAG | 1 | 0 | 0.53 | 2.01 | 1.88 | 0.00 | -1.88 | 0.00 |
| 13 | 355 | UUA | 1 | 0 | 1.23 | 1.46 | 0.81 | 0.00 | -0.81 | 0.00 |
| 14 | 358 | CUG | 1 | 0 | 1.46 | 1.54 | 0.68 | 0.00 | -0.68 | 0.00 |
| 15 | 361 | GAA | 0 | 0 | 0.64 | 2.18 | 0.00 | 0.00 | 0.00 | N/A |
| 16 | 364 | CAU | 1 | 0 | 0.61 | 2.39 | 1.63 | 0.00 | -1.63 | 0.00 |
| 17 | 367 | AGA | 1 | 2 | 0.91 | 2.06 | 0.92 | 1.05 | 0.13 | 0.30 |
| 18 | 370 | GAU | 0 | 0 | 0.62 | 2.38 | 0.00 | 0.00 | 0.00 | N/A |
| 19 | 373 | UUU | 0 | 0 | 0.62 | 2.38 | 0.00 | 0.00 | 0.00 | N/A |
| 20 | 376 | AUA | 0 | 0 | 0.36 | 2.64 | 0.00 | 0.00 | 0.00 | N/A |
| 21 | 379 | GGA | 0 | 0 | 1.00 | 1.82 | 0.00 | 0.00 | 0.00 | N/A |
| 22 | 382 | CUG | 0 | 0 | 1.46 | 1.54 | 0.00 | 0.00 | 0.00 | N/A |
| 23 | 385 | GAC | 3 | 0 | 0.49 | 2.51 | 6.10 | 0.00 | -6.10 | 0.00 |
| 24 | 388 | UAU | 0 | 0 | 0.62 | 2.00 | 0.00 | 0.00 | 0.00 | N/A |
| 25 | 391 | UCC | 2 | 2 | 1.00 | 1.96 | 2.00 | 1.02 | -0.98 | 0.12 |
| 26 | 394 | UCU | 2 | 1 | 1.00 | 2.00 | 2.00 | 0.50 | -1.50 | 0.04 |
| 27 | 397 | UUG | 0 | 0 | 1.15 | 1.79 | 0.00 | 0.00 | 0.00 | N/A |
| 28 | 400 | UAU | 0 | 0 | 0.62 | 2.00 | 0.00 | 0.00 | 0.00 | N/A |
| 29 | 403 | AUG | 0 | 1 | 0.25 | 2.74 | 0.00 | 0.37 | 0.37 | 0.08 |
| 30 | 406 | UGU | 2 | 0 | 0.58 | 2.33 | 3.47 | 0.00 | -3.47 | 0.00 |
| 31 | 409 | AAA | 1 | 0 | 0.61 | 2.34 | 1.64 | 0.00 | -1.64 | 0.00 |
| 32 | 412 | CCC | 1 | 0 | 1.00 | 2.00 | 1.00 | 0.00 | -1.00 | 0.00 |
| 33 | 415 | AAA | 0 | 0 | 0.64 | 2.32 | 0.00 | 0.00 | 0.00 | N/A |
| 34 | 418 | AGG | 1 | 0 | 0.88 | 2.11 | 1.14 | 0.00 | -1.14 | 0.00 |
| 35 | 421 | AGC | 1 | 1 | 0.58 | 2.42 | 1.73 | 0.41 | -1.32 | 0.04 |
| 36 | 424 | AUG | 0 | 0 | 0.00 | 3.00 | 0.00 | 0.00 | 0.00 | N/A |
| 37 | 427 | AAA | 2 | 0 | 0.60 | 2.36 | 3.34 | 0.00 | -3.34 | 0.00 |
| 38 | 430 | CGA | 1 | 0 | 1.12 | 1.51 | 0.90 | 0.00 | -0.90 | 0.00 |
| 39 | 433 | GAC | 2 | 0 | 0.50 | 2.50 | 4.00 | 0.00 | -4.00 | 0.00 |
| 40 | 436 | GAC | 3 | 1 | 0.53 | 2.43 | 5.67 | 0.41 | -5.26 | 0.00 |
| 41 | 439 | ACC | 1 | 1 | 0.55 | 2.45 | 1.80 | 0.41 | -1.39 | 0.03 |
| 42 | 442 | AAG | 0 | 0 | 0.53 | 2.42 | 0.00 | 0.00 | 0.00 | N/A |
| 43 | 445 | GAU | 0 | 1 | 0.62 | 2.34 | 0.00 | 0.43 | 0.43 | 0.21 |
| 44 | 448 | ACC | 1 | 0 | 1.00 | 2.00 | 1.00 | 0.00 | -1.00 | 0.00 |
| 45 | 451 | UAC | 0 | 0 | 0.46 | 2.00 | 0.00 | 0.00 | 0.00 | N/A |
| 46 | 454 | AAA | 1 | 0 | 0.63 | 2.32 | 1.58 | 0.00 | -1.58 | 0.00 |
| 47 | 457 | UUA | 2 | 0 | 1.29 | 1.43 | 1.55 | 0.00 | -1.55 | 0.00 |
| 48 | 460 | CCG | 2 | 0 | 1.00 | 2.00 | 2.00 | 0.00 | -2.00 | 0.00 |
| 49 | 463 | CAC | 1 | 0 | 0.49 | 2.51 | 2.02 | 0.00 | -2.02 | 0.00 |
| 50 | 466 | AGA | 0 | 0 | 0.96 | 2.00 | 0.00 | 0.00 | 0.00 | N/A |
| 51 | 469 | UUA | 2 | 0 | 1.29 | 1.43 | 1.55 | 0.00 | -1.55 | 0.00 |
| 52 | 472 | AUA | 0 | 0 | 0.36 | 2.64 | 0.00 | 0.00 | 0.00 | N/A |
| 53 | 475 | GAA | 0 | 0 | 0.64 | 2.18 | 0.00 | 0.00 | 0.00 | N/A |
| 54 | 478 | AAG | 0 | 0 | 0.53 | 2.42 | 0.00 | 0.00 | 0.00 | N/A |
| 55 | 481 | AAA | 1 | 0 | 0.61 | 2.34 | 1.63 | 0.00 | -1.63 | 0.00 |
| 56 | 484 | AGA | 1 | 0 | 0.93 | 2.03 | 1.07 | 0.00 | -1.07 | 0.00 |
| 57 | 487 | AGA | 1 | 0 | 1.00 | 1.87 | 1.00 | 0.00 | -1.00 | 0.00 |
| 58 | 490 | GAC | 1 | 0 | 0.47 | 2.53 | 2.14 | 0.00 | -2.14 | 0.00 |
| 59 | 493 | CGA | 2 | 0 | 1.10 | 1.57 | 1.82 | 0.00 | -1.82 | 0.00 |
| 60 | 496 | AUU | 1 | 0 | 0.61 | 2.39 | 1.63 | 0.00 | -1.63 | 0.00 |
| 61 | 499 | AAU | 0 | 0 | 0.62 | 2.38 | 0.00 | 0.00 | 0.00 | N/A |
| 62 | 502 | GAA | 0 | 0 | 0.64 | 2.18 | 0.00 | 0.00 | 0.00 | N/A |
| 63 | 505 | UGC | 0 | 0 | 0.46 | 2.38 | 0.00 | 0.00 | 0.00 | N/A |
| 64 | 508 | AUU | 1 | 0 | 0.68 | 2.32 | 1.47 | 0.00 | -1.47 | 0.00 |
| 65 | 511 | GCU | 0 | 0 | 1.00 | 2.00 | 0.00 | 0.00 | 0.00 | N/A |
| 66 | 514 | CAG | 0 | 0 | 0.53 | 2.01 | 0.00 | 0.00 | 0.00 | N/A |
| 67 | 517 | CUG | 2 | 0 | 1.46 | 1.54 | 1.37 | 0.00 | -1.37 | 0.00 |
| 68 | 520 | AAA | 0 | 0 | 0.64 | 2.32 | 0.00 | 0.00 | 0.00 | N/A |
| 69 | 523 | GAU | 1 | 0 | 0.59 | 2.41 | 1.70 | 0.00 | -1.70 | 0.00 |
| 70 | 526 | UUA | 0 | 0 | 1.25 | 1.36 | 0.00 | 0.00 | 0.00 | N/A |
| 71 | 529 | CUG | 1 | 0 | 1.39 | 1.59 | 0.72 | 0.00 | -0.72 | 0.00 |
| 72 | 532 | CCU | 0 | 0 | 1.00 | 2.00 | 0.00 | 0.00 | 0.00 | N/A |
| 73 | 535 | GAA | 1 | 0 | 0.61 | 2.21 | 1.63 | 0.00 | -1.63 | 0.00 |
| 74 | 538 | CAU | 0 | 0 | 0.62 | 2.38 | 0.00 | 0.00 | 0.00 | N/A |
| 75 | 541 | CUG | 1 | 0 | 1.46 | 1.54 | 0.68 | 0.00 | -0.68 | 0.00 |
| 76 | 544 | AAA | 1 | 0 | 0.60 | 2.35 | 1.66 | 0.00 | -1.66 | 0.00 |
| 77 | 547 | UUG | 1 | 0 | 1.15 | 1.78 | 0.87 | 0.00 | -0.87 | 0.00 |
| 78 | 550 | ACA | 1 | 0 | 1.00 | 2.00 | 1.00 | 0.00 | -1.00 | 0.00 |
| 79 | 553 | ACU | 1 | 0 | 1.00 | 2.00 | 1.00 | 0.00 | -1.00 | 0.00 |
| 80 | 556 | CUG | 1 | 0 | 1.46 | 1.54 | 0.68 | 0.00 | -0.68 | 0.00 |
| 81 | 559 | GGA | 1 | 0 | 1.00 | 2.00 | 1.00 | 0.00 | -1.00 | 0.00 |
| 82 | 562 | CAU | 1 | 0 | 0.61 | 2.39 | 1.64 | 0.00 | -1.64 | 0.00 |
| 83 | 565 | CUG | 0 | 0 | 1.46 | 1.54 | 0.00 | 0.00 | 0.00 | N/A |
| 84 | 568 | GAG | 0 | 0 | 0.53 | 2.29 | 0.00 | 0.00 | 0.00 | N/A |
| 85 | 571 | AAA | 0 | 0 | 0.64 | 2.32 | 0.00 | 0.00 | 0.00 | N/A |
| 86 | 574 | GCU | 5 | 0 | 1.00 | 2.00 | 5.00 | 0.00 | -5.00 | 0.00 |
| 87 | 577 | GUA | 2 | 0 | 1.00 | 2.00 | 2.00 | 0.00 | -2.00 | 0.00 |
| 88 | 580 | GUC | 3 | 0 | 1.00 | 2.00 | 3.00 | 0.00 | -3.00 | 0.00 |
| 89 | 583 | UUG | 1 | 0 | 1.16 | 1.78 | 0.86 | 0.00 | -0.86 | 0.00 |
| 90 | 586 | GAA | 0 | 0 | 0.64 | 2.18 | 0.00 | 0.00 | 0.00 | N/A |
| 91 | 589 | UUA | 1 | 0 | 1.25 | 1.38 | 0.80 | 0.00 | -0.80 | 0.00 |
| 92 | 592 | ACU | 0 | 0 | 1.00 | 2.00 | 0.00 | 0.00 | 0.00 | N/A |
| 93 | 595 | UUG | 1 | 0 | 1.16 | 1.78 | 0.86 | 0.00 | -0.86 | 0.00 |
| 94 | 598 | AAA | 0 | 0 | 0.64 | 2.32 | 0.00 | 0.00 | 0.00 | N/A |
| 95 | 601 | CAC | 0 | 0 | 0.46 | 2.54 | 0.00 | 0.00 | 0.00 | N/A |
| 96 | 604 | UUA | 1 | 0 | 1.25 | 1.37 | 0.80 | 0.00 | -0.80 | 0.00 |
| 97 | 607 | AAA | 0 | 0 | 0.64 | 2.32 | 0.00 | 0.00 | 0.00 | N/A |
| 98 | 610 | GCU | 0 | 0 | 1.00 | 2.00 | 0.00 | 0.00 | 0.00 | N/A |
| 99 | 613 | UUA | 1 | 0 | 1.25 | 1.38 | 0.80 | 0.00 | -0.80 | 0.00 |
| 100 | 616 | ACC | 2 | 0 | 1.00 | 2.00 | 2.00 | 0.00 | -2.00 | 0.00 |
| 101 | 619 | GCC | 0 | 0 | 1.00 | 2.00 | 0.00 | 0.00 | 0.00 | N/A |
| 102 | 622 | UUA | 0 | 0 | 1.25 | 1.36 | 0.00 | 0.00 | 0.00 | N/A |
| 103 | 625 | ACC | 3 | 0 | 1.00 | 2.00 | 3.00 | 0.00 | -3.00 | 0.00 |
| 104 | 628 | GAG | 0 | 0 | 0.53 | 2.29 | 0.00 | 0.00 | 0.00 | N/A |
| 105 | 631 | CAA | 3 | 0 | 0.57 | 1.97 | 5.30 | 0.00 | -5.30 | 0.00 |
| 106 | 634 | CAG | 1 | 0 | 0.54 | 2.00 | 1.86 | 0.00 | -1.86 | 0.00 |
| 107 | 637 | CAU | 1 | 0 | 0.61 | 2.39 | 1.63 | 0.00 | -1.63 | 0.00 |
| 108 | 640 | CAG | 0 | 0 | 0.53 | 2.01 | 0.00 | 0.00 | 0.00 | N/A |
| 109 | 643 | AAG | 0 | 1 | 0.55 | 2.41 | 0.00 | 0.41 | 0.41 | 0.19 |
| 110 | 646 | AUA | 0 | 0 | 0.36 | 2.64 | 0.00 | 0.00 | 0.00 | N/A |
| 111 | 649 | AUU | 0 | 0 | 0.68 | 2.32 | 0.00 | 0.00 | 0.00 | N/A |
| 112 | 652 | GCU | 0 | 0 | 1.00 | 2.00 | 0.00 | 0.00 | 0.00 | N/A |
| 113 | 655 | UUA | 0 | 0 | 1.25 | 1.36 | 0.00 | 0.00 | 0.00 | N/A |
| 114 | 658 | CAG | 0 | 0 | 0.53 | 2.01 | 0.00 | 0.00 | 0.00 | N/A |
| 115 | 661 | AAU | 0 | 0 | 0.62 | 2.38 | 0.00 | 0.00 | 0.00 | N/A |
| 116 | 664 | GGG | 0 | 0 | 1.00 | 2.00 | 0.00 | 0.00 | 0.00 | N/A |
| 117 | 667 | GAG | 1 | 0 | 0.53 | 2.29 | 1.87 | 0.00 | -1.87 | 0.00 |
| 118 | 670 | CGA | 1 | 0 | 1.16 | 1.48 | 0.86 | 0.00 | -0.86 | 0.00 |
| 119 | 673 | UCU | 0 | 1 | 1.00 | 2.00 | 0.00 | 0.50 | 0.50 | 0.33 |
| 120 | 676 | CUG | 1 | 1 | 1.13 | 1.86 | 0.88 | 0.54 | -0.35 | 0.14 |
| 121 | 679 | AAA | 1 | 0 | 0.61 | 2.34 | 1.63 | 0.00 | -1.63 | 0.00 |
| 122 | 682 | UCG | 1 | 0 | 1.00 | 1.87 | 1.00 | 0.00 | -1.00 | 0.00 |
| 123 | 685 | CCC | 0 | 0 | 1.00 | 2.00 | 0.00 | 0.00 | 0.00 | N/A |
| 124 | 688 | AUU | 0 | 0 | 0.68 | 2.32 | 0.00 | 0.00 | 0.00 | N/A |
| 125 | 691 | CAG | 0 | 0 | 0.53 | 2.01 | 0.00 | 0.00 | 0.00 | N/A |
| 126 | 694 | UCC | 1 | 1 | 0.88 | 2.08 | 1.13 | 0.48 | -0.65 | 0.09 |
| 127 | 697 | GAC | 1 | 0 | 0.46 | 2.54 | 2.17 | 0.00 | -2.17 | 0.00 |
| 128 | 700 | UUG | 1 | 0 | 1.22 | 1.73 | 0.82 | 0.00 | -0.82 | 0.00 |
| 129 | 703 | GAU | 0 | 0 | 0.62 | 2.38 | 0.00 | 0.00 | 0.00 | N/A |
| 130 | 706 | GCG | 1 | 0 | 1.00 | 2.00 | 1.00 | 0.00 | -1.00 | 0.00 |
| 131 | 709 | UUC | 0 | 0 | 0.46 | 2.54 | 0.00 | 0.00 | 0.00 | N/A |
| 132 | 712 | CAC | 2 | 0 | 0.50 | 2.50 | 3.99 | 0.00 | -3.99 | 0.00 |
| 133 | 715 | UCG | 1 | 0 | 1.00 | 1.84 | 1.00 | 0.00 | -1.00 | 0.00 |
| 134 | 718 | GGA | 0 | 0 | 1.00 | 1.82 | 0.00 | 0.00 | 0.00 | N/A |
| 135 | 721 | UUU | 0 | 0 | 0.62 | 2.38 | 0.00 | 0.00 | 0.00 | N/A |
| 136 | 724 | CAA | 0 | 0 | 0.64 | 1.90 | 0.00 | 0.00 | 0.00 | N/A |
| 137 | 727 | ACA | 1 | 0 | 1.00 | 2.00 | 1.00 | 0.00 | -1.00 | 0.00 |
| 138 | 730 | UGC | 0 | 0 | 0.46 | 2.38 | 0.00 | 0.00 | 0.00 | N/A |
| 139 | 733 | GCC | 0 | 0 | 1.00 | 2.00 | 0.00 | 0.00 | 0.00 | N/A |
| 140 | 736 | AAA | 0 | 0 | 0.64 | 2.32 | 0.00 | 0.00 | 0.00 | N/A |
| 141 | 739 | GAA | 0 | 0 | 0.64 | 2.18 | 0.00 | 0.00 | 0.00 | N/A |
| 142 | 742 | GUC | 0 | 0 | 1.00 | 2.00 | 0.00 | 0.00 | 0.00 | N/A |
| 143 | 745 | UUG | 0 | 0 | 1.15 | 1.79 | 0.00 | 0.00 | 0.00 | N/A |
| 144 | 748 | CAA | 1 | 0 | 0.61 | 1.93 | 1.63 | 0.00 | -1.63 | 0.00 |
| 145 | 751 | UAC | 0 | 0 | 0.46 | 2.00 | 0.00 | 0.00 | 0.00 | N/A |
| 146 | 754 | CUC | 0 | 0 | 1.00 | 2.00 | 0.00 | 0.00 | 0.00 | N/A |
| 147 | 757 | UCC | 0 | 1 | 1.00 | 2.00 | 0.00 | 0.50 | 0.50 | 0.33 |
| 148 | 760 | CGG | 0 | 0 | 1.16 | 1.84 | 0.00 | 0.00 | 0.00 | N/A |
| 149 | 763 | UUU | 0 | 0 | 0.62 | 2.38 | 0.00 | 0.00 | 0.00 | N/A |
| 150 | 766 | GAG | 1 | 0 | 0.56 | 2.26 | 1.80 | 0.00 | -1.80 | 0.00 |
| 151 | 769 | AGC | 1 | 0 | 0.49 | 2.51 | 2.02 | 0.00 | -2.02 | 0.00 |
| 152 | 772 | UGG | 0 | 0 | 0.00 | 1.93 | 0.00 | 0.00 | 0.00 | N/A |
| 153 | 775 | ACA | 2 | 0 | 1.00 | 2.00 | 2.00 | 0.00 | -2.00 | 0.00 |
| 154 | 778 | CCC | 1 | 0 | 1.00 | 2.00 | 1.00 | 0.00 | -1.00 | 0.00 |
| 155 | 781 | AGG | 1 | 0 | 0.85 | 2.15 | 1.17 | 0.00 | -1.17 | 0.00 |
| 156 | 784 | GAG | 1 | 0 | 0.54 | 2.28 | 1.86 | 0.00 | -1.86 | 0.00 |
| 157 | 787 | CCG | 1 | 2 | 0.88 | 2.00 | 1.14 | 1.00 | -0.14 | 0.22 |
| 158 | 790 | CGG | 2 | 0 | 1.11 | 1.85 | 1.80 | 0.00 | -1.80 | 0.00 |
| 159 | 793 | UGU | 3 | 0 | 0.58 | 2.33 | 5.19 | 0.00 | -5.19 | 0.00 |
| 160 | 796 | GUC | 0 | 1 | 1.00 | 2.00 | 0.00 | 0.50 | 0.50 | 0.33 |
| 161 | 799 | CAG | 0 | 0 | 0.53 | 2.01 | 0.00 | 0.00 | 0.00 | N/A |
| 162 | 802 | CUG | 2 | 0 | 1.36 | 1.64 | 1.47 | 0.00 | -1.47 | 0.00 |
| 163 | 805 | AUC | 1 | 2 | 0.66 | 2.34 | 0.76 | 0.64 | -0.12 | 0.22 |
| 164 | 808 | AAC | 0 | 0 | 0.46 | 2.54 | 0.00 | 0.00 | 0.00 | N/A |
| 165 | 811 | CAC | 1 | 0 | 0.49 | 2.51 | 2.02 | 0.00 | -2.02 | 0.00 |
| 166 | 814 | UUG | 1 | 0 | 1.22 | 1.73 | 0.82 | 0.00 | -0.82 | 0.00 |
| 167 | 817 | CAC | 1 | 0 | 0.47 | 2.53 | 2.15 | 0.00 | -2.15 | 0.00 |
| 168 | 820 | GCC | 2 | 0 | 1.00 | 2.00 | 2.00 | 0.00 | -2.00 | 0.00 |
| 169 | 823 | GUG | 1 | 0 | 1.00 | 2.00 | 1.00 | 0.00 | -1.00 | 0.00 |
| 170 | 826 | GCC | 0 | 1 | 1.00 | 2.00 | 0.00 | 0.50 | 0.50 | 0.33 |
| 171 | 829 | ACC | 1 | 0 | 1.00 | 2.00 | 1.00 | 0.00 | -1.00 | 0.00 |
| 172 | 832 | CAG | 0 | 0 | 0.53 | 2.01 | 0.00 | 0.00 | 0.00 | N/A |
| 173 | 835 | UUC | 1 | 1 | 0.47 | 2.53 | 2.13 | 0.40 | -1.73 | 0.02 |
| 174 | 838 | UUG | 2 | 0 | 1.17 | 1.68 | 1.70 | 0.00 | -1.70 | 0.00 |
| 175 | 841 | CCC | 0 | 0 | 1.00 | 2.00 | 0.00 | 0.00 | 0.00 | N/A |
| 176 | 844 | ACC | 0 | 1 | 0.88 | 2.12 | 0.00 | 0.47 | 0.47 | 0.29 |
| 177 | 847 | CCG | 7 | 0 | 1.00 | 2.00 | 7.00 | 0.00 | -7.00 | 0.00 |
| 178 | 850 | CAG | 0 | 0 | 0.53 | 2.01 | 0.00 | 0.00 | 0.00 | N/A |
| 179 | 853 | CUG | 1 | 0 | 1.45 | 1.55 | 0.69 | 0.00 | -0.69 | 0.00 |
| 180 | 856 | UUG | 0 | 0 | 1.15 | 1.79 | 0.00 | 0.00 | 0.00 | N/A |
| 181 | 859 | ACU | 0 | 0 | 1.00 | 2.00 | 0.00 | 0.00 | 0.00 | N/A |
| 182 | 862 | CAA | 0 | 1 | 0.71 | 1.93 | 0.00 | 0.52 | 0.52 | 0.27 |
| 183 | 865 | CAG | 0 | 0 | 0.53 | 2.01 | 0.00 | 0.00 | 0.00 | N/A |
| 184 | 868 | GUC | 2 | 0 | 1.00 | 2.00 | 2.00 | 0.00 | -2.00 | 0.00 |
| 185 | 871 | CCU | 1 | 0 | 1.00 | 2.00 | 1.00 | 0.00 | -1.00 | 0.00 |
| 186 | 874 | CUG | 1 | 2 | 1.32 | 1.68 | 0.76 | 1.19 | 0.43 | 0.41 |
| 187 | 877 | AGC | 2 | 0 | 0.46 | 2.54 | 4.33 | 0.00 | -4.33 | 0.00 |
| 188 | 880 | AAA | 0 | 0 | 0.64 | 2.32 | 0.00 | 0.00 | 0.00 | N/A |
| 189 | 883 | GGC | 1 | 0 | 1.00 | 1.96 | 1.00 | 0.00 | -1.00 | 0.00 |
| 190 | 886 | ACC | 4 | 2 | 1.00 | 2.00 | 4.00 | 1.00 | -3.00 | 0.02 |
| 191 | 889 | GGC | 0 | 3 | 0.96 | 2.03 | 0.00 | 1.48 | 1.48 | 0.69 |
| 192 | 892 | GCU | 3 | 2 | 1.00 | 2.00 | 3.00 | 1.00 | -2.00 | 0.05 |
| 193 | 895 | CCC | 0 | 2 | 1.00 | 2.00 | 0.00 | 1.00 | 1.00 | 0.56 |
| 194 | 898 | UCG | 3 | 2 | 1.00 | 1.89 | 3.00 | 1.06 | -1.94 | 0.05 |
| 195 | 901 | GCC | 3 | 3 | 0.94 | 2.05 | 3.20 | 1.46 | -1.73 | 0.08 |
| 196 | 904 | GCC | 2 | 3 | 1.00 | 2.00 | 2.09 | 1.50 | -0.59 | 0.20 |
| 197 | 916 | GGG | 1 | 0 | 0.57 | 1.13 | 1.77 | 0.00 | -1.77 | 0.00 |
| 198 | 919 | UCC | 2 | 0 | 0.57 | 1.13 | 3.53 | 0.00 | -3.53 | 0.00 |
| 199 | 922 | GCG | 3 | 3 | 0.57 | 1.13 | 5.30 | 2.65 | -2.65 | 0.10 |
| 200 | 925 | GCC | 1 | 1 | 0.57 | 1.13 | 1.77 | 0.88 | -0.88 | 0.11 |
| 201 | 928 | GCC | 1 | 0 | 0.57 | 1.13 | 1.77 | 0.00 | -1.77 | 0.00 |
| 202 | 931 | CCC | 1 | 1 | 0.57 | 1.13 | 1.77 | 0.88 | -0.88 | 0.11 |
| 203 | 934 | UGC | 0 | 0 | 0.26 | 1.35 | 0.00 | 0.00 | 0.00 | N/A |
| 204 | 937 | CUG | 0 | 1 | 1.26 | 1.64 | 0.00 | 0.61 | 0.61 | 0.43 |
| 205 | 940 | GAG | 0 | 1 | 0.55 | 2.31 | 0.00 | 0.43 | 0.43 | 0.19 |
| 206 | 943 | CGC | 0 | 0 | 1.00 | 2.00 | 0.00 | 0.00 | 0.00 | N/A |
| 207 | 946 | GCG | 6 | 2 | 1.00 | 2.00 | 6.00 | 1.00 | -5.00 | 0.00 |
| 208 | 949 | GGG | 0 | 1 | 1.00 | 2.00 | 0.00 | 0.50 | 0.50 | 0.33 |
| 209 | 952 | CAG | 1 | 0 | 0.56 | 1.98 | 1.80 | 0.00 | -1.80 | 0.00 |
| 210 | 955 | AAG | 0 | 0 | 0.53 | 2.42 | 0.00 | 0.00 | 0.00 | N/A |
| 211 | 958 | CUG | 2 | 0 | 1.42 | 1.58 | 1.41 | 0.00 | -1.41 | 0.00 |
| 212 | 961 | GAG | 1 | 0 | 0.53 | 2.29 | 1.88 | 0.00 | -1.88 | 0.00 |
| 213 | 964 | CCC | 1 | 1 | 1.00 | 2.00 | 1.00 | 0.50 | -0.50 | 0.11 |
| 214 | 967 | CUC | 2 | 1 | 1.02 | 1.88 | 1.96 | 0.53 | -1.43 | 0.04 |
| 215 | 970 | GCC | 2 | 1 | 1.00 | 2.00 | 2.00 | 0.50 | -1.50 | 0.04 |
| 216 | 973 | UAC | 1 | 2 | 0.46 | 2.50 | 2.16 | 0.80 | -1.36 | 0.07 |
| 217 | 976 | UGC | 0 | 0 | 0.46 | 2.38 | 0.00 | 0.00 | 0.00 | N/A |
| 218 | 979 | GUG | 2 | 0 | 1.00 | 2.00 | 2.00 | 0.00 | -2.00 | 0.00 |
| 219 | 982 | CCC | 2 | 0 | 1.00 | 2.00 | 2.00 | 0.00 | -2.00 | 0.00 |
| 220 | 985 | GUC | 0 | 0 | 1.00 | 2.00 | 0.00 | 0.00 | 0.00 | N/A |
| 221 | 988 | AUC | 1 | 0 | 0.61 | 2.39 | 1.64 | 0.00 | -1.64 | 0.00 |
| 222 | 991 | CAG | 0 | 0 | 0.53 | 2.01 | 0.00 | 0.00 | 0.00 | N/A |
| 223 | 994 | CGG | 0 | 0 | 1.16 | 1.84 | 0.00 | 0.00 | 0.00 | N/A |
| 224 | 997 | ACU | 0 | 0 | 1.00 | 2.00 | 0.00 | 0.00 | 0.00 | N/A |
| 225 | 1000 | CAG | 0 | 1 | 0.52 | 2.12 | 0.00 | 0.47 | 0.47 | 0.20 |
| 226 | 1003 | CCC | 2 | 0 | 1.00 | 2.00 | 2.00 | 0.00 | -2.00 | 0.00 |
| 227 | 1006 | AGC | 1 | 3 | 0.64 | 2.36 | 1.31 | 1.34 | 0.03 | 0.27 |
| 228 | 1009 | GCC | 0 | 1 | 1.00 | 2.00 | 0.00 | 0.50 | 0.50 | 0.33 |
| 229 | 1012 | GAG | 0 | 0 | 0.53 | 2.29 | 0.00 | 0.00 | 0.00 | N/A |
| 230 | 1015 | CUC | 1 | 0 | 1.10 | 1.90 | 0.91 | 0.00 | -0.91 | 0.00 |
| 231 | 1018 | GCC | 0 | 2 | 0.92 | 2.08 | 0.00 | 0.96 | 0.96 | 0.52 |
| 232 | 1021 | GCC | 0 | 0 | 1.00 | 2.00 | 0.19 | 0.01 | -0.18 | 0.01 |
| 233 | 1027 | GAG | 1 | 0 | 0.54 | 2.28 | 1.86 | 0.00 | -1.86 | 0.00 |
| 234 | 1030 | AAC | 1 | 0 | 0.49 | 2.51 | 2.02 | 0.00 | -2.02 | 0.00 |
| 235 | 1033 | GAC | 1 | 0 | 0.46 | 2.54 | 2.15 | 0.00 | -2.15 | 0.00 |
| 236 | 1036 | ACG | 2 | 0 | 1.00 | 2.00 | 2.00 | 0.00 | -2.00 | 0.00 |
| 237 | 1039 | GAC | 0 | 0 | 0.46 | 2.54 | 0.00 | 0.00 | 0.00 | N/A |
| 238 | 1042 | ACC | 3 | 0 | 1.00 | 2.00 | 3.00 | 0.00 | -3.00 | 0.00 |
| 239 | 1045 | GAC | 1 | 0 | 0.47 | 2.53 | 2.15 | 0.00 | -2.15 | 0.00 |
| 240 | 1048 | AGC | 0 | 0 | 0.46 | 2.54 | 0.00 | 0.00 | 0.00 | N/A |
| 241 | 1051 | GGC | 0 | 0 | 1.00 | 2.00 | 0.00 | 0.00 | 0.00 | N/A |
| 242 | 1054 | UAC | 0 | 0 | 0.46 | 2.00 | 0.00 | 0.00 | 0.00 | N/A |
| 243 | 1057 | GGC | 2 | 0 | 1.00 | 1.96 | 2.00 | 0.00 | -2.00 | 0.00 |
| 244 | 1060 | GGC | 2 | 0 | 1.00 | 1.99 | 2.00 | 0.00 | -2.00 | 0.00 |
| 245 | 1063 | GAA | 2 | 0 | 0.55 | 2.27 | 3.65 | 0.00 | -3.65 | 0.00 |
| 246 | 1066 | GCC | 0 | 2 | 0.92 | 2.08 | 0.00 | 0.96 | 0.96 | 0.52 |
| 247 | 1069 | GAG | 0 | 0 | 0.53 | 2.29 | 0.00 | 0.00 | 0.00 | N/A |
| 248 | 1072 | GCC | 3 | 1 | 1.00 | 2.00 | 3.00 | 0.50 | -2.50 | 0.01 |
| 249 | 1075 | CGG | 4 | 0 | 1.08 | 1.92 | 3.71 | 0.00 | -3.71 | 0.00 |
| 250 | 1078 | CCG | 2 | 0 | 1.00 | 2.00 | 2.00 | 0.00 | -2.00 | 0.00 |
| 251 | 1081 | GAC | 1 | 0 | 0.49 | 2.51 | 2.02 | 0.00 | -2.02 | 0.00 |
| 252 | 1084 | CGC | 1 | 0 | 1.03 | 1.87 | 0.97 | 0.00 | -0.97 | 0.00 |
| 253 | 1087 | GAG | 1 | 1 | 0.54 | 2.28 | 1.84 | 0.44 | -1.40 | 0.04 |
| 254 | 1090 | AAA | 2 | 0 | 0.54 | 2.41 | 3.68 | 0.00 | -3.68 | 0.00 |
| 255 | 1093 | GGC | 1 | 2 | 0.97 | 2.03 | 1.03 | 0.99 | -0.05 | 0.25 |
| 256 | 1096 | AAA | 0 | 1 | 0.64 | 2.23 | 0.00 | 0.45 | 0.45 | 0.22 |
| 257 | 1099 | GGC | 0 | 2 | 0.98 | 2.02 | 0.00 | 0.99 | 0.99 | 0.55 |
| 258 | 1102 | GCG | 2 | 1 | 1.00 | 2.00 | 2.00 | 0.50 | -1.50 | 0.04 |
| 259 | 1105 | GGG | 1 | 1 | 0.97 | 2.03 | 1.03 | 0.49 | -0.54 | 0.10 |
| 260 | 1108 | GCG | 1 | 5 | 1.07 | 1.93 | 0.94 | 2.59 | 1.65 | 0.69 |
| 261 | 1111 | AGC | 0 | 3 | 0.62 | 2.38 | 0.00 | 1.26 | 1.26 | 0.50 |
| 262 | 1114 | CGC | 0 | 1 | 0.88 | 2.12 | 0.00 | 0.47 | 0.47 | 0.29 |
| 263 | 1117 | GUC | 2 | 1 | 1.07 | 1.93 | 1.88 | 0.52 | -1.36 | 0.04 |
| 264 | 1120 | ACC | 2 | 0 | 1.00 | 2.00 | 2.00 | 0.00 | -2.00 | 0.00 |
| 265 | 1123 | AUC | 1 | 0 | 0.62 | 2.38 | 1.61 | 0.00 | -1.61 | 0.00 |
| 266 | 1126 | AAG | 2 | 0 | 0.56 | 2.40 | 3.58 | 0.00 | -3.58 | 0.00 |
| 267 | 1129 | CAG | 0 | 0 | 0.53 | 2.01 | 0.00 | 0.00 | 0.00 | N/A |
| 268 | 1132 | GAG | 0 | 0 | 0.53 | 2.29 | 0.00 | 0.00 | 0.00 | N/A |
| 269 | 1135 | CCU | 2 | 0 | 1.00 | 2.00 | 2.00 | 0.00 | -2.00 | 0.00 |
| 270 | 1138 | CCC | 0 | 1 | 1.00 | 2.00 | 0.00 | 0.50 | 0.50 | 0.33 |
| 271 | 1141 | GGG | 1 | 0 | 1.00 | 2.00 | 1.00 | 0.00 | -1.00 | 0.00 |
| 272 | 1144 | GAG | 0 | 2 | 0.52 | 2.34 | 0.00 | 0.85 | 0.85 | 0.33 |
| 273 | 1147 | GAC | 0 | 1 | 0.48 | 2.49 | 0.00 | 0.40 | 0.40 | 0.16 |
| 274 | 1150 | UCG | 1 | 3 | 1.01 | 1.88 | 0.99 | 1.59 | 0.60 | 0.43 |
| 275 | 1153 | CCG | 2 | 0 | 1.00 | 2.00 | 2.00 | 0.00 | -2.00 | 0.00 |
| 276 | 1156 | GCG | 0 | 0 | 1.00 | 2.00 | 0.00 | 0.00 | 0.00 | N/A |
| 277 | 1159 | CCC | 1 | 0 | 1.00 | 2.00 | 1.00 | 0.00 | -1.00 | 0.00 |
| 278 | 1162 | AAG | 1 | 0 | 0.56 | 2.40 | 1.80 | 0.00 | -1.80 | 0.00 |
| 279 | 1165 | AGG | 0 | 0 | 0.85 | 2.15 | 0.00 | 0.00 | 0.00 | N/A |
| 280 | 1168 | AUG | 0 | 1 | 0.32 | 2.68 | 0.00 | 0.37 | 0.37 | 0.11 |
| 281 | 1171 | AAG | 1 | 4 | 0.57 | 2.39 | 0.88 | 1.46 | 0.58 | 0.37 |
| 282 | 1174 | CUG | 0 | 0 | 1.46 | 1.54 | 0.00 | 0.00 | 0.00 | N/A |
| 283 | 1177 | GAU | 0 | 0 | 0.62 | 2.38 | 0.00 | 0.00 | 0.00 | N/A |
| 284 | 1180 | UCC | 1 | 3 | 0.88 | 2.08 | 1.13 | 1.44 | 0.31 | 0.34 |
| 285 | 1183 | CGC | 2 | 2 | 0.96 | 2.04 | 2.45 | 1.10 | -1.35 | 0.11 |
| 286 | 1198 | GGC | 2 | 2 | 0.87 | 2.12 | 2.29 | 0.82 | -1.46 | 0.06 |
| 287 | 1201 | GGC | 0 | 1 | 0.88 | 2.12 | 0.00 | 0.47 | 0.47 | 0.29 |
| 288 | 1204 | GGC | 2 | 2 | 0.87 | 2.13 | 2.29 | 0.94 | -1.35 | 0.08 |
| 289 | 1207 | CCG | 2 | 6 | 0.93 | 2.07 | 2.16 | 2.89 | 0.73 | 0.47 |
| 290 | 1210 | GGG | 1 | 4 | 0.92 | 2.05 | 0.55 | 1.71 | 1.16 | 0.57 |
| 291 | 1213 | GGC | 2 | 1 | 0.88 | 2.12 | 2.27 | 0.47 | -1.79 | 0.03 |
| 292 | 1216 | GGC | 0 | 1 | 1.00 | 2.00 | 0.00 | 0.50 | 0.50 | 0.33 |
| 293 | 1219 | GCG | 1 | 1 | 1.00 | 2.00 | 1.00 | 0.50 | -0.50 | 0.11 |
| 294 | 1222 | GCG | 1 | 2 | 1.02 | 1.98 | 0.98 | 1.01 | 0.02 | 0.27 |
| 295 | 1225 | GCG | 1 | 2 | 1.00 | 2.00 | 1.00 | 1.00 | 0.00 | 0.26 |
| 296 | 1228 | GCG | 2 | 1 | 1.00 | 1.99 | 2.00 | 0.50 | -1.50 | 0.04 |
| 297 | 1231 | GCA | 5 | 0 | 1.00 | 2.00 | 5.00 | 0.00 | -5.00 | 0.00 |
| 298 | 1234 | GCC | 1 | 0 | 1.00 | 2.00 | 1.00 | 0.00 | -1.00 | 0.00 |
| 299 | 1237 | GCG | 1 | 0 | 1.00 | 2.00 | 1.00 | 0.00 | -1.00 | 0.00 |
| 300 | 1240 | CUU | 2 | 0 | 1.00 | 2.00 | 2.00 | 0.00 | -2.00 | 0.00 |
| 301 | 1243 | CUG | 2 | 2 | 1.20 | 1.80 | 1.39 | 1.30 | -0.09 | 0.29 |
| 302 | 1246 | GGG | 0 | 2 | 1.01 | 1.99 | 0.00 | 1.01 | 1.01 | 0.56 |
| 303 | 1249 | CCC | 7 | 2 | 0.88 | 2.12 | 7.93 | 0.94 | -6.98 | 0.00 |
| 304 | 1252 | GAC | 0 | 0 | 0.26 | 1.44 | 0.00 | 0.00 | 0.00 | N/A |
| 305 | 1255 | CCU | 2 | 0 | 0.57 | 1.13 | 3.53 | 0.00 | -3.53 | 0.00 |
| 306 | 1258 | GCC | 2 | 2 | 1.00 | 2.00 | 2.00 | 1.00 | -1.00 | 0.11 |
| 307 | 1261 | GCC | 4 | 1 | 1.00 | 2.00 | 4.00 | 0.50 | -3.50 | 0.00 |
| 308 | 1264 | GCG | 2 | 1 | 1.00 | 2.00 | 2.00 | 0.50 | -1.50 | 0.04 |
| 309 | 1267 | GCC | 3 | 0 | 1.00 | 2.00 | 3.00 | 0.00 | -3.00 | 0.00 |
| 310 | 1270 | GCG | 2 | 0 | 1.00 | 2.00 | 2.00 | 0.00 | -2.00 | 0.00 |
| 311 | 1273 | CUG | 1 | 0 | 1.46 | 1.54 | 0.69 | 0.00 | -0.69 | 0.00 |
| 312 | 1276 | CUG | 1 | 0 | 1.46 | 1.54 | 0.68 | 0.00 | -0.68 | 0.00 |
| 313 | 1279 | AGA | 0 | 0 | 0.96 | 2.00 | 0.00 | 0.00 | 0.00 | N/A |
| 314 | 1282 | CCC | 0 | 0 | 1.00 | 2.00 | 0.00 | 0.00 | 0.00 | N/A |
| 315 | 1285 | GAC | 0 | 0 | 0.46 | 2.54 | 0.00 | 0.00 | 0.00 | N/A |
| 316 | 1288 | GCC | 2 | 0 | 1.00 | 2.00 | 2.00 | 0.00 | -2.00 | 0.00 |
| 317 | 1291 | GCC | 1 | 0 | 1.00 | 2.00 | 1.00 | 0.00 | -1.00 | 0.00 |
| 318 | 1294 | CUG | 0 | 0 | 1.46 | 1.54 | 0.00 | 0.00 | 0.00 | N/A |
| 319 | 1297 | CUC | 0 | 0 | 1.00 | 2.00 | 0.00 | 0.00 | 0.00 | N/A |
| 320 | 1300 | AGC | 1 | 0 | 0.47 | 2.53 | 2.15 | 0.00 | -2.15 | 0.00 |
| 321 | 1303 | UCG | 2 | 0 | 1.00 | 1.87 | 2.00 | 0.00 | -2.00 | 0.00 |
| 322 | 1306 | CUG | 2 | 0 | 1.36 | 1.64 | 1.47 | 0.00 | -1.47 | 0.00 |
| 323 | 1309 | GUG | 1 | 1 | 1.10 | 1.90 | 0.91 | 0.53 | -0.38 | 0.13 |
| 324 | 1312 | GCG | 2 | 0 | 1.00 | 2.00 | 2.00 | 0.00 | -2.00 | 0.00 |
| 325 | 1315 | UUC | 1 | 0 | 0.49 | 2.51 | 2.02 | 0.00 | -2.02 | 0.00 |
| 326 | 1318 | GGC | 1 | 0 | 1.00 | 1.96 | 1.00 | 0.00 | -1.00 | 0.00 |
| 327 | 1321 | GGA | 1 | 0 | 1.00 | 1.82 | 1.00 | 0.00 | -1.00 | 0.00 |
| 328 | 1324 | GGC | 0 | 0 | 1.00 | 2.00 | 0.00 | 0.00 | 0.00 | N/A |
| 329 | 1327 | GGA | 1 | 0 | 1.00 | 2.00 | 1.00 | 0.00 | -1.00 | 0.00 |
| 330 | 1330 | GGC | 1 | 1 | 1.00 | 2.00 | 1.00 | 0.50 | -0.50 | 0.11 |
| 331 | 1333 | GCG | 1 | 1 | 1.00 | 2.00 | 1.00 | 0.50 | -0.50 | 0.11 |
| 332 | 1336 | CCC | 1 | 1 | 1.00 | 2.00 | 1.00 | 0.50 | -0.50 | 0.11 |
| 333 | 1339 | UUC | 0 | 2 | 0.62 | 2.38 | 0.00 | 0.84 | 0.84 | 0.37 |
| 334 | 1342 | CCG | 2 | 3 | 0.96 | 2.03 | 1.73 | 1.64 | -0.09 | 0.30 |
| 335 | 1345 | CAG | 1 | 3 | 0.63 | 2.05 | 0.79 | 1.22 | 0.43 | 0.35 |
| 336 | 1348 | CCC | 5 | 2 | 0.89 | 2.04 | 5.04 | 0.74 | -4.30 | 0.01 |
| 337 | 1351 | GCG | 4 | 2 | 0.91 | 2.01 | 4.38 | 1.00 | -3.38 | 0.01 |
| 338 | 1354 | GCC | 2 | 0 | 1.00 | 2.00 | 2.00 | 0.00 | -2.00 | 0.00 |
| 339 | 1357 | GCC | 2 | 0 | 1.00 | 2.00 | 2.13 | 0.03 | -2.10 | 0.00 |
| 340 | 1372 | GCC | 0 | 0 | 0.99 | 1.99 | 0.00 | 0.00 | 0.00 | N/A |
| 341 | 1375 | CCC | 2 | 0 | 0.99 | 1.99 | 2.01 | 0.00 | -2.01 | 0.00 |
| 342 | 1378 | UUC | 1 | 1 | 0.59 | 2.40 | 1.71 | 0.42 | -1.29 | 0.04 |
| 343 | 1381 | UGC | 0 | 0 | 0.46 | 2.37 | 0.00 | 0.00 | 0.00 | N/A |
| 344 | 1384 | CUG | 0 | 0 | 1.45 | 1.53 | 0.00 | 0.00 | 0.00 | N/A |
| 345 | 1387 | CCC | 1 | 0 | 0.99 | 1.99 | 1.01 | 0.00 | -1.01 | 0.00 |
| 346 | 1390 | UUC | 0 | 0 | 0.46 | 2.53 | 0.00 | 0.00 | 0.00 | N/A |
| 347 | 1393 | UGC | 1 | 1 | 0.46 | 2.02 | 2.18 | 0.50 | -1.68 | 0.03 |
| 348 | 1396 | UUC | 0 | 0 | 0.46 | 2.53 | 0.00 | 0.00 | 0.00 | N/A |
| 349 | 1399 | CUC | 0 | 1 | 0.99 | 1.99 | 0.00 | 0.50 | 0.50 | 0.33 |
| 350 | 1402 | UCG | 1 | 0 | 0.99 | 1.86 | 1.01 | 0.00 | -1.01 | 0.00 |
| 351 | 1405 | CCU | 2 | 0 | 0.99 | 1.99 | 2.01 | 0.00 | -2.01 | 0.00 |
| 352 | 1408 | UCU | 5 | 0 | 0.99 | 1.88 | 5.03 | 0.00 | -5.03 | 0.00 |
| 353 | 1411 | GCA | 3 | 0 | 0.99 | 1.99 | 3.02 | 0.00 | -3.02 | 0.00 |
| 354 | 1414 | GCU | 1 | 0 | 0.99 | 1.99 | 1.01 | 0.00 | -1.01 | 0.00 |
| 355 | 1417 | GCC | 0 | 0 | 0.99 | 1.99 | 0.00 | 0.00 | 0.00 | N/A |
| 356 | 1420 | GCC | 0 | 0 | 0.99 | 1.99 | 0.00 | 0.00 | 0.00 | N/A |
| 357 | 1423 | UAC | 0 | 0 | 0.46 | 1.99 | 0.00 | 0.00 | 0.00 | N/A |
| 358 | 1426 | GUG | 0 | 1 | 0.78 | 2.21 | 0.00 | 0.45 | 0.45 | 0.26 |
| 359 | 1429 | CAG | 0 | 0 | 0.53 | 2.00 | 0.00 | 0.00 | 0.00 | N/A |
| 360 | 1432 | CCC | 2 | 0 | 0.99 | 1.99 | 2.01 | 0.00 | -2.01 | 0.00 |
| 361 | 1435 | UUC | 0 | 1 | 0.61 | 2.36 | 0.00 | 0.42 | 0.42 | 0.20 |
| 362 | 1438 | CUG | 0 | 0 | 1.45 | 1.53 | 0.00 | 0.00 | 0.00 | N/A |
| 363 | 1441 | GAC | 0 | 0 | 0.46 | 2.53 | 0.00 | 0.00 | 0.00 | N/A |
| 364 | 1444 | AAG | 0 | 0 | 0.53 | 2.41 | 0.00 | 0.00 | 0.00 | N/A |
| 365 | 1447 | AGC | 0 | 0 | 0.46 | 2.53 | 0.00 | 0.00 | 0.00 | N/A |
| 366 | 1450 | GGC | 0 | 4 | 0.82 | 2.17 | 0.00 | 1.85 | 1.85 | 0.72 |
| 367 | 1453 | CUG | 0 | 0 | 1.45 | 1.53 | 0.00 | 0.00 | 0.00 | N/A |
| 368 | 1456 | GAG | 0 | 0 | 0.53 | 2.27 | 0.00 | 0.00 | 0.00 | N/A |
| 369 | 1459 | AAG | 1 | 0 | 0.55 | 2.39 | 1.81 | 0.00 | -1.81 | 0.00 |
| 370 | 1462 | UAU | 2 | 0 | 0.56 | 1.99 | 3.55 | 0.00 | -3.55 | 0.00 |
| 371 | 1465 | CUG | 1 | 0 | 1.35 | 1.63 | 0.74 | 0.00 | -0.74 | 0.00 |
| 372 | 1468 | UAC | 1 | 0 | 0.46 | 1.99 | 2.18 | 0.00 | -2.18 | 0.00 |
| 373 | 1471 | CCG | 1 | 0 | 0.99 | 1.99 | 1.04 | 0.00 | -1.04 | 0.00 |
| 374 | 1474 | GCG | 0 | 1 | 0.56 | 1.12 | 0.00 | 0.89 | 0.89 | 0.33 |
| 375 | 1477 | GCG | 1 | 0 | 0.99 | 1.99 | 1.01 | 0.02 | -0.99 | 0.00 |
| 376 | 1480 | GCU | 3 | 0 | 0.99 | 1.99 | 3.02 | 0.00 | -3.02 | 0.00 |
| 377 | 1483 | GCC | 0 | 0 | 0.99 | 1.99 | 0.00 | 0.00 | 0.00 | N/A |
| 378 | 1486 | GCC | 0 | 1 | 0.99 | 1.99 | 0.00 | 0.50 | 0.50 | 0.33 |
| 379 | 1489 | CCG | 3 | 0 | 0.99 | 1.99 | 3.02 | 0.00 | -3.02 | 0.00 |
| 380 | 1492 | UUC | 1 | 1 | 0.49 | 2.49 | 2.03 | 0.40 | -1.62 | 0.03 |
| 381 | 1495 | CCG | 5 | 0 | 0.99 | 1.99 | 5.03 | 0.00 | -5.03 | 0.00 |
| 382 | 1498 | CUG | 4 | 0 | 1.37 | 1.59 | 2.91 | 0.00 | -2.91 | 0.00 |
| 383 | 1501 | CUA | 5 | 0 | 1.33 | 1.65 | 3.76 | 0.00 | -3.76 | 0.00 |
| 384 | 1504 | UAC | 0 | 0 | 0.46 | 1.99 | 0.00 | 0.00 | 0.00 | N/A |
| 385 | 1507 | CCC | 2 | 0 | 0.99 | 1.99 | 2.01 | 0.00 | -2.01 | 0.00 |
| 386 | 1510 | GGC | 2 | 0 | 0.99 | 1.94 | 2.01 | 0.00 | -2.01 | 0.00 |
| 387 | 1513 | AUC | 0 | 0 | 0.62 | 2.37 | 0.00 | 0.00 | 0.00 | N/A |
| 388 | 1516 | CCC | 2 | 0 | 0.99 | 1.99 | 2.01 | 0.00 | -2.01 | 0.00 |
| 389 | 1519 | GCC | 3 | 0 | 0.99 | 1.99 | 3.02 | 0.00 | -3.02 | 0.00 |
| 390 | 1522 | CCG | 3 | 1 | 0.89 | 1.99 | 3.36 | 0.50 | -2.86 | 0.01 |
| 391 | 1525 | GCG | 2 | 0 | 0.56 | 1.12 | 3.89 | 0.02 | -3.87 | 0.00 |
| 392 | 1531 | GCC | 1 | 0 | 0.56 | 1.12 | 1.78 | 0.01 | -1.77 | 0.00 |
| 393 | 1534 | GCG | 2 | 0 | 0.56 | 1.12 | 3.57 | 0.00 | -3.57 | 0.00 |
| 394 | 1537 | GCA | 1 | 0 | 0.56 | 1.12 | 1.78 | 0.00 | -1.78 | 0.00 |
| 395 | 1540 | GCC | 5 | 0 | 0.56 | 1.12 | 8.92 | 0.00 | -8.92 | 0.00 |
| 396 | 1543 | GCC | 3 | 0 | 0.56 | 1.12 | 5.35 | 0.00 | -5.35 | 0.00 |
| 397 | 1546 | GCC | 5 | 0 | 0.56 | 1.12 | 8.92 | 0.00 | -8.92 | 0.00 |
| 398 | 1549 | GCC | 7 | 0 | 0.56 | 1.12 | 12.49 | 0.00 | -12.49 | 0.00 |
| 399 | 1552 | GCU | 3 | 0 | 0.56 | 1.12 | 5.35 | 0.00 | -5.35 | 0.00 |
| 400 | 1555 | GCC | 5 | 0 | 0.57 | 1.13 | 8.83 | 0.00 | -8.83 | 0.00 |
| 401 | 1558 | GCC | 1 | 0 | 0.57 | 1.13 | 1.77 | 0.00 | -1.77 | 0.00 |
| 402 | 1561 | GCC | 3 | 0 | 1.00 | 2.00 | 3.00 | 0.00 | -3.00 | 0.00 |
| 403 | 1564 | GCC | 1 | 0 | 1.00 | 2.00 | 1.00 | 0.00 | -1.00 | 0.00 |
| 404 | 1567 | GCC | 2 | 0 | 1.00 | 2.00 | 2.00 | 0.00 | -2.00 | 0.00 |
| 405 | 1570 | GCG | 1 | 0 | 1.00 | 2.00 | 1.00 | 0.00 | -1.00 | 0.00 |
| 406 | 1573 | UUC | 1 | 0 | 0.47 | 2.53 | 2.15 | 0.00 | -2.15 | 0.00 |
| 407 | 1576 | CCC | 0 | 0 | 1.00 | 2.00 | 0.00 | 0.00 | 0.00 | N/A |
| 408 | 1579 | UGC | 2 | 0 | 0.47 | 2.38 | 4.29 | 0.00 | -4.29 | 0.00 |
| 409 | 1582 | CUG | 1 | 0 | 1.36 | 1.64 | 0.74 | 0.00 | -0.74 | 0.00 |
| 410 | 1585 | UCC | 0 | 0 | 1.00 | 2.00 | 0.00 | 0.00 | 0.00 | N/A |
| 411 | 1588 | UCG | 4 | 0 | 1.00 | 1.85 | 4.00 | 0.00 | -4.00 | 0.00 |
| 412 | 1591 | GUG | 0 | 0 | 1.00 | 2.00 | 0.00 | 0.00 | 0.00 | N/A |
| 413 | 1594 | UUG | 1 | 0 | 1.22 | 1.73 | 0.82 | 0.00 | -0.82 | 0.00 |
| 414 | 1597 | UCG | 0 | 1 | 1.00 | 1.87 | 0.00 | 0.53 | 0.53 | 0.35 |
| 415 | 1600 | CCC | 1 | 1 | 1.00 | 1.92 | 1.00 | 0.52 | -0.48 | 0.12 |
| 416 | 1603 | CCU | 1 | 1 | 1.00 | 2.00 | 1.00 | 0.50 | -0.50 | 0.11 |
| 417 | 1606 | CCC | 2 | 0 | 0.57 | 1.13 | 3.53 | 0.00 | -3.53 | 0.00 |
| 418 | 1609 | GAG | 1 | 0 | 0.54 | 2.28 | 1.87 | 0.00 | -1.87 | 0.00 |
| 419 | 1612 | AAG | 1 | 0 | 0.53 | 2.42 | 1.87 | 0.00 | -1.87 | 0.00 |
| 420 | 1615 | GCG | 1 | 1 | 1.00 | 2.00 | 1.30 | 0.50 | -0.80 | 0.11 |
| 421 | 1624 | GGC | 3 | 5 | 0.99 | 1.99 | 3.02 | 2.51 | -0.51 | 0.26 |
| 422 | 1627 | GCC | 2 | 0 | 1.00 | 2.00 | 2.00 | 0.00 | -2.00 | 0.00 |
| 423 | 1630 | GCC | 3 | 0 | 1.00 | 2.00 | 3.00 | 0.00 | -3.00 | 0.00 |
| 424 | 1633 | GCC | 0 | 0 | 1.00 | 2.00 | 0.00 | 0.00 | 0.00 | N/A |
| 425 | 1636 | GCG | 0 | 1 | 1.00 | 1.97 | 0.00 | 0.51 | 0.51 | 0.34 |
| 426 | 1639 | ACC | 0 | 1 | 1.00 | 2.00 | 0.00 | 0.50 | 0.50 | 0.33 |
| 427 | 1642 | CUC | 0 | 0 | 1.00 | 2.00 | 0.00 | 0.00 | 0.00 | N/A |
| 428 | 1645 | CUG | 3 | 0 | 1.45 | 1.55 | 2.08 | 0.00 | -2.08 | 0.00 |
| 429 | 1648 | CCG | 2 | 1 | 1.00 | 1.99 | 2.00 | 0.50 | -1.50 | 0.04 |
| 430 | 1651 | CAC | 0 | 1 | 0.58 | 2.42 | 0.00 | 0.41 | 0.41 | 0.19 |
| 431 | 1654 | GAG | 0 | 1 | 0.52 | 2.34 | 0.00 | 0.43 | 0.43 | 0.18 |
| 432 | 1657 | GUG | 0 | 0 | 1.00 | 2.00 | 0.00 | 0.00 | 0.00 | N/A |
| 433 | 1660 | GCG | 1 | 1 | 1.00 | 2.00 | 1.00 | 0.50 | -0.50 | 0.11 |
| 434 | 1663 | CCC | 1 | 2 | 1.00 | 1.98 | 1.00 | 1.01 | 0.01 | 0.26 |
| 435 | 1666 | CUU | 1 | 1 | 1.00 | 2.00 | 1.00 | 0.50 | -0.50 | 0.11 |
| 436 | 1669 | GGG | 1 | 1 | 0.57 | 1.13 | 1.77 | 0.89 | -0.88 | 0.11 |
| 437 | 1672 | GCG | 3 | 1 | 0.57 | 1.12 | 5.30 | 0.90 | -4.40 | 0.01 |
| 438 | 1675 | CCG | 1 | 1 | 0.76 | 0.93 | 1.31 | 1.07 | -0.24 | 0.20 |
| 439 | 1678 | CAC | 2 | 1 | 0.27 | 1.43 | 7.35 | 0.70 | -6.65 | 0.00 |
| 440 | 1681 | CCC | 1 | 2 | 1.00 | 2.00 | 1.00 | 1.00 | 0.00 | 0.26 |
| 441 | 1684 | CAG | 2 | 3 | 0.97 | 2.00 | 2.07 | 1.50 | -0.57 | 0.20 |
| 442 | 1687 | CAC | 2 | 1 | 0.50 | 2.50 | 4.04 | 0.40 | -3.64 | 0.00 |
| 443 | 1690 | CCG | 0 | 1 | 1.10 | 1.90 | 0.00 | 0.53 | 0.53 | 0.37 |
| 444 | 1693 | CAC | 0 | 1 | 0.58 | 2.42 | 0.00 | 0.41 | 0.41 | 0.19 |
| 445 | 1696 | GGC | 2 | 2 | 0.91 | 2.09 | 2.21 | 0.95 | -1.25 | 0.09 |
| 446 | 1699 | CGC | 0 | 1 | 1.00 | 2.00 | 0.00 | 0.50 | 0.50 | 0.33 |
| 447 | 1702 | ACC | 0 | 2 | 0.89 | 2.11 | 0.00 | 0.95 | 0.95 | 0.51 |
| 448 | 1705 | CAC | 0 | 2 | 0.58 | 2.42 | 0.00 | 0.83 | 0.83 | 0.35 |
| 449 | 1708 | CUG | 1 | 2 | 1.31 | 1.69 | 0.76 | 1.18 | 0.42 | 0.41 |
| 450 | 1711 | CCC | 1 | 2 | 1.00 | 2.00 | 1.00 | 1.00 | 0.00 | 0.26 |
| 451 | 1714 | UUC | 1 | 1 | 0.47 | 2.50 | 2.15 | 0.40 | -1.75 | 0.02 |
| 452 | 1717 | GCC | 2 | 2 | 0.89 | 2.08 | 1.68 | 0.72 | -0.96 | 0.12 |
| 453 | 1720 | GGG | 2 | 3 | 0.98 | 2.01 | 1.87 | 1.57 | -0.30 | 0.26 |
| 454 | 1723 | CCC | 2 | 5 | 1.00 | 2.00 | 2.00 | 2.50 | 0.50 | 0.43 |
| 455 | 1726 | CGC | 2 | 3 | 0.94 | 2.06 | 2.14 | 1.45 | -0.68 | 0.18 |
| 456 | 1729 | GAG | 0 | 3 | 0.57 | 2.31 | 0.29 | 1.23 | 0.93 | 0.42 |
| 457 | 1732 | CCG | 2 | 4 | 1.01 | 1.99 | 1.97 | 2.01 | 0.04 | 0.33 |
| 458 | 1735 | GGG | 1 | 0 | 1.00 | 1.99 | 1.00 | 0.00 | -1.00 | 0.00 |
| 459 | 1738 | AAC | 0 | 1 | 0.46 | 2.54 | 0.00 | 0.39 | 0.39 | 0.15 |
| 460 | 1741 | CCG | 2 | 3 | 0.96 | 2.04 | 1.91 | 1.55 | -0.36 | 0.24 |
| 461 | 1744 | GAG | 0 | 1 | 0.52 | 2.34 | 0.00 | 0.43 | 0.43 | 0.18 |
| 462 | 1747 | AGC | 1 | 2 | 0.60 | 2.40 | 1.68 | 0.83 | -0.84 | 0.10 |
| 463 | 1750 | UCU | 4 | 2 | 0.97 | 2.03 | 4.14 | 0.99 | -3.15 | 0.02 |
| 464 | 1753 | GCU | 1 | 2 | 1.00 | 2.00 | 1.00 | 1.00 | 0.00 | 0.26 |
| 465 | 1756 | CAG | 0 | 2 | 0.64 | 2.00 | 0.00 | 1.00 | 1.00 | 0.43 |
| 466 | 1759 | GAA | 0 | 0 | 0.64 | 2.18 | 0.00 | 0.00 | 0.00 | N/A |
| 467 | 1762 | GAU | 1 | 1 | 0.63 | 2.37 | 1.60 | 0.42 | -1.17 | 0.04 |
| 468 | 1765 | CCC | 2 | 3 | 0.98 | 2.02 | 2.03 | 1.49 | -0.55 | 0.20 |
| 469 | 1768 | UCG | 1 | 1 | 1.03 | 1.81 | 0.97 | 0.55 | -0.42 | 0.13 |
| 470 | 1771 | CAG | 0 | 0 | 0.53 | 2.01 | 0.00 | 0.00 | 0.00 | N/A |
| 471 | 1774 | CCA | 2 | 1 | 1.00 | 1.96 | 2.00 | 0.51 | -1.49 | 0.04 |
| 472 | 1777 | GGA | 1 | 1 | 1.00 | 1.83 | 1.00 | 0.55 | -0.45 | 0.12 |
| 473 | 1780 | AAG | 0 | 0 | 0.53 | 2.42 | 0.00 | 0.00 | 0.00 | N/A |
| 474 | 1783 | GAA | 1 | 0 | 0.63 | 2.19 | 1.58 | 0.00 | -1.58 | 0.00 |
| 475 | 1786 | GCU | 3 | 4 | 0.87 | 2.13 | 3.46 | 1.87 | -1.59 | 0.11 |
| 476 | 1789 | CCC | 0 | 2 | 0.99 | 2.01 | 0.00 | 1.00 | 1.00 | 0.55 |
