# Supplementary material for: Unexpected predicted length variation for the coding sequence of the sleep related gene, BHLHE41 in gorilla amidst strong purifying selection across mammals
Source: PLoS One. 2020 Apr 14;15(4):e0223203. doi: 10.1371/journal.pone.0223203 (PMC7156063; doi:10.1371/journal.pone.0223203)
Supplement: S3 Table — (DOCX) [file pone.0223203.s006.docx]

S3 Table. *BHLHE41* mammalian nucleotide alignment with reptile outgroup.

NM_030762 ------------------------------------------------------------

XM_520805 ------------------------------------------------------------

XM_019037881 AUGGUACGUUCCGCACGUGAGCUGGGUGCUGGUCUGGCCGGCGACGCGCGUGCCCUGUGG

XM_002823045 ------------------------------------------------------------

XM_005570417 ------------------------------------------------------------

XM_012093655 ------------------------------------------------------------

XM_015151321 ------------------------------------------------------------

XM_011759130 ------------------------------------------------------------

XM_007967990 ------------------------------------------------------------

XM_023209042 ------------------------------------------------------------

XM_025402281 ------------------------------------------------------------

XM_017507035 ------------------------------------------------------------

XM_012739537 ------------------------------------------------------------

XM_007446307 ------------------------------------------------------------

XM_024128992 ------------------------------------------------------------

XM_027129408 ------------------------------------------------------------

XM_025879601 ------------------------------------------------------------

XM_022577811 ------------------------------------------------------------

XM_019936346 ------------------------------------------------------------

XM_027593397 ------------------------------------------------------------

XM_015093964 ------------------------------------------------------------

XM_019452268 ------------------------------------------------------------

XM_004270956 ------------------------------------------------------------

XM_027541573 ------------------------------------------------------------

XM_027934162 ------------------------------------------------------------

XM_003355541 ------------------------------------------------------------

XM_016119294 ------------------------------------------------------------

XM_006127674 ------------------------------------------------------------

NM_030762 ------------------------------------------------------------

XM_520805 ------------------------------------------------------------

XM_019037881 CCAAACACUGCCUGGAGUGAGAGCAAACUACCAGCGCAGUGGGGCCGGCGCGAGUGUGCG

XM_002823045 ------------------------------------------------------------

XM_005570417 ------------------------------------------------------------

XM_012093655 ------------------------------------------------------------

XM_015151321 ------------------------------------------------------------

XM_011759130 ------------------------------------------------------------

XM_007967990 ------------------------------------------------------------

XM_023209042 ------------------------------------------------------------

XM_025402281 ------------------------------------------------------------

XM_017507035 ------------------------------------------------------------

XM_012739537 ------------------------------------------------------------

XM_007446307 ------------------------------------------------------------

XM_024128992 ------------------------------------------------------------

XM_027129408 ------------------------------------------------------------

XM_025879601 ------------------------------------------------------------

XM_022577811 ------------------------------------------------------------

XM_019936346 ------------------------------------------------------------

XM_027593397 ------------------------------------------------------------

XM_015093964 ------------------------------------------------------------

XM_019452268 ------------------------------------------------------------

XM_004270956 ------------------------------------------------------------

XM_027541573 ------------------------------------------------------------

XM_027934162 ------------------------------------------------------------

XM_003355541 ------------------------------------------------------------

XM_016119294 ------------------------------------------------------------

XM_006127674 ------------------------------------------------------------

NM_030762 ------------------------------------------------------------

XM_520805 ------------------------------------------------------------

XM_019037881 UGUGUGUGCGUGUGUGUGUGCGAGCGCGGUGGAGGGGGAGACCAACUGCUUCACACUUUC

XM_002823045 ------------------------------------------------------------

XM_005570417 ------------------------------------------------------------

XM_012093655 ------------------------------------------------------------

XM_015151321 ------------------------------------------------------------

XM_011759130 ------------------------------------------------------------

XM_007967990 ------------------------------------------------------------

XM_023209042 ------------------------------------------------------------

XM_025402281 ------------------------------------------------------------

XM_017507035 ------------------------------------------------------------

XM_012739537 ------------------------------------------------------------

XM_007446307 ------------------------------------------------------------

XM_024128992 ------------------------------------------------------------

XM_027129408 ------------------------------------------------------------

XM_025879601 ------------------------------------------------------------

XM_022577811 ------------------------------------------------------------

XM_019936346 ------------------------------------------------------------

XM_027593397 ------------------------------------------------------------

XM_015093964 ------------------------------------------------------------

XM_019452268 ------------------------------------------------------------

XM_004270956 ------------------------------------------------------------

XM_027541573 ------------------------------------------------------------

XM_027934162 ------------------------------------------------------------

XM_003355541 ------------------------------------------------------------

XM_016119294 ------------------------------------------------------------

XM_006127674 ------------------------------------------------------------

NM_030762 ------------------------------------------------------------

XM_520805 ------------------------------------------------------------

XM_019037881 AACACUGCACUGAAGAGGGAGAGCGAGAGAGAGACUGGAGACGCACAGAUCCCCCCAAGG

XM_002823045 ------------------------------------------------------------

XM_005570417 ------------------------------------------------------------

XM_012093655 ------------------------------------------------------------

XM_015151321 ------------------------------------------------------------

XM_011759130 ------------------------------------------------------------

XM_007967990 ------------------------------------------------------------

XM_023209042 ------------------------------------------------------------

XM_025402281 ------------------------------------------------------------

XM_017507035 ------------------------------------------------------------

XM_012739537 ------------------------------------------------------------

XM_007446307 ------------------------------------------------------------

XM_024128992 ------------------------------------------------------------

XM_027129408 ------------------------------------------------------------

XM_025879601 ------------------------------------------------------------

XM_022577811 ------------------------------------------------------------

XM_019936346 ------------------------------------------------------------

XM_027593397 ------------------------------------------------------------

XM_015093964 ------------------------------------------------------------

XM_019452268 ------------------------------------------------------------

XM_004270956 ------------------------------------------------------------

XM_027541573 ------------------------------------------------------------

XM_027934162 ------------------------------------------------------------

XM_003355541 ------------------------------------------------------------

XM_016119294 ------------------------------------------------------------

XM_006127674 ------------------------------------------------------------

NM_030762 ------------------------------------------------------------

XM_520805 ------------------------------------------------------------

XM_019037881 UCUCCCAAGCCUACCGUCCCACAGAUUAUUGUACAGAGCCCCAAAAAUCGAAACAGAGGA

XM_002823045 ------------------------------------------------------------

XM_005570417 ------------------------------------------------------------

XM_012093655 ------------------------------------------------------------

XM_015151321 ------------------------------------------------------------

XM_011759130 ------------------------------------------------------------

XM_007967990 ------------------------------------------------------------

XM_023209042 ------------------------------------------------------------

XM_025402281 ------------------------------------------------------------

XM_017507035 ------------------------------------------------------------

XM_012739537 ------------------------------------------------------------

XM_007446307 ------------------------------------------------------------

XM_024128992 ------------------------------------------------------------

XM_027129408 ------------------------------------------------------------

XM_025879601 ------------------------------------------------------------

XM_022577811 ------------------------------------------------------------

XM_019936346 ------------------------------------------------------------

XM_027593397 ------------------------------------------------------------

XM_015093964 ------------------------------------------------------------

XM_019452268 ------------------------------------------------------------

XM_004270956 ------------------------------------------------------------

XM_027541573 ------------------------------------------------------------

XM_027934162 ------------------------------------------------------------

XM_003355541 ------------------------------------------------------------

XM_016119294 ------------------------------------------------------------

XM_006127674 ------------------------------------------------------------

NM_030762 ------------------AUGGACGAAGGAAUUCCUCAUUUGCAAGAGAGACAGUUACUG

XM_520805 ------------------AUGGACGAAGGAAUUCCUCAUUUGCAAGAGAGACAGUUACUG

XM_019037881 AACGAACAGCAGUUGAACAUGGACGAAGGAAUUCCUCAUUUGCAAGAGAGACAAUUACUG

XM_002823045 ------------------AUGGACGAAGGAAUUCCUCAUUUGCAAGAGAGACAGUUACUG

XM_005570417 ------------------AUGGACGAAGGAAUUCCUCAUUUGCAAGAGAGACAGUUACUG

XM_012093655 ------------------AUGGACGAAGGAAUUCCUCAUUUGCAAGAGAGACAGUUACUG

XM_015151321 ------------------AUGGACGAAGGAAUUCCUCAUUUGCAAGAGAGACAGUUACUG

XM_011759130 ------------------AUGGACGAAGGAAUUCCUCAUUUGCAAGAGAGACAGUUACUG

XM_007967990 ------------------AUGGACGAAGGAAUUCCUCAUUUGCAAGAGAGACAGUUACUG

XM_023209042 ------------------AUGGACGAAGGAAUUCCUCAUUUGCAAGAGAGACAGUUACUG

XM_025402281 ------------------AUGGACGAAGGAAUUCCUCAUUUGCAAGAGAGACAGUUACUG

XM_017507035 ------------------AUGGACGAAGGAAUUCCUCAUUUGCAAGAGAGACAGUUACUG

XM_012739537 ------------------AUGGACGAAGGAAUUCCUCAUUUGCAAGAGAGACAGUUACUG

XM_007446307 ------------------AUGGACGAAGGAAUUCCUCAUUUGCAAGAGAGACAGUUACUG

XM_024128992 ------------------AUGGACGAAGGAAUUCCUCAUUUGCAAGAGAGACAGUUACUG

XM_027129408 ------------------AUGGACGAAGGAAUUCCUCAUUUGCAAGAGAGACAGUUACUG

XM_025879601 ------------------AUGGACGAAGGAAUUCCUCAUUUGCAAGAGAGACAGUUACUG

XM_022577811 ------------------AUGGACGAAGGAAUUCCUCAUUUGCAAGAGAGACAGUUACUG

XM_019936346 ------------------AUGGACGAAGGAAUUCCUCAUUUGCAAGAGAGACAGUUACUG

XM_027593397 ------------------AUGGACGAAGGAAUUCCUCAUUUGCAAGAGAGACAGUUACUG

XM_015093964 ------------------AUGGACGAAGGAAUUCCUCAUUUGCAAGAGAGACAGUUACUG

XM_019452268 ------------------AUGGACGAAGGAAUUCCUCAUUUGCAAGAGAGACAGUUACUG

XM_004270956 ------------------AUGGACGAAGGAAUUCCUCAUUUGCAAGAGAGACAGUUACUG

XM_027541573 ------------------AUGGACGAAGGAAUUCCUCAUUUGCAAGAGAGACAGUUACUG

XM_027934162 ------------------AUGGACGAAGGAAUUCCUCAUUUGCAAGAGAGACAGUUACUG

XM_003355541 ------------------AUGGACGAAGGAAUUCCUCAUUUGCAAGAAAGACAGUUACUG

XM_016119294 ------------------AUGGACGAAGGAAUUCCUCAUUUGCAAGAGAGACAGUUACUG

XM_006127674 ------------------AUGGAUGAAGGAAUCCCUCGUUUGCCAGACAGGCAGUUGCUA

NM_030762 GAACAUAGAGAUUUUAUAGGACUGGACUAUUCCUCUUUGUAUAUGUGUAAACCCAAAAGG

XM_520805 GAACAUAGAGAUUUUAUAGGACUGGACUAUUCCUCUUUGUAUAUGUGUAAACCCAAAAGG

XM_019037881 GAACAUAGAGAUUUUAUAGGACUGGACUAUUCCUCUUUGUAUAUGUGUAAACCCAAAAGG

XM_002823045 GAACAUAGAGAUUUUAUAGGACUGGACUAUUCCUCUUUGUAUAUGUGUAAACCCAAAAGG

XM_005570417 GAACAUAGAGAUUUUAUAGGACUGGAUUAUUCCUCUUUGUAUAUGUGUAAACCCAAAAGG

XM_012093655 GAACAUAGAGAUUUUAUAGGACUGGAUUAUUCCUCUUUGUAUAUGUGUAAACCCAAAAGG

XM_015151321 GAACAUAGAGAUUUUAUAGGACUGGAUUAUUCCUCUUUGUAUAUGUGUAAACCCAAAAGG

XM_011759130 GAACAUAGAGAUUUUAUAGGACUGGAUUAUUCCUCUUUGUAUAUGUGUAAACCCAAAAGG

XM_007967990 GAACAUAGAGAUUUUAUAGGACUGGACUAUUCCUCUUUGUAUAUGUGUAAACCCAAAAGG

XM_023209042 GAACAUAGAGAUUUUAUAGGACUGGACUAUUCCUCUUUGUAUAUGUGUAAACCCAAAAGG

XM_025402281 GAACAUAGAGAUUUUAUAGGACUGGAUUAUUCCUCUUUGUAUAUGUGUAAACCCAAAAGG

XM_017507035 GAACACAGAGAUUUUAUAGGACUGGACUAUUCCGCCUUGUAUAUGUGUAAACCCAAAAGG

XM_012739537 GAACAUAGAGAUUUUAUAGGACUGGAUUAUUCCUCUUUGUAUAUGUGUAAACCCAAAAGG

XM_007446307 GAACAUAGAGAUUUUAUAGGACUGGAUUAUCCCUCUUUGUAUAUGUGUAAGCCCAAAAGG

XM_024128992 GAACAUAGAGAUUUUAUAGGACUGGAUUAUCCCUCUUUGUAUAUGUGUAAGCCCAAAAGG

XM_027129408 GAACAUAGAGAUUUUAUAGGACUGGAUUAUCCCUCUUUGUAUAUGUGUAAGCCGAAAAGG

XM_025879601 GAACAUAGAGAUUUUAUAGGACUGGACUAUUCCUCUUUGUAUAUGUGUAAGCCCAAAAGG

XM_022577811 GAACAUAGAGAUUUUAUAGGACUGGAUUAUCCCUCUUUGUAUAUGUGUAAGCCCAAAAGG

XM_019936346 GAACAUAGAGAUUUUAUAGGACUGGAUUAUCCCUCUUUGUAUAUGUGUAAGCCGAAAAGG

XM_027593397 GAACAUAGAGAUUUUAUAGGACUGGACUAUUCCUCUUUGUAUAUGUGUAAGCCCAAAAGG

XM_015093964 GAACAUAGAGAUUUUAUAGGACUGGAUUAUCCCUCUUUGUAUAUGUGUAAGCCCAAAAGG

XM_019452268 GAACAUAGAGAUUUUAUAGGACUGGACUAUUCCUCUUUGUAUAUGUGUAAGCCCAAAAGG

XM_004270956 GAACAUAGAGAUUUUAUAGGACUGGAUUAUCCCUCUUUGUAUAUGUGUAAGCCGAAAAGG

XM_027541573 GAACAUAGAGAUUUUAUAGGACUGGAUUAUCCCUCUUUGUAUAUGUGUAAGCCCAAAAGG

XM_027934162 GAACAUAGAGAUUUUAUAGGACUGGACUAUUCUUCUUUGUAUAUGUGUAAACCCAAAAGG

XM_003355541 GAACAUAGAGAUUUUAUAGGACUGGAUUAUUCCUCUUUGUAUAUGUGUAAGCCCAAAAGG

XM_016119294 GAACAUAGAGAUUUUAUAGGACUGGACUAUUCCUCUUUGUAUAUGUGCAAACCCAAAAGG

XM_006127674 GAACAUGUGGAUUUUAUAGGACUGGACUAUCCAUCCUUGUAUUUGUGCAAACCCAAAAGA

NM_030762 AGCAUGAAACGAGACGACACCAAGGAUACCUACAAAUUACCGCACAGAUUAAUAGAAAAG

XM_520805 AGCAUGAAACGAGACGACACCAAGGAUACCUACAAAUUACCGCACAGAUUAAUAGAAAAG

XM_019037881 AGCAUGAAACGAGACGACACCAAGGAUACCUACAAAUUACCGCACAGAUUAAUAGAAAAG

XM_002823045 AGUAUGAAACGAGACGACACCAAGGAUACCUACAAAUUACCGCACAGAUUAAUAGAAAAG

XM_005570417 AGCAUGAAACGAGACGAUACCAAGGAUACCUACAAAUUACCGCACAGAUUAAUAGAAAAG

XM_012093655 AGCAUGAAACGAGACGAUACCAAGGAUACCUACAAAUUACCGCACAGAUUAAUAGAAAAG

XM_015151321 AGCAUGAAACGAGACGAUACCAAGGAUACCUACAAAUUACCGCACAGAUUAAUAGAAAAG

XM_011759130 AGCAUGAAACGAGACGAUACCAAGGAUACCUACAAAUUACCGCACAGAUUAAUAGAAAAG

XM_007967990 AGCAUGAAACGAGACGAUACCAAGGAUACCUACAAAUUACCGCACAGAUUAAUAGAAAAG

XM_023209042 AGCAUGAAACGAGACGAUACCAAGGAUACCUACAAAUUACCGCACAGAUUAAUAGAAAAG

XM_025402281 AGCAUGAAACGAGACGAUACCAAGGAUACCUACAAAUUACCGCACAGAUUAAUAGAAAAG

XM_017507035 AGCAUGAAACGAGACGACACCAAGGAUACCUACAAAUUACCGCACAGAUUAAUAGAAAAG

XM_012739537 AGCAUGAAACGAGACGACAGCAAGGAUACCUACAAAUUACCGCACAGAUUAAUAGAAAAG

XM_007446307 AGCAUGAAGCGAGACGAUAGCAAGGAUACCUACAAAUUACCGCACAGAUUAAUAGAAAAG

XM_024128992 AGCAUGAAGCGAGACGAUAGCAAGGAUACCUACAAAUUACCGCACAGAUUAAUAGAAAAG

XM_027129408 AGCAUGAAGCGAGACGACAGCAAGGAUACCUACAAAUUACCGCACAGAUUAAUAGAAAAG

XM_025879601 AGCAUGAAGCGAGACGAUAGCAAGGAUACCUACAAAUUACCGCACAGAUUAAUAGAAAAG

XM_022577811 AGCAUGAAGCGAGACGAUAGCAAGGAUACCUACAAAUUACCGCACAGAUUAAUAGAAAAG

XM_019936346 AGCAUGAAGCGAGACGACAGCAAGGAUACCUACAAAUUACCGCACAGAUUAAUAGAAAAG

XM_027593397 AGCAUGAAGCGAGACGAUAGCAAGGAUACCUACAAAUUACCGCACAGAUUAAUAGAAAAG

XM_015093964 AGCAUGAAGCGAGACGAUAGCAAGGAUACCUACAAAUUACCGCACAGAUUAAUAGAAAAG

XM_019452268 AGCAUGAAGCGAGACGAUAGCAAGGAUACCUACAAAUUACCGCACAGAUUAAUAGAAAAG

XM_004270956 AGCAUGAAGCGAGACGACAGCAAGGAUACCUACAAAUUACCGCACAGAUUAAUAGAAAAG

XM_027541573 AGCAUGAAGCGAGACGAUAGCAAGGAUACCUACAAAUUACCGCACAGAUUAAUAGAAAAG

XM_027934162 AGCAUGAAGCGAGACGACAGCAAGGAUACCUACAAAUUACCGCACAGAUUAAUAGAAAAG

XM_003355541 AGCAUGAAGCGAGACGAUAGCAAGGAUACCUACAAAUUACCGCACAGAUUAAUAGAAAAG

XM_016119294 AGCAUGAAGCGAGAUGAUAGCAAGGAUACCUACAAGUUACCACACAGAUUAAUAGAAAAG

XM_006127674 GGCAUGAAAAGAGAUGAGAGUAAGGAAACAUACAAACUGCCACAUAGACUGAUAGAAAAG

NM_030762 AAAAGAAGAGACCGAAUUAAUGAAUGCAUUGCUCAGCUGAAAGAUUUACUGCCUGAACAU

XM_520805 AAAAGAAGAGACCGAAUUAAUGAAUGCAUUGCUCAGCUGAAAGAUUUACUGCCUGAACAU

XM_019037881 AAAAGAAGAGACCGAAUUAAUGAAUGCAUUGCUCAGCUGAAAGAUUUACUGCCUGAACAU

XM_002823045 AAAAGAAGAGACCGAAUUAAUGAAUGCAUUGCUCAGCUGAAAGAUUUACUGCCUGAACAU

XM_005570417 AAAAGAAGAGACCGAAUUAAUGAAUGCAUUGCUCAGCUGAAAGAUUUACUGCCUGAACAU

XM_012093655 AAAAGAAGAGACCGAAUUAAUGAAUGCAUUGCUCAGCUGAAAGAUUUACUGCCUGAACAU

XM_015151321 AAAAGAAGAGACCGAAUUAAUGAAUGCAUUGCUCAGCUGAAAGAUUUACUGCCUGAACAU

XM_011759130 AAAAGAAGAGACCGAAUUAAUGAAUGCAUUGCUCAGCUGAAAGAUUUACUGCCUGAACAU

XM_007967990 AAAAGAAGAGACCGAAUUAAUGAAUGCAUUGCUCAGCUGAAAGAUUUACUGCCUGAACAU

XM_023209042 AAAAGAAGAGACCGAAUUAAUGAAUGCAUUGCUCAGCUGAAAGAUUUACUGCCUGAACAU

XM_025402281 AAAAGAAGAGACCGAAUUAAUGAAUGCAUUGCUCAGCUGAAAGAUUUACUGCCUGAACAU

XM_017507035 AAAAGAAGAGACCGAAUUAAUGAAUGCAUUGCUCAGCUGAAAGAUUUACUGCCUGAACAU

XM_012739537 AAAAGAAGAGACCGAAUUAAUGAAUGCAUUGCUCAGCUGAAAGAUUUACUGCCUGAACAU

XM_007446307 AAAAGAAGAGACCGAAUUAAUGAAUGCAUCGCUCAGCUGAAAGACUUACUGCCUGAACAU

XM_024128992 AAAAGAAGAGACCGAAUUAAUGAAUGCAUCGCUCAGCUUAAAGACUUACUGCCUGAACAU

XM_027129408 AAAAGAAGAGACCGAAUUAAUGAAUGCAUCGCUCAGCUGAAAGACUUACUGCCUGAACAU

XM_025879601 AAAAGAAGAGAUCGAAUUAAUGAAUGCAUUGCUCAGCUGAAAGAUUUACUGCCUGAACAU

XM_022577811 AAAAGAAGAGACCGAAUUAAUGAAUGCAUCGCUCAGCUGAAAGACUUACUGCCUGAACAU

XM_019936346 AAAAGAAGAGACCGAAUUAAUGAAUGCAUCGCUCAGCUGAAAGACUUACUGCCUGAACAU

XM_027593397 AAAAGAAGAGAUCGAAUUAAUGAAUGCAUUGCUCAGCUGAAAGAUUUACUGCCUGAACAU

XM_015093964 AAAAGAAGAGACCGAAUUAAUGAAUGCAUUGCUCAGCUGAAAGACUUACUGCCUGAACAU

XM_019452268 AAAAGAAGAGAUCGAAUUAAUGAAUGCAUUGCUCAGCUGAAAGAUUUACUGCCUGAACAU

XM_004270956 AAAAGAAGAGACCGAAUUAAUGAAUGCAUCGCUCAGCUGAAAGACUUACUGCCUGAACAU

XM_027541573 AAAAGAAGAGACCGAAUUAAUGAAUGCAUUGCUCAGCUGAAAGACUUACUGCCUGAACAU

XM_027934162 AAAAGAAGAGACCGAAUUAAUGAAUGCAUUGCUCAGCUGAAAGAUUUACUGCCUGAACAU

XM_003355541 AAAAGAAGAGACCGAAUUAAUGAAUGCAUUGCUCAGCUGAAAGACUUACUGCCUGAACAU

XM_016119294 AAAAGAAGAGACCGAAUUAAUGAAUGCAUUGCUCAGCUGAAAGAUUUACUGCCUGAACAU

XM_006127674 AAGAGGCGAGACAGGAUAAAUGAAUGCAUUGCUCAGCUAAAAGAUUUAUUGCCUGAGCAU

NM_030762 CUGAAAUUGACAACUCUGGGACAUCUGGAGAAAGCUGUAGUCUUGGAAUUAACUUUGAAA

XM_520805 CUGAAAUUGACAACUCUGGGACAUCUGGAGAAAGCUGUAGUCUUGGAAUUAACUUUGAAA

XM_019037881 CUGAAAUUGACAACUCUGGGGCAUCUGGAGAAAGCUGUAGUCUUGGAAUUAACUUUGAAA

XM_002823045 CUGAAAUUGACAACUCUGGGGCAUCUGGAGAAAGCUGUAGUCUUGGAAUUAACUUUGAAA

XM_005570417 CUGAAAUUGACAACUCUGGGGCAUCUGGAGAAAGCCGUAGUCUUGGAAUUAACUCUGAAA

XM_012093655 CUGAAAUUGACAACUCUGGGGCAUCUGGAGAAAGCCGUAGUCUUGGAAUUAACUCUGAAA

XM_015151321 CUGAAAUUGACAACUCUGGGGCAUCUGGAGAAAGCCGUAGUCUUGGAAUUAACUCUGAAA

XM_011759130 CUGAAAUUGACAACUCUGGGGCAUCUGGAGAAAGCCGUAGUCUUGGAAUUAACUCUGAAA

XM_007967990 CUGAAACUGACAACUCUGGGGCAUCUGGAGAAAGCCGUAGUCUUGGAAUUAACUCUGAAA

XM_023209042 CUGAAAUUGACAACUCUGGGGCAUCUGGAGAAAGCCGUAGUCUUGGAAUUAACUCUGAAA

XM_025402281 CUGAAAUUGACAACUCUGGGGCAUCUGGAGAAAGCCGUAGUCUUGGAAUUAACUCUGAAA

XM_017507035 CUGAAAUUGACAACUCUGGGGCAUCUGGAGAAAGCCGUAGUCUUGGAAUUAACUUUGAAA

XM_012739537 CUGAAAUUGACAACUCUGGGGCAUCUGGAGAAAGCUGUUGUCUUGGAAUUAACUUUGAAA

XM_007446307 CUAAAGUUGACAACUCUGGGGCACCUGGAGAAAGCGGUGGUCUUGGAAUUAACUUUGAAA

XM_024128992 CUAAAGUUGACAACUCUGGGGCACCUGGAGAAAGCGGUGGUCUUGGAAUUAACUUUGAAA

XM_027129408 CUAAAGUUGACAACUCUGGGGCACCUGGAGAAAGCAGUGGUCUUGGAAUUAACUUUGAAA

XM_025879601 CUGAAGUUGACAACUCUGGGGCAUCUGGAGAAAGCGGUAGUCUUGGAAUUAACUUUGAAA

XM_022577811 CUAAAGUUGACAACUCUGGGGCACCUGGAGAAAGCAGUGGUCUUGGAAUUAACUUUGAAA

XM_019936346 CUAAAGUUGACAACUCUGGGGCACCUGGAGAAAGCAGUGGUCUUGGAAUUAACUUUGAAA

XM_027593397 CUGAAGUUGACAACUCUGGGGCAUCUGGAGAAAGCGGUAGUCUUGGAAUUAACUUUGAAA

XM_015093964 CUAAAGUUGACAACUCUGGGGCAUCUGGAGAAAGCGGUAGUCCUGGAAUUAACUUUGAAA

XM_019452268 CUGAAGUUGACAACUCUGGGGCAUCUGGAGAAAGCGGUAGUAUUGGAAUUAACUUUGAAA

XM_004270956 CUAAAGUUGACAACUCUGGGGCACCUGGAGAAAGCAGUGGUCUUGGAAUUAACUUUGAAA

XM_027541573 CUAAAGUUGACAACUCUGGGGCAUCUGGAGAAAGCUGUAGUCCUGGAAUUAACUUUGAAA

XM_027934162 CUGAAAUUGACAACACUAGGGCAUCUGGAGAAAGCGGUAGUCUUGGAAUUAACUUUGAAA

XM_003355541 CUAAAGUUGACAACUCUGGGGCAUCUGGAGAAAGCGGUAGUAUUGGAAUUGACUUUGAAA

XM_016119294 CUGAAGUUGACAACUCUGGGGCAUCUGGAGAAAGCGGUAGUCUUGGAAUUAACUUUGAAA

XM_006127674 CUGAAAUUGACGACUCUGGGGCAUCUGGAGAAAGCUGUAGUUUUGGAAUUAACUUUGAAA

NM_030762 CACUUAAAAGCUUUAACCGCCUUAACCGAGCAACAGCAUCAGAAGAUAAUUGCUUUACAG

XM_520805 CACUUAAAAGCUUUAACCGCCUUAACCGAGCAACAGCAUCAGAAGAUAAUUGCUUUACAG

XM_019037881 CACUUAAAAGCUUUAACCGCCUUAACCGAGCAACAGCAUCAGAAGAUAAUUGCUUUACAG

XM_002823045 CACUUAAAAGCUUUAACCGCCUUAACCGAGCAACAGCAUCAGAAGAUAAUUGCUUUACAG

XM_005570417 CACCUAAAAGCUUUAACCGCCUUAACCGAGCAACAGCAUCAGAAGAUAAUUGCUUUACAG

XM_012093655 CACCUAAAAGCUUUAACCGCCUUAACCGAGCAACAGCAUCAGAAGAUAAUUGCUUUACAG

XM_015151321 CACCUAAAAGCUUUAACCGCCUUAACCGAGCAACAGCAUCAGAAGAUAAUUGCUUUACAG

XM_011759130 CACCUAAAAGCUUUAACCGCCUUAACCGAGCAACAGCAUCAGAAGAUAAUUGCUUUACAG

XM_007967990 CACCUAAAAGCUUUAACCGCCUUAACCGAGCAACAGCAUCAGAAGAUAAUUGCUUUACAG

XM_023209042 CACUUAAAAGCUUUAACCGCCUUAACCGAGCAACAGCACCAGAAGAUAAUUGCUUUACAG

XM_025402281 CACCUAAAAGCUUUAACCGCCUUAACCGAGCAACAGCAUCAGAAGAUAAUUGCUUUACAG

XM_017507035 CACUUAAAAGCUUUAACCGCCUUAACCGAGCAGCAGCAUCAGAAGAUAAUUGCUUUACAG

XM_012739537 CACUUAAAAGCUUUAACCGCCUUAACCGAGCAGCAGCAUCAGAAGAUAAUUGCUUUACAG

XM_007446307 CACUUAAAAGCUUUAACAGCCUUAACGGAGCAGCAGCAUCAGAAGAUAAUUGCUUUACAG

XM_024128992 CACUUAAAAGCUUUAACAGCCUUAACGGAGCAGCAGCAUCAGAAGAUAAUUGCUUUACAG

XM_027129408 CACUUAAAAGCUUUAACAGCCUUAACGGAGCAGCAGCAUCAGAAGAUAAUUGCUUUACAG

XM_025879601 CACUUAAAAGCUUUAACAGCCUUAACCGAGCAGCAGCAUCAGAAGAUAAUUGCUUUACAG

XM_022577811 CACUUAAAAGCUUUAACAGCCUUAACGGAGCAGCAGCAUCAGAAGAUAAUUGCUUUACAG

XM_019936346 CACUUAAAAGCUUUAACAGCCUUAACGGAGCAGCAGCAUCAGAAGAUAAUUGCUUUACAG

XM_027593397 CACUUAAAAGCUUUAACAGCCUUAACCGAGCAGCAGCAUCAGAAGAUAAUUGCUUUACAG

XM_015093964 CACUUAAAAGCUUUGACAGCCUUAACGGAGCAGCAACAUCAGAAGAUAAUUGCUUUACAG

XM_019452268 CACUUAAAAGCUUUAACAGCCUUAACCGAGCAGCAGCAUCAGAAGAUAAUUGCUUUACAG

XM_004270956 CACUUAAAAGCUUUAACAGCCUUAACGGAGCAGCAGCAUCAGAAGAUAAUUGCUUUACAG

XM_027541573 CACUUAAAAGCUUUGACAGCCUUAACGGAGCAGCAACAUCAGAAGAUAAUUGCUUUACAG

XM_027934162 CACUUAAAAGCUUUAACAGCCUUAACCGAGCAACAGCAUCAGAAGAUAAUUGCUUUACAG

XM_003355541 CACUUAAAAGCUUUAACAGCCUUAACAGAGCAGCAGCAUCAGAAGAUAAUUGCUUUACAG

XM_016119294 CACUUAAAAGCUUUAACAGCCUUAACCGAGCAGCAGCAUCAGAAGAUAAUUGCUUUACAG

XM_006127674 CACUUAAAAGCUUUAACAGCCUUAACGGAGCAACAGCAUCAGAAUAUAAUUGCUUUACAG

NM_030762 AAUGGGGAGCGAUCUCUGAAAUCGCCCAUUCAGUCCGACUUGGAUGCGUUCCACUCGGGA

XM_520805 AAUGGGGAGCGAUCUCUGAAAUCGCCCAUUCAGUCCGACUUGGAUGCGUUCCACUCGGGA

XM_019037881 AAUGGGGAGCGAUCUCUGAAAUCGCCCAUUCAGUCCGACUUGGAUGCGUUCCACUCGGGA

XM_002823045 AAUGGGGAGCGAUCUCUGAAAUCGCCCAUUCAGUCCGAUUUGGAUGCGUUCCACUCGGGA

XM_005570417 AAUGGGGAGCGAUCUCUGAAAUCGCCCAUUCAGUCCGACUUGGAUGCGUUCCACUCGGGA

XM_012093655 AAUGGGGAGCGAUCUCUGAAAUCGCCCAUUCAGUCCGACUUGGAUGCGUUCCACUCGGGA

XM_015151321 AAUGGGGAGCGAUCUCUGAAAUCGCCCAUUCAGUCCGACUUGGAUGCGUUCCACUCGGGA

XM_011759130 AAUGGGGAGCGAUCUCUGAAAUCGCCCAUUCAGUCCGACUUGGAUGCGUUCCACUCGGGA

XM_007967990 AAUGGGGAGCGAUCUCUGAAAUCGCCCAUUCAGUCCGACUUGGAUGCGUUCCACUCGGGA

XM_023209042 AAUGGGGAGCGAUCUCUGAAAUCGCCCAUUCAGUCCGACUUGGAUGCGUUCCACUCGGGA

XM_025402281 AAUGGGGAGCGAUCUCUGAAAUCGCCCAUUCAGUCCGACUUGGAUGCGUUCCACUCGGGA

XM_017507035 AAUGGGGAGCGAUCUCUGAAAUCGCCCAUUCAGUCCGACUUGGAUGCGUUCCACUCGGGA

XM_012739537 AAUGGGGAGCGAUCUCUGAAAUCGCCCAUUCAGUCUGACUUGGAUGCGUUCCACUCGGGA

XM_007446307 AAUGGGGAGCGAUCUCUGAAAUCGCCCAUUCAGUCCGACUUGGAUGCGUUCCAUUCGGGA

XM_024128992 AAUGGGGAGCGAUCUCUGAAAUCGCCCAUUCAGUCCGACUUGGAUGCGUUCCAUUCGGGA

XM_027129408 AAUGGGGAGCGAUCUCUGAAAUCGCCCAUUCAGUCCGACUUGGAUGCGUUCCAUUCGGGA

XM_025879601 AAUGGGGAGCGAUCUCUGAAAUCGCCCAUUCAGUCCGACUUGGAUGCGUUCCACUCGGGA

XM_022577811 AAUGGGGAGCGAUCUCUGAAAUCGCCCAUUCAGUCCGACUUGGAUGCGUUCCAUUCGGGA

XM_019936346 AAUGGGGAGCGAUCUCUGAAAUCGCCCAUUCAGUCCGACUUGGAUGCGUUCCAUUCGGGA

XM_027593397 AAUGGGGAGCGAUCUCUGAAAUCGCCCAUUCAGUCCGACUUGGAUGCGUUCCACUCGGGA

XM_015093964 AAUGGGGAGCGAUCUCUGAAAUCGCCCAUUCAGUCCGACUUGGAUGCGUUCCACUCGGGA

XM_019452268 AAUGGGGAGCGAUCUCUGAAAUCGCCCAUUCAGUCCGACUUGGAUGCGUUCCACUCCGGA

XM_004270956 AAUGGGGAGCGAUCUCUGAAAUCGCCCAUUCAGUCCGACUUGGAUGCGUUCCAUUCGGGA

XM_027541573 AAUGGGGAACGAUCUCUGAAAUCGCCCAUUCAGUCCGACUUGGAUGCGUUCCACUCGGGA

XM_027934162 AAUGGGGAGCGAUCUCUGAAAUCGCCCAUUCAGUCCGACUUGGAUGCGUUCCACUCGGGA

XM_003355541 AAUGGGGAGCGAUCUUUGAAAUCGCCCAUUCAGUCCGACUUGGAUGCGUUCCACUCGGGA

XM_016119294 AAUGGGGAGCGAUCUCUGAAAUCGCCCAUUCAGUCCGACUUGGAUGCGUUCCACUCGGGA

XM_006127674 AAUGGGGAGCGGGCUAUGAAGUCCCCCAUUCAGUGCGACCUGGAUGCUUUCCAUUCGGGA

NM_030762 UUUCAAACAUGCGCCAAAGAAGUCUUGCAAUACCUCUCCCGGUUUGAGAGCUGGACACCC

XM_520805 UUUCAAACAUGCGCCAAAGAAGUCUUGCAAUACCUCUCCCGGUUUGAGAGCUGGACACCC

XM_019037881 UUUCAAACAUGCGCCAAAGAAGUCUUGCAAUACCUCUCCCGGUUUGAGAGCUGGACACCC

XM_002823045 UUUCAAACAUGCGCCAAAGAAGUCUUGCAAUACCUCUCCCGGUUUGAGAGCUGGACACCC

XM_005570417 UUUCAAACAUGCGCCAAAGAAGUCUUGCAAUACCUCUCCCGGUUUGAGAGCUGGACACCC

XM_012093655 UUUCAAACAUGCGCCAAAGAAGUCUUGCAAUACCUCUCCCGGUUUGAGAGCUGGACACCC

XM_015151321 UUUCAAACAUGCGCCAAAGAAGUCUUGCAAUACCUCUCCCGGUUUGAGAGCUGGACACCC

XM_011759130 UUUCAAACAUGCGCCAAAGAAGUCUUGCAAUACCUCUCCCGGUUUGAGAGCUGGACACCC

XM_007967990 UUUCAAACAUGCGCCAAAGAAGUCUUGCAAUACCUCUCCCGGUUUGAGAGCUGGACACCC

XM_023209042 UUUCAAACAUGCGCCAAAGAAGUCUUGCAAUACCUCUCCCGGUUUGAGAGCUGGACACCC

XM_025402281 UUUCAAACAUGCGCCAAAGAAGUCUUGCAAUACCUCUCCCGGUUUGAGAGCUGGACACCC

XM_017507035 UUUCAAACAUGCGCCAAAGAAGUCUUGCAAUACCUCUCCCGGUUUGAGAGCUGGACACCC

XM_012739537 UUUCAAACAUGCGCCAAAGAAGUCUUGCAAUACCUCUCCCGGUUUGAGAGCUGGACACCC

XM_007446307 UUUCAAACAUGCGCCAAAGAAGUCUUGCAAUACCUCGCCCGGUUUGAGAGCUGGACGCCC

XM_024128992 UUUCAAACAUGCGCCAAAGAAGUCUUGCAAUACCUCGCCCGGUUUGAGAGCUGGACGCCC

XM_027129408 UUUCAAACAUGCGCCAAAGAAGUCUUGCAAUACCUCGCCCGGUUUGAGAGCUGGACGCCC

XM_025879601 UUUCAAACAUGCGCCAAAGAAGUCUUGCAAUACCUCUCCCGGUUUGAGAGCUGGACGCCC

XM_022577811 UUUCAAACAUGCGCCAAAGAAGUCUUGCAAUACCUCGCCCGGUUUGAGAGCUGGACGCCC

XM_019936346 UUUCAAACAUGCGCCAAAGAAGUCUUGCAAUACCUCGCCCGGUUUGAGAGCUGGACGCCG

XM_027593397 UUUCAAACAUGCGCCAAAGAAGUCUUGCAAUACCUCUCCCGGUUUGAGAGCUGGACGCCC

XM_015093964 UUUCAAACAUGCGCCAAAGAAGUCUUGCAAUACCUCGCCCGGUUUGAGAGCUGGACGCCC

XM_019452268 UUUCAAACAUGCGCCAAAGAAGUCUUGCAAUACCUCUCCCGGUUUGAGAGCUGGACGCCC

XM_004270956 UUUCAAACAUGCGCCAAAGAAGUCUUGCAAUACCUCGCCCGGUUUGAGAGCUGGACGCCC

XM_027541573 UUUCAAACAUGCGCCAAAGAAGUCUUGCAAUACCUCGCCCGGUUUGAGAGCUGGACGCCC

XM_027934162 UUUCAAACAUGCGCCAAAGAAGUCUUGCAAUACCUCUCCCGGUUUGAGAGCUGGACACCC

XM_003355541 UUUCAAACAUGCGCCAAAGAAGUCUUGCAAUACCUCGCCCGGUUUGAGAGCUGGACGCCC

XM_016119294 UUUCAAACAUGCGCCAAAGAAGUCUUGCAAUACCUCUCCCGGUUUGAGAGCUGGACGCCC

XM_006127674 UUUCAAACGUGCGCCAAAGAAGUCUUGCAGUACCUCUCCCGGUUUGAAAGUUGGACUCCC

NM_030762 AGGGAGCCGCGGUGUGUCCAGCUGAUCAACCACUUGCACGCCGUGGCCACCCAGUUCUUG

XM_520805 AGGGAGCCGCGGUGUGUCCAGCUGAUCAACCACUUGCACGCCGUGGCCACCCAGUUCUUG

XM_019037881 AGGGAGCCGCGGUGUGUCCAGCUGAUCAACCACUUGCACGCCGUGGCCACCCAGUUCUUG

XM_002823045 AGGGAGCCGCGGUGUGUCCAGCUGAUCAACCACUUGCACGCCGUGGCCACCCAGUUCUUG

XM_005570417 AGGGAGCCGCGGUGUGUCCAGCUGAUCAACCACUUGCACGCCGUGGCCACCCAGUUCUUG

XM_012093655 AGGGAGCCGCGGUGUGUCCAGCUGAUCAACCACUUGCACGCCGUGGCCACCCAGUUCUUG

XM_015151321 AGGGAGCCGCGGUGUGUCCAGCUGAUCAACCACUUGCACGCCGUGGCCACCCAGUUCUUG

XM_011759130 AGGGAGCCGCGGUGUGUCCAGCUGAUCAACCACUUGCACGCCGUGGCCACCCAGUUCUUG

XM_007967990 AGGGAGCCGCGGUGUGUCCAGCUGAUCAACCACUUGCACGCCGUGGCCACCCAGUUCUUG

XM_023209042 AGGGAGCCGCGGUGUGUCCAGCUGAUCAACCACUUGCACGCCGUGGCCACCCAGUUGUUG

XM_025402281 AGGGAGCCGCGGUGUGUCCAGCUGAUCAACCACUUGCACGCCGUGGCCACCCAGUUCUUG

XM_017507035 AGGGAGCCGCGGUGUGUCCAGCUGAUCAACCACUUGCACGCCGUGGCCACCCAGUUCUUG

XM_012739537 AGGGAGCCGCGGUGCGUCCAGCUGAUCAACCACUUGCACGCCGUGGCCACCCAGUUCUUG

XM_007446307 AGGGAGCCGCGGUGCGUCCAGCUGAUCAACCACUUGCACGCCGUGGCCACCCAGUUCUUG

XM_024128992 AGGGAGCCGCGGUGUGUCCAGCUGAUCAACCACUUGCACGCCGUGGCCACCCAGUUCUUG

XM_027129408 AGGGAGCCGCGGUGUGUCCAGCUGAUCAACCACUUGCACGCCGUGGCCACCCAGUUCUUG

XM_025879601 AGAGAGCAGCGGUGUGUCCAGCUGAUCAACCACUUGCACGCCGUGGCCACCCAGUUCUUG

XM_022577811 AGGGAGCCGCGGUGUGUCCAGCUGAUCAACCACUUGCACGCCGUGGCCACCCAGUUCUUG

XM_019936346 AGGGAGCCGCGGUGUGUCCAGCUGAUCAACCACUUGCACGCCGUGGCCACCCAGUUCUUG

XM_027593397 AGAGAGCAGCGGUGUGUCCAGCUGAUCAACCACUUGCACGCCGUGGCCACCCAGUUCUUG

XM_015093964 AGGGAGCCGCGGUGUGUCCAGCUGAUCAACCACUUGCACGCCGUGGCCACCCAGUUCUUG

XM_019452268 AGGGAGCAGCGGUGUGUCCAGCUGAUCAACCACUUGCACGCCGUGGCCACCCAGUUCUUG

XM_004270956 AGGGAGCCGCGGUGUGUCCAGCUGAUCAACCACUUGCACGCCGUGGCCACCCAGUUCUUG

XM_027541573 AGGGAGCCGCGGUGUGUCCAGCUGAUCAACCACUUGCACGCCGUGGCCACCCAGUUCUUG

XM_027934162 AGGGAGCCGCGGUGUGUCCAGCUAAUCAACCACUUGCACGCCGUGGCCACCCAGUUUUUG

XM_003355541 AGGGAGCCUCGGUGUGUCCAGCUGAUCAACCACUUGCACGCCGUGGCCACCCAGUUCUUA

XM_016119294 AGGGAACCGCGGUGUGUCCAGCUGAUCAACCACUUGCAUGCGGUGGCCACCCAGUUCUUG

XM_006127674 AGGGAGCAGAGAUGCGCCCAGCUCGUGAACCAUCUGCACGCGGUUUCCACUCAGUUCUUA

NM_030762 CCCACCCCGCAGCUGUUGACUCAACAGGUCCCUCUGAGCAAAGGCACCGGCGCUCCCUCG

XM_520805 CCCACCCCGCAGCUGUUGACUCAACAGGUCCCUCUGAGCAAAGGCACCGGCGCUCCCUCG

XM_019037881 CCCACCCCGCAGCUGUUGACUCAACAGGUCCCUCUGAGUAAAGGCACCGGCGCUCCCUCG

XM_002823045 CCCACCCCGCAGCUGUUGACUCAACAGGUCCCUCUGAGCAAAGGCACCGGCGCUCCCUCG

XM_005570417 CCCACCCCGCAGCUGUUGACUCAACAGGUCCCUCUGAGCAAAGGCACCGGCGCUCCCUCG

XM_012093655 CCCACCCCGCAGCUGUUGACUCAACAGGUCCCUCUGAGCAAAGGCACCGGCGCUCCCUCG

XM_015151321 CCCACCCCGCAGCUGUUGACUCAACAGGUCCCUCUGAGCAAAGGCACCGGCGCUCCCUCG

XM_011759130 CCCACCCCGCAGCUGUUGACUCAACAGGUCCCUCUGAGCAAAGGCACCGGCGCUCCCUCG

XM_007967990 CCCACCCCGCAGCUGUUGACUCAACAGGUCCCUCUGAGCAAAGGCACCGGCGCUCCCUCG

XM_023209042 CCCACCCCGCAGCUGUUGACUCAACAGGUCCCUCUGAGCAAAGGCACCGGCGCUCCCUCG

XM_025402281 CCCACCCCGCAGCUGUUGACUCAACAGGUCCCUCUGAGCAAAGGCACCGGCGCUCCCUCG

XM_017507035 CCCACCCCUCAGCUGUUGACUCAACAGGUCCCUCUGAGCAAAGGCACCGGCGCUCCCUCG

XM_012739537 CCCACCCCGCAGCUGUUGACUCAACAGGUCCCUCUGAGCAAAGGCGCGGGCGCCGCCUCG

XM_007446307 CCCACCCCCCAGCUGUUGACUCAACAGGUUCCUCUGAGCAAAGGCACCGGCGUGCCCUCG

XM_024128992 CCCACCCCCCAGCUGUUGACUCAACAGGUUCCUCUGAGCAAAGGCACCGGCGUGCCCUCG

XM_027129408 CCCACCCCCCAGCUGUUGACUCAACAGGUUCCUCUGAGCAAAGGCACCGGCGUGCCCUCG

XM_025879601 CCCACCCCCCAGCUGUUGACUCAACAGGUCCCUCUGAGUAAAGGCACCGGCGCGCCCUCG

XM_022577811 CCCACCCCCCAGCUGUUGACUCAACAGGUUCCUCUGAGCAAAGGCACCGGCGUGCCCUCG

XM_019936346 CCCACCCCCCAGCUGUUGACUCAACAGGUUCCUCUGAGCAAAGGCACCGGCGUGCCCUCG

XM_027593397 CCCACCCCCCAGCUGUUGACUCAACAGGUCCCUCUGAGUAAAGGCACCGGCGCGCCCUCG

XM_015093964 CCCACCCCGCAGCUGUUGACUCAACAGGUUCCUCUGAGCAAAGGCACCGGCGCGCCCACG

XM_019452268 CCCACCCCUCAGCUGUUGACUCAACAGGUUCCUCUGAGCAAAGGCACUGGCGCGCCCUCG

XM_004270956 CCCACCCCCCAGCUGUUGACUCAACAGGUUCCUCUGAGCAAAGGCACCGGCGUGCCCUCG

XM_027541573 CCCACCCCGCAGCUGUUGACUCAACAGGUUCCUCUGAGCAAAGGCACCGCCGCGCCCACG

XM_027934162 CCCACCCCCCAGUUGUUGACUCAACAGGUCCCUUUGAGCAAAGGCACUGGCGCUCCCUCC

XM_003355541 CCCACCCCGCAGCUGUUGACUCAACAGGUUCCUCUGAGCAAAGGCACCGGCGCGCCCACC

XM_016119294 CCCACCCCUCAGCUGUUGACUCAACAGGUCCCUCUGAGCAAAGGCACGGGCGCGCCCUCG

XM_006127674 CCCAGCCCCCAGCUGUUGACUCCACAGGUCCCCGCGAGCAAAGGAUCCUCCUCCUCCUCU

NM_030762 GCCGCC---------GGGUCCGCGGCCGCCCCCUGCCUGGAGCGCGCGGGGCAGAAGCUG

XM_520805 GCCGCC---------GGGUCCGCGGCCGCCCCCUGCCUGGAGCGCGCGGGGCAGAAGCUG

XM_019037881 GCCGCC---------GGGUCCGCGGCCGCCCCCUGCCUGGAGCGCGCGGGGCAGAAGCUG

XM_002823045 GCCGC---------UGGGUCCGCGGCCGCCCCCUGCCUGGAGCGCGCGGGGCAGAAGCUG

XM_005570417 GCCGCC---------GGGUCCGCGGCCGCCCCCUGCCUGGAGCGCGCGGGGCAGAAGCUG

XM_012093655 GCCGCC---------GGGUCCGCGGCCGCCCCCUGCCUGGAGCGCGCGGGGCAGAAGCUG

XM_015151321 GCCGCC---------GGGUCCGCGGCCGCCCCCUGCCUGGAGCGCGCGGGGCAGAAGCUG

XM_011759130 GCCGCC---------GGGUCCGCGGCCGCCCCCUGCCUGGAGCGCGCGGGGCAGAAGCUG

XM_007967990 GCCGCC---------GGGUCCGCGGCCGCCCCCUGCCUGGAGCGCGCGGGGCAGAAGCUG

XM_023209042 GCCGCC---------GGGUCCGCGGCCGCCCCCUGCCUGGAGCGCACUGGGCAGAAGCUG

XM_025402281 GCCGCC---------GGGUCCGCGGCCGCCCCCUGCCUGGAGCGCGCGGGGCAGAAGCUG

XM_017507035 GCUGCC---------GGGUCCGCAACCGCCCCCUGCCUGGAGCGCGCGGCGCAGAAGCUG

XM_012739537 GCCGCCGCCCCCGCGGGGUCGGCCGCCGCGCCCUGCCUGGAGCGCGCGGGGCAGAAGCUG

XM_007446307 GCCGCCACCCCCGCCGGGUCCGGGGCCGCCCCCUGCCUGGAGCGCGCCGGGCAGAAGCUG

XM_024128992 GCCGCCGCCCCCGCCGGGUCCGGGGCCGCCCCCUGCCUGGAGCGCGCCGGGCAGAAGCUG

XM_027129408 GCCCCCACCCCCGCCGGGUCCGGGGCCGCCCCCUGCCUGGAGCGCGCCGGGCAGAAGCUG

XM_025879601 GCCGCUGCCCCCACAGGGUCCGCGGCCGCCCCCUGCCUGGAGCGCGCCGGGCAGAAGCUU

XM_022577811 GCCGCCACCCCCGCCGGGUCCGGGGCCGCCCCCUGCCUGGAGCGCGCCGGGCAGAAGCUG

XM_019936346 GCCGCCACCCCCGCCGGGUCCGGGGCCGCCCCCUGCCUGGAGCGCGCCGGGCAGAAGCUG

XM_027593397 GCCGCUGCCCCCACAGGGUCCGCGGCCGCCCCCUGCCUGGAGCGCGCCGGGCAGAAGCUU

XM_015093964 GCCGCCGCCCCCGCCGGCUCCGGGGCCGCCCCCUGCCUGGAGCGCGCGGGGCAGAAGCUG

XM_019452268 GCCGCCGCCCCCGCAGGGUCUGCGGCCGCCCCCUGCCUGGAGCGCGCCGGGCAGAAGCUU

XM_004270956 GCCGCCACCCCCGCCGGGUCCGGGGCCGCCCCCUGCCUGGAGCGCGCCGGGCAGAAGCUG

XM_027541573 GCGGCCGCCCCCGCCGGCUCCGGGGCCGCCCCCUGCCUGGAGCGCGCGGGGCAGAAGCUG

XM_027934162 GCCCCCGCCCCCACCGGGUCCACGGCCGCCCCCUGCCUGGAGCGCGCAGGGCAGAAGCUC

XM_003355541 ACCGCC---CCCGCCGGGUCCGUGGCCGCCGCUUGCCUGGAGCGCGCGGGGCAGAAGCUG

XM_016119294 GCCUCCGCCCCCGCCGGGUCCGCAGCAGCCCCCUGCCUGGAGCGCGCUGGGCAGAAGCUG

XM_006127674 UGUGCA------------------------------CAGGAUCGCACCGGGCAAAAGCUG

NM_030762 GAGCCCCUCGCCUACUGCGUGCCCGUCAUCCAGCGGACUCAGCCCAGCGCCGAGCUCGCC

XM_520805 GAGCCCCUCGCCUACUGCGUGCCGGUCAUCCAGCGGACUCAGCCCAGCGCCGAGCUCGCC

XM_019037881 GAGCCCCUCGCCUACUGCGUGCCGGUCAUCCAGCGGACUCAGCCCAGCGCCGAGCUCGCC

XM_002823045 GAGCCCCUUGCCUACUGCGUGCCGGUCAUCCAGCGGACUCAGCCCAGCGCCGAGCUCGCC

XM_005570417 GAGCCCCUCGCCUACUGCGUGCCGGUCAUCCAGCGGACUCAGCCCAGCGCCGAGCUCGCC

XM_012093655 GAGCCCCUCGCCUACUGCGUGCCGGUCAUCCAGCGGACUCAGCCCAGCGCCGAGCUCGCC

XM_015151321 GAGCCCCUCGCCUACUGCGUGCCGGUCAUCCAGCGGACUCAGCCCAGCGCCGAGCUCGCC

XM_011759130 GAGCCCCUCGCCUACUGCGUGCCGGUCAUCCAGCGGACUCAGCCCAGCGCCGAGCUCGCC

XM_007967990 GAGCCCCUCGCCUACUGCGUGCCGGUCAUCCAGCGGACUCAGCCCAGCGCCGAGCUCGCC

XM_023209042 GAGCCCCUCGCCUACUGCGUGCCGGUCAUCCAGCGGACUCAGCCCAACGCCGAGCUCGCC

XM_025402281 GAGCCCCUCGCCUACUGCGUGCCGGUCAUCCAGCGGACUCAGCCCAGCGCCGAGCUCGCC

XM_017507035 GAGCCCCUCGCCCAUUGCGUGCCGGUCAUCCAGCGGACUCAGCCUAGCGCCGAGCUCGCC

XM_012739537 GAGCCCCUGGCGCACUGCGUGCCGGUCAUCCAGCGGACUCAGCCCAGCGCCGAGCUCGCC

XM_007446307 GAGCCCCUCGCCCACUGCGUGCCGGUCAUCCAGCGGACUCAGCCCAGCGCCGAGCUCGCC

XM_024128992 GAGCCCCUCGCCCACUGCGUGCCGGUCAUCCAGCGGACUCAGCCCAGCGCCGAGCUCGCC

XM_027129408 GAGCCCCUCGCCCACUGCGUGCCGGUCAUCCAGCGGACUCAGCCCAGCGCCGAGCUCGCC

XM_025879601 GAGCCCCUCGCCCACUGCGUGCCGGUCAUCCAGCGGACUCAGCCCAGCGCCGAGCUCGCC

XM_022577811 GAGCCCCUCGCCCACUGCGUGCCGGUCAUCCAGCGGACUCAGCCCAGCGCCGAGCUCGCC

XM_019936346 GAGCCCCUCGCCCACUGCGUGCCGGUCAUCCAGCGGACUCAGCCCAGCGCCGAGCUCGCC

XM_027593397 GAGCCCCUCGCCCACUGCGUGCCGGUCAUCCAGCGGACUCAGCCCAGCGCCGAGCUCGCC

XM_015093964 GAACCCCUCGCCCACUGCGUGCCGGUCAUCCAGCGGACUCAGCCCAGCGCCGAGCUCGCC

XM_019452268 GAGCCCCUCGCCCACUGCGUCCCGGUCAUCCAGCGGACUCAGCCCAGCGCCGAGCUCGCC

XM_004270956 GAGCCCCUCGCCCACUGCGUGCCGGUCAUCCAGCGGACUCAGCCCAGCGCCGAGCUCGCC

XM_027541573 GAGCCCCUCGCCCACUGCGUGCCGGUCAUCCAGCGGACUCAGCCCAGCUCCGAGCUCGCC

XM_027934162 GAGCCCCUCGCCCACUGCGUGCCGGUCAUCCAGCGGACUCAGCCCAGCGCCGAGCUCGCC

XM_003355541 GAGCCCCUCGCCCACUGCGUGCCGGUCAUCCAGCGGACUCAGCCCAGCGCCGAGCUCGCC

XM_016119294 GAGCCCCUCGCCCACUGCGUUCCGGUCAUACAGCGGACUCAGCCCAGCGCCGAGCUCGCC

XM_006127674 GAGGCUCAGACUAACUGCGUGCCCGUCAUCCAGCGGACUCACCCGCCGGCCGAGCUGAGC

NM_030762 GCC---GAGAACGACACGGACACCGACAGCGGCUACGGCGGCGAAGCCGAGGCCCGGCCG

XM_520805 GCC---GAGAACGACACGGACACCGACAGCGGCUACGGCGGCGAAGCCGAGGCCCGGCCG

XM_019037881 GCC---GAGAACGACACGGACACCGACAGCGGCUACGGCGGCGAAGCCGAGGCCCGGCCG

XM_002823045 GCC---GAGAACGACACGGACACCGACAGCGGCUACGGCGGCGAAGCCGAGGCCCGGCCG

XM_005570417 GCC---GAGAACGACACGGACACCGACAGCGGCUACGGCGGCGAAGCCGAGGCCCGGCCG

XM_012093655 GCC---GAGAACGACACGGACACCGACAGCGGCUACGGCGGCGAAGCCGAGGCCCGGCCG

XM_015151321 GCC---GAGAACGACACGGACACCGACAGCGGCUACGGCGGCGAAGCCGAGGCCCGGCCG

XM_011759130 GCC---GAGAACGACACGGACACCGACAGCGGCUACGGCGGCGAAGCCGAGGCCCGGCCG

XM_007967990 GCC---GAGAACGACACGGACACCGACAGCGGCUACGGCGGCGAAGCCGAGGCCCGGCCG

XM_023209042 GCC---GAGAACGACACGGACACCGACAGCGGCUACGGCGGCGAAGCCGAGGCCCGGCCG

XM_025402281 GC---AGAGAACGACACGGACACCGACAGCGGCUACGGCGGCGAAGCCGAGGCCCGGCCG

XM_017507035 GCC---GAGAACGACACGGACACCGACAGCGGCUACGGCGGCGAAGCCGAGGCCCGGCCG

XM_012739537 GCC---GAGAACGACACGGACACCGACAGCGGCUACGGCGGCGAGGCCGAGGCCCGGCCG

XM_007446307 GCC---GAGAACGACACGGACACCGACAGCGGCUACGGCGGCGAGGCCGAGGCCCGGCCG

XM_024128992 GCC---GAGAACGACACGGACACCGACAGCGGCUACGGCGGCGAGGCCGAGGCCCGGCCG

XM_027129408 GCC---GAGAACGACACGGACACCGACAGCGGCUACGGCGGCGAGGCCGAGGCCCGGCCG

XM_025879601 GCC---GAGAACGACACGGACACCGACAGCGGCUACGGCGGCGAGGCCGAGGCGAGGCCG

XM_022577811 GCC---GAGAACGACACGGACACCGACAGCGGCUACGGCGGCGAGGCCGAGGCGAGGCCG

XM_019936346 GCC---GAGAACGACACGGACACCGACAGCGGCUACGGCGGCGAGGCCGAGGCCCGGCCG

XM_027593397 GCC---GAGAACGACACGGACACCGACAGCGGCUACGGCGGCGAGGCCGAGGCGAGGCCG

XM_015093964 GCCGCCGAGAACGACACGGACACCGAUAGCGGCUACGGUGGCGAGGCCGAGGCCCGGCCG

XM_019452268 GCC---GAGAACGACACGGACACCGACAGCGGCUACGGCGGCGAGGCCGAGGCUCGGCCG

XM_004270956 GCC---GAGAACGACACGGACACCGACAGCGGCUACGGCGGCGAGGCCGAGGCCCGGCCG

XM_027541573 GCCGCCGAGAACGACACGGACACCGAUAGCGGCUACGGUGGCGAGGCCGAGGCCCGGCCG

XM_027934162 GC---GGAGAACGACACGGACACUGACAGCGGCUACGGCGGAGAGGCCGAGGCCCGGCCA

XM_003355541 GC---UGAGAACGAUACAGACACGGACAGCGGCUACGGCGGCGAGGCCGAGGCCCGGCCA

XM_016119294 GCC---GAAAACGACACGGACACCGACAGCGGCUACGGCGGCGAAGCCGAGGCCAGGCCG

XM_006127674 G---GGGAGAAUGACACCGACACAGACAGCGGCUACGGAGGGGAGAGCGAGGGCAGGCCG

NM_030762 GACCGCGAGAAAGGCAAAGGCGCGGGGGCGAGCCGCGUCACCAUCAAGCAGGAGCCUCCC

XM_520805 GACCGCGAGAAAGGCAAAGGCGCGGGGGCGAGCCGCGUCACCAUCAAGCAGGAGCCUCCC

XM_019037881 GACCGCGAGAAAGGCAAAGGCGCGGGGGCGAGCCGCGUCACCAUCAAGCAGGAGCCUCCC

XM_002823045 GACCGCGAGAAAGGCAAAGGCGCGGGGGCGAGCCGCGUCACCAUCAAGCAGGAGCCUCCC

XM_005570417 GACCGCGAGAAAGGCAAAGGCGCGGGGGCGAGCCGCGUCACCAUCAAGCAGGAGCCUCCC

XM_012093655 GACCGCGAGAAAGGCAAAGGCGCGGGGGCGAGCCGCGUCACCAUCAAGCAGGAGCCUCCC

XM_015151321 GACCGCGAGAAAGGCAAAGGCGCGGGGGCGAGCCGCGUCACCAUCAAGCAGGAGCCUCCC

XM_011759130 GACCGCGAGAAAGGGAAAGGCGCGGGGGCGAGCCGCGUCACCAUCAAGCAGGAGCCUCCC

XM_007967990 GACCGCGAGAAAGGCAAAGGCGCGGGGGCGAGCCGCGUCACCAUCAAGCAGGAGCCUCCC

XM_023209042 GACCGCGAGAAAGGCAAAGGCGCGGGGGCGAGCCGCGUCACCAUCAAGCAGGAGCCUCCC

XM_025402281 GACCGCGAGAAAGGCAAAGGCGCGGGGGCGAGCCGCGUCACCAUCAAGCAGGAGCCUCCC

XM_017507035 GACCGCGAGAAGGGCAAAGGCGCGGGUGCGAGCCGCGUCACCAUCAAGCAGGAGCCUCCC

XM_012739537 GACCGCGGGAAGAGCAAAGGCGCGGGGGCGAGCCGCGUCACCAUCAAGCAGGAGCCGCCC

XM_007446307 GACCGCGAGAAGGGCAAAGGCGCGGGGGCAAGCCGCGUCACCAUCAAGCAGGAGCCCCCC

XM_024128992 GACCGCGAGAAGGGCAAAGGCGCGGGGGCGAGCCGCGUCACCAUCAAGCAGGAGCCCCCC

XM_027129408 GACCGCGAGAAGGGCAAAGGCGCGGGGGCAAGCCGCGUCACCAUCAAGCAGGAGCCCCCC

XM_025879601 GACCGCGAGAAGGGCAAAGGCUCCGGGACGGGCCGCGUCACCAUCAAGCAGGAGCCCCCC

XM_022577811 GACCGCGAGAAGGGCAAAGGCGCGGGGACAAGCCGCGUCACCAUCAAGCAGGAGCCCCCC

XM_019936346 GACCGCGAGAAGGGCAAAGGCGCGGGGGCAAGCCGCGUCACCAUCAAGCAGGAGCCCCCC

XM_027593397 GACCGCGAGAAGGGCAAAGGCUCCGGGACGGGCCGCGUCACCAUCAAGCAGGAGCCCCCC

XM_015093964 GACCGCGAAAAGGGCAAAGGCGCGGGGACGAGCCGCGUUACCAUCAAGCAGGAGCCCCCC

XM_019452268 GACCGCGAGAAGGGCAAAGGCGCCGGGGCGAGCCGCGUCACUAUCAAGCAGGAGCCCCCC

XM_004270956 GACCGCGAGAAGGGCAAAGGCGCGGGGGCAAGCCGCGUCACCAUCAAGCAGGAGCCCCCC

XM_027541573 GACCGCGAGAAGGGCAAAGGCGCGGGGGCGAGCCGCGUCACCAUUAAGCAGGAGCCCCCC

XM_027934162 GACCGCGAGAAGAGCAAAGGCGCAGGGGCGAGCCGCGUCACCAUCAAGCAGGAGCCUCCC

XM_003355541 GACCGCGAGAAAGGCAAAGGCGCGGGGGCGAGCCGCGUCACCAUCAAGCAGGAGCCCCCC

XM_016119294 GACCGCGAGAAGGGCAAAAGCGCGGGGGCGAGCCGCGUCACCAUCAAACAGGAGCCCCCC

XM_006127674 GAUCGAGAGAAGGGCCAAGCCGCGAGGCUGCCCAGCCUGACUAUCAAACAGGAGCCUGCC

NM_030762 GGGGAGGACUCGCCGGCGCCCAAGAGGAUGAAGCUGGAUUCCCGCGGCGGCGGCAGCGGC

XM_520805 GGGGAGGACUCGCCGGCGCCCAAGAGGAUGAAGCUGGAUUCCCGCGGCGGCGGCAGCGGC

XM_019037881 GGGGAGGACUCGCCGGCGCCCAAGAGGAUGAAGCUGGAUUCCC------------GCGGC

XM_002823045 GGGGAGGACUCGCCGGCGCCCAAGAGGAUGAAGCUGGAUUCCCGCGGCGGCGGCAGCGGC

XM_005570417 GGGGAGGACUUGCCGGCGCCCAAGAGGAUGAAGCUGGAUUCCCGCGGCGGCGGCAGCGGC

XM_012093655 GGGGAGGACUUGCCGGCGCCCAAGAGGAUGAAGCUGGAUUCCCGCGGCGGCGGCAGCGGC

XM_015151321 GGGGAGGACUUGCCGGCGCCCAAGAGGAUGAAGCUGGAUUCCCGCGGCGGCGGCAGCGGC

XM_011759130 GGAGAGGACUUGCCGGCGCCCAAGAGGAUGAAGCUGGAUUCCCGCGGCGGCGGCAGCGGC

XM_007967990 GGGGAGGACUUGCCGGCGCCCAAGAGGAUGAAGCUGGAUACCCGCGGCGGCGGCAGCGGC

XM_023209042 GGGGAGGACUUGCCGGCGCCCAAGAGGAUGAAGCUGGAUUCCC------------GCGGC

XM_025402281 GGGGAGGACUUGCCGGCGCCCAAGAGGAUGAAGCUGGAUUCCC------------GCGGC

XM_017507035 GGGGAGGACUCGCCGGCGCCCAAGAGGAUGAAGCUGGAUUCCCGUGGCG------GCGGC

XM_012739537 GGGGAGGACUCGCCGGCGCCCAAGAGGAUGCGGCUGGAUUCCCGCGGCGGGGCGGCGGGC

XM_007446307 GGGGACGACUCGCCGGCGCCCAAGAGGAUGAAGCUGGAUUCCCGCAGCG------GCGGC

XM_024128992 GGGGAGGACUCGCCGGCGCCCAAGAGGAUGAAGCUGGAUUCCCGC---------AGCGGC

XM_027129408 GGGGAGGACUCGCCGGCGCCCAAGAGGAUGAAGCUGGAUUCCCGC---------AGCGGC

XM_025879601 GGGGAGGACUCGCCGGCGCCCAAGAGGAUGAAGCUGGAUUCCCGCGGCG------GCGGC

XM_022577811 GGGGAGGACUCGCCGGCGCCCAAGAGGAUGAAGCUGGAUUCCCGC---------AGCGGA

XM_019936346 GGGGAGGACUCGCCGGCGCCCAAGAGGAUGAAGCUGGAUUCCCGC---------AGCGGC

XM_027593397 GGGGAGGACUCGCCGGCGCCCAAGAGGAUGAAGCUGGAUUCCCGCGGCG------GCGGC

XM_015093964 GGGGAGGACUUGCCGGCGCCCAAGAGGAUGAGGCUGGAUACCCGCGGCGGCG---GCGGC

XM_019452268 GGGGAGGACUCGCCGGCGCCCAAGAGGAUGAAGCUGGAUUCCCGCG---------GCGGC

XM_004270956 GGGGAGGACUCGCCGGCGCCCAAGAGGAUGAAGCUGGAUUCCCGC---------AGCGGC

XM_027541573 GGGGAGGACUCGCCAGCGCCCAAGAGGAUGAGGCUGGAUACCCGCGGCUGCG---GCGGC

XM_027934162 GGGGAGGACUCGCCGGCGCCCAAGAGGAUGAAGCUGGAUUCCCGUG---------GCGGC

XM_003355541 GGGGAGGACUCGCCGGCGCCCAAGAGGAUGAAGCUGGAUUCCCGCGGCG------GCGGC

XM_016119294 GGGGAGGACUCGCCGGCGCCUAAGAGGAUGAGGCUGGAUUCUC---------------GU

XM_006127674 GGGGACGAGGCCCCAGCGCCCAAAAGGCUGAAGCUGGAUUGCACCAGCAGCAGCAGCAGC

NM_030762 GGCGGCCCGGGGGGCGGCGCGGCGGCGGCGGCAGCCGCGCUUCUGGGGCCCGACCCUGCC

XM_520805 GGCGGCCCGGGGGGCGGCGCGGCGGCGGCGGCAGCCGCGCUCCUGGGGCCCGACCCUGCC

XM_019037881 GGCGGCCCGGGGGGCGGCGCGGCGGCGGCGGCAGCCGCGCUCCUGGGGCCCGACCCUGCC

XM_002823045 GGCGGCCCGGGGGGUGGCGCGGCGGCGGCGGCAGCCGCGCUCCUGGGGCCGGACCCUGCC

XM_005570417 GGCGGCCCGGGGGGCGGCGCGGCGGCGGCGGCAGCUGCGCUCCUGGGGCCCGACCCUGCC

XM_012093655 GGCGGCCCGGGGGGCGGCGCGGCGGCGGCGGCAGCUGCGCUCCUGGGGCCCGACCCUGCC

XM_015151321 GGCGGCCCGGGGGGCGGCGCGGCGGCGGCGGCAGCUGCGCUCCUGGGGCCCGACCCUGCU

XM_011759130 GGCGGCCCGGGGGGCGGCGCGGCGGCGGCGGCAGCUGCGCUCCUGGGGCCCGACCCUGCC

XM_007967990 GGCGGCCCGGGGGGCGGCGCGGCGGCGGCGGCAGCUGCGCUCCUGGGGCCCGACCCUGCC

XM_023209042 GGCGGCCCGGGGGGCGGCGCGGCGGCGGCGGCAGCUGCGCUCCUGGGGCCCGACCCUGCC

XM_025402281 GGCGGCCCGGGGGGCGGCGCGGCGGCGGCGGCAGCUGCGCUCCUGGGGCCCGACCCUGCC

XM_017507035 GGCGGCCCGGGGGGCGGCGCGGCGGCGGCGGCAGCCGCGCUCCUGGGGCCGGACCCUGCC

XM_012739537 GGCGGCGGCCCGGGCGGCGCGGCGCGGGCGGCGGCCGCGCUCCUGGGGCCAGACCCGGCC

XM_007446307 GGCGGCCUGGGGGGCGGCGCGGCGGCGGCGGCGGCCGCGCUCCUGGGGCCCGACCCGGCC

XM_024128992 GGCGGCCUGGGGGGCGGCGCGGCGGCGGCGGCGGCCGCGCUCCUGGGGCCCGACCCGGCC

XM_027129408 GGCGGCCUGGGGGGCGGCGCGGCGGCGGCGGCCGCCGCGCUCCUGGGGCCCGACCCGGCC

XM_025879601 GGCGGCCUGGGGGGCGGCGCGGCGGCGGCGGCGGCCGCGCUCCUGGGGCCGGACCCGGCC

XM_022577811 GGCGGCCUGGGGGGCGGCGCGGCGGCGGCGGCCGCCGCGCUCUUGGGGCCCGACCCGGCC

XM_019936346 GGCGGCCUGGGGGGCGGCGCGGCGGCGGCGGCCGCCGCGCUCCUGGGGCCCGACCCGGCC

XM_027593397 GGCGGCCUGGGGGGCGGCGCGGCGGCGGCGGCGGCCGCGCUCCUGGGGCCGGACCCGGCC

XM_015093964 GGCGGCCCGGGGGGCGGCGCGGCGGCGGCGGCGGCCGCGCUCCUGGGGCCCGACCCGGCC

XM_019452268 GGCGGCCUGGGGGGCGGCGCGGCGGCGGCGGCGGCCGCGCUCCUGGGGCCCGACCCGGCC

XM_004270956 GGCGGCCUGGGGGGCGGCGCGGCGGCGGCGGCCGCCGCGCUCCUGGGGCCCGACCCGGCC

XM_027541573 GGCGGCCCGGGGGGCGGCGCGGCGGCGGCGGCGGCCGCGCUCCUGGGGCCCGACCCGACC

XM_027934162 GGCGGCCCGGGGGGCGGCGCGGCGGCGGCGGCGGCCGCACUCCUGGGGCCUGACCCGGCC

XM_003355541 GGCGGUCCGGGGGGUGGCGCGGCGGCGGCGGCAGCCGCGCUCCUGGGGCCGGACCCGGCC

XM_016119294 GGCGGCGCAGGGGGCGGCGCGGCGGCGGCUGCCGCCGCGCUUCUGGGGCCAGACCCCGCC

XM_006127674 AGCAAUAGCCACAGCGCCCCCCUCCCUUCCGCCGCCGCGCUCAGCCCGGAC------CCG

NM_030762 GCCGCGGCCGCGCUGCUGAGACCCGACGCCGCCCUGCUCAGCUCGCUGGUGGCGUUCGGC

XM_520805 GCCGCGGCCGCGCUGCUGAGACCCGACGCCGCCCUGCUCAGCUCGCUGGUGGCGUUCGGC

XM_019037881 GCCGCGGCCGCGCUGCUGAGACCCGACGCCGCCCUGCUCAGCUCGCUGGUUGCGUUCGGC

XM_002823045 GCCGCGGCCGCGCUGCUGAGACCCGACGCCGCCCUGCUCAGCUCGCUGGUGGCGUUCGGC

XM_005570417 GCCGCGGCCGCGCUGCUGAGACCCGACGCCGCCCUGCUCAGCUCGCUGGUGGCGUUCGGC

XM_012093655 GCCGCGGCCGCGCUGCUGAGACCCGACGCCGCCCUGCUCAGCUCGCUGGUGGCGUUCGGC

XM_015151321 GCCGCGGCCGCGCUGCUGAGACCCGACGCCGCCCUGCUCAGCUCGCUGGUGGCGUUCGGC

XM_011759130 GCCGCGGCCGCGCUGCUGAGACCCGACGCCGCCCUGCUCAGCUCGCUGGUGGCGUUCGGC

XM_007967990 GCCGCGGCCGCGCUGCUGAGACCCGACGCCGCCCUGCUCAGCUCGCUGGUGGCGUUCGGC

XM_023209042 GCCGCGGCCGCGUUGCUGAGACCCGACGCCGCCCUGCUCAGCUCGCUGGUGGCGUUCGGC

XM_025402281 GCCGCGGCCGCGCUGCUGAGACCCGACGCCGCCCUGCUCAGCUCGCUGGUGGCGUUCGGC

XM_017507035 GCCGCGGCUGCGCUGCUGAGACCCGACGCCGCCCUGCUCAGCUCGCUGGUGGCGUUCGGC

XM_012739537 GCGGCCGCCGCGCUGCUGAGACCCGACGCCGCCCUGCUCAGCUCGCUGGUGGCGUUCGGC

XM_007446307 ACCCCGGCCGCGCUGCUGAGACCCGACGCUGCCCUGCUCAGCUCGCUGGUGGCGUUCGGC

XM_024128992 GCCCCGGCCGCGCUGCUGAGACCCGACGCUGCCCUGCUCAGCUCGCUGGUGGCGUUCGGC

XM_027129408 GCCCCGGCCGCGCUGCUGAGACCCGACGCUGCCCUGCUCAGCUCGCUGGUGGCGUUCGGC

XM_025879601 GCCGCGGCAGCGCUGCUGAGACCCGACGCGGCCCUGCUCAGCUCGCUGGUGGCGUUCGGC

XM_022577811 GCCCCGGCCGCGCUGCUGAGACCCGACGCUGCCCUGCUCAGCUCGCUGGUGGCGUUCGGC

XM_019936346 GCCCCGGCCGCGCUGCUGAGACCCGACGCUGCCCUGCUCAGCUCGCUGGUGGCGUUCGGC

XM_027593397 GCCGCGGCAGCGCUGCUGAGACCCGACGCGGCCCUGCUCAGCUCGCUGGUGGCGUUCGGC

XM_015093964 GCCGCGGCCGCGCUGCUGAGACCCGACGCCGCCCUGCUCAGCUCGCUGGUGGCGUUCGGC

XM_019452268 GCCGCGGCAGCGCUGCUGAGACCCGACGCCGCCCUGCUCAGCUCGCUGGUGGCUUUCGGC

XM_004270956 GCCCCGGCCGCGCUGCUGAGACCCGACGCUGCCCUGCUCAGCUCUCUGGUGGCGUUCGGC

XM_027541573 GCUGCGGCCGCGCUGCUGAGACCCGACGCCGCCCUGCUCAGCUCGCUGGUGGCGUUCGGC

XM_027934162 GCUGCGGCCGCGCUGCUGAGACCCGACGCCGCCCUGCUCAGCUCGCUAGUGGCGUUCGGC

XM_003355541 GCUGCAGCCGCACUGCUAAGACCCGACGCCGCCCUGCUCAGCUCGCUGGUGGCGUUCGGC

XM_016119294 GCCGCGGCCGCGCUGCUGAGACCCGACGCCGCGCUGCUCAGUUCGCUGGUGGCGUUCGGC

XM_006127674 GCCGCGGCUGCCCUGCUGAGACCCGACGCCGCCCUGCUCAGCUCCCUCCUGGCCUUUGGA

NM_030762 GGAGGCGGAGGCGCGCCCUUCCCGCAGCCCGCGGCCGCCG---------CGGCCCCCUUC

XM_520805 GGAGGCGGGGGCGCGCCCUUCCCGCAGCCCGCGGCCGCCG---------CGGCCCCCUUC

XM_019037881 GGAGGCGGGGGCGCGCCCUUCCCGCAGCCCGCGGCCGCC---------------------

XM_002823045 GGAGGCGGGGGCGCGCCCUUCCCGCAGCCCGCGGCCGCCG---------CGGCCCCCUUC

XM_005570417 GGAGGCGGGGGCGCGCCCUUCCCGCAGCCCGCGGCCGCCG---------CGGCCCCCUUC

XM_012093655 GGAGGCGGGGGCGCGCCCUUCCCGCAGCCCGCGGCCGCCG---------CGGCCCCCUUC

XM_015151321 GGAGGCGGGGGCGCGCCCUUCCCGCAGCCCGCGGCCGCCG---------CGGCCCCCUUC

XM_011759130 GGAGGCGGGGGCGCGCCCUUCCCGCAGCCCGCGGCCGCCG---------CGGCCCCCUUC

XM_007967990 GGAGGCGGGGGCGCGCCCUUCCCGCAGCCCGCGGCCGCCG---------CGGCCCCCUUC

XM_023209042 GGAGGCGGGGGCGCGCCCUUCCCGCAGCCCGCGGCCGCCG---------CGGCCCCCUUC

XM_025402281 GGAGGCGGGGGCGCGCCCUUCCCGCAGCCCGCGGCCGCAG---------CGGCCCCCUUC

XM_017507035 GGAGGCGGGGGCGCGCCCUUCCCGCAGCCCGCAGCCGCCG---------CGGCCCCCUUC

XM_012739537 GGCGGCGGGGGCGCGCCCUUCGCGCAGCCCGCGGCCGCCG---------CGGCCCCCUUC

XM_007446307 GGAGGCGGGGGCGCGCCCUUCGCGCAGCCUGCGGCCGCCG---------CGGCCCCCUUC

XM_024128992 GGAGGCGGGGGCGCGCCCUUCGCGCAGCCUGCGGCCGCCG---------CGGCCCCCUUC

XM_027129408 GGAGGCGGGGGCGCGCCCUUCGCGCAGCCUGCGGCCGCCG---------CGGCCCCCUUC

XM_025879601 GGAGGCGGGGGCGCGCCCUUCGCGCAGCCCGCCGCCGCCGCGGCUGCCGCGGCCCCCUUC

XM_022577811 GGAGGCGGGGGCGCGCCCUUCGCGCAGCCUGCGGCCGCCG---------CGGCCCCCUUC

XM_019936346 GGAGGCGGGGGCGCGCCCUUCGCGCAGCCUGCGGCCGCCG---------CGGCCCCCUUC

XM_027593397 GGAGGCGGGGGCGCGCCCUUCGCGCAGCCCGCCGCCGCCGCGGCUGCCGCGGCCCCCUUC

XM_015093964 GGAGGCGGGGGCGCGCCCUUCGCGCAGCCCGCGGCCGCCG---------CGGCCCCCUUC

XM_019452268 GGAGGCGGGGGCGCGCCCUUCGCGCAGCCCGCGGCCGCCGCGGCCGCCGCGGCCCCCUUC

XM_004270956 GGAGGCGGGGGCGCGCCCUUCGCGCAGCCUGCGGCCGCCG---------CGGCCCCCUUC

XM_027541573 GGAGGCGGGGGCGCGCCCUUCGCGCAGCCGGCGGCCGCCG---------CGGCCCCUUUC

XM_027934162 GGAGGCGGGGGUGCACCCUUCGCUCAGCCCGCCGCAG------------CGGCCCCCUUC

XM_003355541 GGAGGCGGGGGCGCGCCCUUCGCGCAGCCGGCGGCUGCUG---------CAGCCCCCUUC

XM_016119294 GGAGGCGGGGGCGCGCCCUUCGCGCAGCCUGCGGCCGCCG---------CGGCCCCCUUC

XM_006127674 GGAGGCGGGGCCGGGGCUCCCUUUGGCCAGCAAGCCG------------CGGCCCCGCUU

NM_030762 UGCCUGCCCUUCUGCUUCCUCUCGCCUUCUGCAGCUGCCGCCUACGUGCAGCCCUUCCUG

XM_520805 UGCCUGCCCUUCUGCUUCCUCUCGCCUUCUGCAGCUGCCGCCUACGUGCAGCCCUUCCUG

XM_019037881 ------------------------------------------------------------

XM_002823045 UGCCUGCCCUUCUGCUUCCUCUCGCCUUCUGCAGCUGCCGCCUACGUGCAGCCCUUCCUG

XM_005570417 UGCCUGCCCUUCUGCUUCCUCUCGCCUUCUGCAGCCGCCGCCUACGUGCAGCCCUUCCUG

XM_012093655 UGCCUGCCCUUCUGCUUCCUCUCGCCUUCUGCAGCCGCCGCCUACGUGCAGCCCUUCCUG

XM_015151321 UGCCUGCCCUUCUGCUUCCUCUCGCCUUCUGCAGCCGCCGCCUACGUGCAGCCCUUCCUG

XM_011759130 UGCCUGCCCUUCUGCUUCCUCUCGCCUUCUGCAGCCGCCGCCUACGUGCAGCCCUUCCUG

XM_007967990 UGCCUGCCCUUCUGCUUCCUCUCGCCUUCUGCAGCCGCCGCCUACGUGCAGCCCUUCCUG

XM_023209042 UGCCUGCCCUUCUGCUUCCUCUCGCCUUCUGCAGCCGCCGCCUACGUGCAGCCCUUCCUG

XM_025402281 UGCCUGCCCUUCUGCUUCCUCUCGCCUUCUGCAGCCGCCGCCUACGUGCAGCCCUUCCUG

XM_017507035 UGCCUGCCCUUCUACUUCCUCUCGCCUUCUGCAGCCGCCGCCUACGUGCAGCCCUUCCUG

XM_012739537 UGCCUGCCCUUCUACUUCCUCUCGCCCUCGGCCGCCGCCGCCUACGUGCAGCCCUUCCUG

XM_007446307 UGCCUGCCCUUCUACUUCCUCUCGCCUUCGGCGGCCGCCGCCUACGUGCAGCCCUUCCUG

XM_024128992 UGCCUGCCCUUCUACUUCCUCUCGCCUUCGGCGGCCGCCGCCUACGUGCAGCCCUUCCUG

XM_027129408 UGCCUGCCCUUCUAUUUCCUCUCGCCUUCGGCGGCCGCCGCCUACGUGCAGCCCUUCCUG

XM_025879601 UGCCUGCCCUUCUACUUCCUCUCGCCUUCGGCGGCCGCCGCCUACGUGCAGCCUUUCCUG

XM_022577811 UGCCUGCCCUUCUACUUCCUCUCGCCUUCGGCGGCCGCCGCCUACGUGCAGCCCUUCCUG

XM_019936346 UGCCUGCCCUUCUAUUUCCUCUCGCCUUCGGCGGCCGCCGCCUACGUGCAGCCCUUCCUG

XM_027593397 UGCCUGCCCUUCUACUUCCUCUCGCCUUCGGCGGCCGCCGCCUACGUGCAGCCUUUCCUG

XM_015093964 UGCCUGCCCUUCUACUUCCUCUCGCCUUCGGCGGCCGCCGCCUACGUGCAGCCCUUCCUG

XM_019452268 UGCCUGCCCUUCUACUUCCUCUCGCCUUCGGCGGCCGCCGCCUACGUGCAGCCCUUCCUG

XM_004270956 UGCCUGCCCUUCUAUUUCUUCUCGCCUUCGGCGGCCGCCGCCUACGUGCAGCCCUUCCUG

XM_027541573 UGCCUGCCCUUCUACUUCCUCUCGCCUUCGGCGGCCGCCGCCUACGUGCAGCCCUUCCUG

XM_027934162 UGCCUGCCUUUCUACUUCCUCUCGCCUUCCGCGGCCGCCGCCUACGUGCAGCCUUUCCUG

XM_003355541 UGCCUGCCCUUCUACUUCCUCUCGCCUUCCGCGGCCGCCGCCUACGUGCAGCCCUUCCUG

XM_016119294 UGCCUGCCCUUCUACUUCCUCUCGCCUUCAGCAGCCGCCGCCUACGUGCAGCCCUUCCUG

XM_006127674 UGCCUGCCCUUCUACUUCCUCUCCCCCUCCGCGGCCGCCGCCUACAUGCAGCCCUUGCUG

NM_030762 GACAAGAGCGGCCUGGAGAAGUAUCUGUACCCGGCGGCGGCUGCCGCCCCGUUCCCGCUG

XM_520805 GACAAGAGCGGCCUGGAGAAGUAUCUGUACCCGGCGGCGGCUGCCGCCCCGUUCCCGCUG

XM_019037881 ------------------------------------------------------------

XM_002823045 GACAAGAGCGGCCUGGAGAAGUAUCUGUACCCGGCGGCGGCCGCCGCCCCGUUCCCGCUG

XM_005570417 GACAAGAGCGGCCUGGAGAAGUAUCUGUACCCGGCGGCGGCCGCCGCCCCGUUCCCACUG

XM_012093655 GACAAGAGCGGCCUGGAGAAGUAUCUGUACCCGGCGGCGGCCGCCGCCCCGUUCCCACUG

XM_015151321 GACAAGAGCGGCCUGGAGAAGUAUCUGUACCCGGCGGCGGCCGCCGCCCCGUUCCCACUG

XM_011759130 GACAAGAGCGGCCUGGAGAAGUAUCUGUACCCGGCGGCGGCCGCCGCCCCGUUCCCACUG

XM_007967990 GACAAGAGCAGCCUGGAGAAGUAUCUGUACCCGGCGGCGGCCGCCGCCCCGUUCCCACUG

XM_023209042 GACAAGAGCGGCCUGGAGAAGUAUCUGUACCCGGCGGCGGCUGCCACCCCGUUCCCACUG

XM_025402281 GACAAGAGCGGCCUGGAGAAGUAUCUGUACCCGGCGGCGGCCGCCGCCCCGUUCCCACUG

XM_017507035 GACAAGAGCGGCCUGGAGAAGUAUCUGUACCCGGCGGCGGCCGCCGCCCCGUUCCCGCUG

XM_012739537 GACAAGAGCGGCCUGGAGAAGUAUCUGUACCCGGCGGCCGCCGCCGCCCCGUUCCCGCUG

XM_007446307 GACAAGAGCGGCCUGGAGAAGUACCUGUACCCGGCGGCGGCCGCCGCCCCGUUCCCACUA

XM_024128992 GACAAGAGCGGCCUGGAGAAGUACCUGUACCCGGCGGCGGCCGCCGCCCCGUUCCCACUG

XM_027129408 GACAAGAGCGGCCUGGAGAAGUACCUGUACCCGGCGGCGGCCGCCGCCCCGUUCCCACUA

XM_025879601 GACAAGAGCGGCCUGGAGAAGUACCUGUACCCGGCGGCGGCCGCCGCCCCGUUCCCACUG

XM_022577811 GACAAGAGCGGCCUGGAGAAGUACCUGUACCCGGCGGCGGCCGCCGCCCCGUUCCCACUA

XM_019936346 GACAAGAGCGGCCUGGAGAAGUACCUGUACCCGGCGGCGGCCGCCGCCCCGUUCCCACUA

XM_027593397 GACAAGAGCGGCCUGGAGAAGUACCUGUACCCGGCGGCGGCCGCCGCCCCGUUCCCACUG

XM_015093964 GACAAGAGCGGCCUGGAGAAGUAUCUGUACCCGGCGGCGGCCGCCGCCCCGUUCCCAUUG

XM_019452268 GACAAGAGCGGCCUGGAGAAGUACCUGUAUCCGGCGGCGGCCGCCGCCCCGUUCCCACUA

XM_004270956 GACAAGAGCGGCCUGGAGAAGUACCUGUACCCGGCGGCGGCCGCCGCCCCGUUCCCACUA

XM_027541573 GACAAGAGCGGCCUGGAGAAGUACCUGUACCCGGCGGCGGCCGCCGCCCCGUUUCCAUUG

XM_027934162 GACAAGAGCGGCCUGGAGAAGUAUCUGUACCCCACGGCGGCUGCCGCCCCGUUCCCACUG

XM_003355541 GACAAGAGCGGCCUGGAGAAGUACCUGUACCCGGCGGCGGCCGCCGCCCCAUUCCCACUG

XM_016119294 GACAAGAGCAGCCUGGAGAAGUACCUGUACCCGGCGGCGGCCGCCGCCCCCUUCCCGCUG

XM_006127674 GACAAGAGCAACCUGGAGAAAUAUCUCUACCC------GGCCGCCGCCCCCAUCCCUUUG

NM_030762 CUAUACCCCGGCAUCCCCGCCCCGGCGGCAGCCGCGGCAGCCGCCGCCGCCGCUGCCGCC

XM_520805 CUAUACCCCGGCAUCCCCGCCCCGGCGGCAGCCGCGGCAGCCGCCGCCGCCGCUGCCGCC

XM_019037881 ------------------------------------------------------GCGGCC

XM_002823045 CUAUACCCCGGCAUCCCCGCCCCGGCGGCAGCCGCGGCCGCCGCCGCCGCCGCUGCCGCC

XM_005570417 CUAUACCCCGGCAUCCCUGCCCCGGCCGCAGCCGCGGCCGCCGCCGCCGCCGCUGCCGCC

XM_012093655 CUAUACCCCGGCAUCCCUGCCCCGGCCGCAGCCGCGGCCGCCGCCGCCGCCGCUGCCGCC

XM_015151321 CUAUACCCCGGCAUCCCUGCCCCGGCCGCAGCCGCGGCCGCCGCCGCCGCCGCUGCCGCC

XM_011759130 CUAUACCCCGGCAUCCCUGCCCCGGCCGCAGCCGCGGCCGCCGCCGCCGCCGCUGCCGCC

XM_007967990 CUAUACCCCGGCAUCCCUGCUCCGGCCGCAGCCGCGGCCGCCGCCGCCGCCGCUGCCGCC

XM_023209042 CUAUACCCCGGCAUCCCUGCCCCGGCCGCAGCCGCGGCCGCCGCUGCAGCCGCUGCCGCC

XM_025402281 CUAUACCCCGGCAUCCCUGCCCCGGCCGCAGCCGCGGCCGCUGCCGCCGCCGCUGCCGCC

XM_017507035 CUGUACCCUGGCAUCCCCGCCCCGGCC---GCGGCCGCCGCCGCCGCAGCUGCUGCCGCC

XM_012739537 CUGUACCCCGGCAUCCCCGCCCCGGCC---GCCGCCGCCGCCGCCGCCGCGGCCGCCGCC

XM_007446307 CUGUACCCCGGCAUCCCCGCCCCGGCC---GCCGCCGCCGCCGCCGCCGCUGCCGCCGCC

XM_024128992 CUGUACCCCGGCAUCCCCGCCCCGGCC---GCCGCCGCCGCCGCCGCCGCUGCCGCCGCC

XM_027129408 CUGUACCCCGGCAUCCCCGCCCCGGCC---GCCGCCGCCGCCGCCGCCGCUGCCGCCGCC

XM_025879601 CUGUACCCCGGCAUCCCCGCGCCGGC---AGCCGCCGCCGCAGCCGCCGCCGCAGCCGCC

XM_022577811 CUGUACCCCGGCAUCCCCGCCCCGGCCGCCGCCGCCGCCGCCGCCGCCGCCGCCGCCGCC

XM_019936346 CUGUACCCCGGCAUCCCCGCCCCGGCC---GCCGCCGCCGCCGCCGCCGCUGCCGCAGCC

XM_027593397 CUGUACCCCGGCAUCCCCGCGCCGGC---AGCCGCCGCCGCAGCCGCCGCCGCAGCCGCC

XM_015093964 CUGUACCCCGGCAUCCCCGCUCCGGCC---GCCGCCGCCGCCGCCGCCGCGGCCGCCGCC

XM_019452268 CUGUACCCCGGCAUCCCCGCGCCAGC---AGCCGCCGCCGCCGCCGCCGCCGCAGCCGCC

XM_004270956 CUGUACCCCGGCAUCCCCGCCCCGG------CCGCCGCCGCCGCCGCCGCCGCCGCUGCC

XM_027541573 CUGUACCCCGGCAUCCCUGCUCCGGCC---GCCGCCGCCGCCGCCGCCGCGGCCGCGGCC

XM_027934162 UUGUACCCCGGCAUCCCCGCCCCAGC---UGCCGCCGCCGCUGCUGCUGCAGCGGCCGCC

XM_003355541 CUAUACCCCGGAAUCCCCGCCCCGGCC---GCCGCCGCCGCAGCUGCAGCCGCCGCAGCC

XM_016119294 UUGUACCCUGGCAUCCCCGCCCCAGC---UGCCGCAGCCGCAGCCGCUGCCGCCGCCGCU

XM_006127674 CUCUACCCCGGAAUCCCCGCCCAG------------------------------------

NM_030762 GCCGCCGCCGCGUUCCCCUGCCUGUCCUCGGUGUUGUCGCCCCCUCCCGAGAAGGCG---

XM_520805 GCCGCCGCCGCGUUCCCCUGCCUGUCCUCGGUGUUGUCGCCCCCUCCCGAGAAGGCG---

XM_019037881 GCCGCCGCCGCGUUCCCCUGCCUGUCCUCGGUGUUGUCGCCCCCUCCCGAGAAGGCG---

XM_002823045 GCCGCCGCCGCGUUCCCCUGCCUGUCCUCGGUGUUGUCGCCCCCUCCCGAGAAGGCG---

XM_005570417 GCCGCCGCCGCCUUCCCCUGCCUGUCCUCGGUGUUGUCGCCCCCUCCCGAGAAGGCG---

XM_012093655 GCCGCCGCCGCCUUCCCCUGCCUGUCCUCGGUGUUGUCGCCCCCGCCCGAGAAGGCG---

XM_015151321 GCCGCCGCCGCCUUCCCCUGCCUGUCCUCGGUGUUGUCGCCCCCUCCCGAGAAGGCG---

XM_011759130 GCCGCCGCCGCCUUCCCCUGCCUGUCCUCGGUGUUGUCGCCCCCUCCCGAGAAGGCG---

XM_007967990 GCCGCCGCCGCCUUCCCCUGCCUGUCCUCGGUGUUGUCGCCCCCUCCCGAGAAGGCG---

XM_023209042 GCCGCCGCCGCCUUCCCCUGUCUGUCCUCGGUGUUGUCGCCCCCUCCCGAGAAGGCG---

XM_025402281 GCUGCCGCCGCCUUCCCCUGCCUGUCCUCGGUGUUGUCGCCCCCGCCCGAGAAGGCG---

XM_017507035 GCCGCCGCCGCCUUCCCCUGCCUGUCCUCGGUGUUGUCGCCCCCUCCGGAGAAGGCG---

XM_012739537 GCCGCCGCCGCCUUCCCCUGCCUGUCCUCCGUGUUGUCGCCCCCUCCCGAGAAAGCCGC-

XM_007446307 GCCGCCGCCGCCUUCCCCUGCCUGUCCUCCGUGUUGUCGCCCCCUCCCGAGAAGGCGGCA

XM_024128992 GCCGCCGCCGCCUUCCCCUGCCUGUCCUCCGUGUUGUCGCCCCCUCCCGAGAAGGCGGCA

XM_027129408 GCCGCCGCCGCCUUCCCCUGCCUGUCCUCCGUGUUGUCGCCCCCUCCCGAGAAGGCGGCA

XM_025879601 GCCGCCGCUGCCUUCCCCUGCCUGUCCUCGGUGUUGUCGCCCCCUCCCGAGAAGGC----

XM_022577811 GCCGCCGCCGCCUUCCCCUGCCUGUCCUCCGUGUUGUCGCCCCCUCCCGAGAAGGCGGCA

XM_019936346 GCCGCCGCCGCCUUCCCCUGCCUGUCCUCCGUGUUGUCGCCCCCUCCCGAGAAGGCGGCA

XM_027593397 GCCGCCGCUGCCUUCCCCUGCCUGUCCUCGGUGUUGUCGCCCCCUCCCGAGAAGGC----

XM_015093964 GCCGCCGCCGCCUUCCCCUGCCUGUCCUCUGUGUUGUCGCCCCCUCCCGAGAAGGCGGCG

XM_019452268 GCCGCCGCCGCCUUCCCCUGCCUGUCCUCGGUGUUGUCGCCCCCUCCCGAGAAGGC----

XM_004270956 GCCGCCGCCGCCUUCCCCUGCCUGUCCUCCGUGUUGUCGCCCCCUCCCGAGAAGGCGGCA

XM_027541573 GCCGCCGCCGCCUUCCCCUGCCUGUCCUCCGUGUUGUCGCCCCCUCCCGAGAAGGCGGCG

XM_027934162 GCGGCCGCCGCCUUCCCCUGUCUGUCCUCGGUGUUGUCGCCCCCUCCCGAGAAGGCGGG-

XM_003355541 GCCGCAGCCGCCUUCCCCUGCCUGUCCUCAGUGUUGUCGCCCCCUCCGGAAAAGGC---A

XM_016119294 GCUGCCGCCGCCUUUCCCUGCCUGUCCUCGGUGUUGUCGCCCCCUCCCGAGAAGGUGGG-

XM_006127674 GCCGCCGCUGCCUUCCCCUGCCUCUCCUCGGUGCUGGCGUCAGCU---GAGAAGGCGAAC

NM_030762 ---GGCGCCGCCGCCGCGACCCUCCUGCCGCACGAGGUGGCGCCCCUUGGGGCGCCGCAC

XM_520805 ---GGCGCCGCCGCCGCGACCCUCCUGCCGCACGAGGUGGCGCCCCUUGGGGCGCCGCAC

XM_019037881 ---GGCGCCGCCGCCGCGACCCUCCUGCCGCACGAGGUGGCGCCCCUUGGGGCGCCGCAC

XM_002823045 ---GGCGCCGCCGCCGCGACCCUCCUGCCGCACGAGGUGGCGCCCCUUGGGGCGCCGCAC

XM_005570417 ---GGCGCCGCCGCCGCGACCCUCCUGCCGCACGAGGUGGCGCCCCUUGGGGCGCCGCAC

XM_012093655 ---GGCGCCGCCGCCGCGACCCUCCUGCCGCACGAGGUGGCGCCCCUUGGGGCGCCGCAC

XM_015151321 ---GGCGCCGCCGCCGCGACCCUCCUGCCGCACGAGGUGGCGCCCCUUGGGGCGCCGCAC

XM_011759130 ---GGCGCCGCCGCCGCGACCCUCCUGCCGCACGAGGUGGCGCCCCUUGGGGCGCCGCAC

XM_007967990 ---GGCGCCGCCGCCGCGACCCUCCUGCCGCACGAGGUGGCGCCCCUUGGGGCGCCGCAC

XM_023209042 ---GGCGCCGCCGCCGCGACCCUCCUGCCGCACGAGGUGGCGCCCCUUGGGGCGCCGCAC

XM_025402281 ---GGCGCCGCCGCCGCGACCCUCUUGCCGCACGAGGUGGCGCCCCUUGGGGCGCCGCAC

XM_017507035 ---GGUGCCGCCGCCGCGACCCUCCUGCCGCACGAGGUGGCGCCCCCUGGGGCACCGCAC

XM_012739537 --GGGCGCCGCCGCCGCGACCCUCCUGCCGCACGAGGUGGCGCCCCCUGCGGCGCUGCAC

XM_007446307 ---GCCGCCGCCGCCGCGACCCUCCUGCCGCACGAGGUGGCGCCCCCUGGGGCGCUGCAC

XM_024128992 GCCGCCGCCGCCGCCGCGACCCUCCUGCCGCACGAGGUGGCGCCCCCUGGGGCGCUGCGC

XM_027129408 ---GCCGCCGCUGCCGCGACCCUCCUGCCGCACGAGGUGGCGCCCCCUGGGGCGCUGCAC

XM_025879601 --CAGCGCCGCCGCCGCGACCCUCUUGCCGCACGAGGUGGCGCCCCCUGGGGCGCUGCAC

XM_022577811 ---GCCGCCGCUGCCGCGACCCUCCUGCCGCACGAGGUGGCGCCCCCUGGGGCGCUGCAC

XM_019936346 ---GCCGCCGCUGCCGCGACCCUCCUGCCGCACGAGGUGGCGCCCCCUGGGGCGCUGCAC

XM_027593397 --CAGCGCCGCCGCCGCGACCCUCUUGCCGCACGAGGUGGCGCCCCCUGGGGCGCUGCAC

XM_015093964 GCCGCCGCCGCAGCCGCGACCCUCCUGCCGCACGAGGUGGCGCCCCCUGGGGCUCUGCAC

XM_019452268 --CGGCGCCGCCGCCGCGACCCUCCUGCCGCACGAGGUGGCGCCCCCUGGGGCGCUGCAC

XM_004270956 ---GCCGCCGCUGCCGCGACCCUCCUGCCGCACGAGGUGGCGCCCCCUGGGGCGCUGCAC

XM_027541573 GCCGCCGCCGCAGCCGCGACCCUCCUGCCACACGAGGUGGCGCCCCCUGGGGCUCUGCAC

XM_027934162 --CGCGGCUGCUGCCGCGACCCUCUUGUCGCACGAGGUGGCGCCCCCUGGGGCGUUGCAU

XM_003355541 GCGGCCGCCGCCGCCGCGACCCUCCUGCCGCACGAGGUGGCGUCCCCCGGAGCGCUGCAC

XM_016119294 --CGCCGCAGCCGCCGCGACCCUCCUGCCGCACGAGGUGGCGCCCCCUGGGUCACUGCAU

XM_006127674 GCGGCAGCCGCCGCCUCGGCCCUCCUGCCUCUCGACGUGGUCUCGCCU------------

NM_030762 CCCCAGCACCCGCACGGCCGCACCCACCUGCCCUUCGCCGGGCCCCGCGAGCCGGGGAAC

XM_520805 CCCCAGCACCCGCACGGCCGCACCCACCUGCCCUUCGCCGGGCCCCGCGAGCCGGGGAAC

XM_019037881 CCCCAGCACCCGCACGGCCGCACCCACCUGCCCUUCGCCGGGCCCCGCGAGCCGGGGAAC

XM_002823045 CCCCAGCACCCGCACGGCCGCACCCACCUGCCCUUCGCCGGCCCACGCGAGCCGGGGAAC

XM_005570417 CCACAACACCCGCACGGCCGCACCCACCUGCCCUUCGCCGGCCCCCGCGAGCCGGGGAAC

XM_012093655 CCACAACACCCGCACGGCCGCACCCACCUGCCCUUCGCCGGCCCCCGCGAGCCGGGGAAC

XM_015151321 CCACAACACCCGCACGGCCGCACCCACCUGCCCUUCGCCGGCCCCCGCGAGUCGGGGAAC

XM_011759130 CCACAACACCCGCACGGCCGCACCCACCUGCCCUUCGCCGGCCCCCGCGAGCCGGGGAAC

XM_007967990 CCACAACACCCGCACGGCCGCACCCACCUGCCCUUCGCCGGCCCCCGCGAGCCGGGGAAC

XM_023209042 CCACAGCACCCGCACGGCCGCACCCACCUGCCCUUCGCCGGCCCCCGCGAGCCGGGGAAC

XM_025402281 CCACAACACCCGCACGGCCGCACCCACCUGCCCUUCGCCGGCCCCCGCGAGCCGGGGAAC

XM_017507035 CCCCCGCACCCGCACGGCCGCACCCACCUGCCCUUCGCCGGCCCCCGCGAGCCGGGGAAC

XM_012739537 GCCCCGCACCCGCACGGCCGCACCCACCUGCCCUUCGCCGGCCCCCGCGAGCCGGGGAAC

XM_007446307 CCCGCGCCCCCGCACGGCCGCACCCACCUGCCCUUCGCCGUCCGCAGCGAGCCGGGGAAC

XM_024128992 CCCCCGCCCCCGCACGGCCGCACCCACCUGCCCUUCGCCGGCCGCAGCGAGCCGGGGAAC

XM_027129408 CCCCCGCCCCCGCACGGCCGCACCCACCUGCCCUUCGCCGGCCGCAGCGAGCCGGGGAAC

XM_025879601 CCCCCGCACCCGCACGGCCGCACCCACCUGUCCUUCGCCGGCGCCCGCGAGCCCGGGAAC

XM_022577811 CCCCCGCCCCCGCACGGCCGCACCCACCUGCCCUUCGCCGGCCGCAGCGAGCCGGGGAAC

XM_019936346 CCCCCGCCCCCGCACGGCCGCACCCACCUGCCCUUCGCCGGCCGCAGCGAGCCGGGGAAC

XM_027593397 CCCCCGCACCCGCACGGCCGCACCCACCUGUCCUUCGCCGGCGCCCGCGAGCCCGGGAAC

XM_015093964 CCCGCGCACCCGCACGGCCGCACCCACCUGCCCUUCGCCGGCGCUCGCGAGCCCGGGAAC

XM_019452268 CCCCCGCACCCGCACGGCCGCACCCACCUGUCCUUCGCUGGCGCCCGUGAGCCCGGGAAC

XM_004270956 CCCCCGCCCCCGCACGGCCGCACCCACCUGCCCUUCGCCGGCCGCAGCGAGCCGGGGAAC

XM_027541573 CCCGCGCACCCGCACGGCCGCACCCACCUGCCCUUCGCCGGCGCUCGCGAGCCCGGGAAC

XM_027934162 CCCCCGCAUCCGCACGGCCGCACCCACCUGCCCUUCGCCGGCCCCCGCGAGCCGGGGAAC

XM_003355541 CCCCCGCAUCCGCACGGCCGCACCCACCUGCCCUUCGCCGGCCCCCGUGAGCCCGGGAAC

XM_016119294 CCCCCUCACCCGCACGGGCGCACCCACCUGCCCUUUGCCGGCCCCCGCGAGCACGGAAAC

XM_006127674 UCCCCGCACCUGCCCCAUCCCUUCGCCGCUGCUUGCGAGACAGGCACCAGCGUGGGGGAC

NM_030762 CCGGAGAGCUCUGCUCAGGAAGAUCCCUCGCAGCCAGGAAAGGAAGCUCCCUGA

XM_520805 CCGGAGAGCUCUGCUCAGGAAGAUCCCUCGCAGCCAGGAAAGGAAGCUCCCUGA

XM_019037881 CCGGAGAGCUCUGCUCAGGAAGAUCCCUCGCAGCCAGGAAAGGAAGCUCCCUGA

XM_002823045 UCGGAGAGCUCUGCUCAGGAAGAUCCCUCGCAGCCAGGAAAGGAAGCUCCCUGA

XM_005570417 CCGGAGAGCUCUGCUCAGGAAGAUCCCUCGCAGCCAGGAAAGGAAGCACCCUGA

XM_012093655 CCGGAGAGCUCUGCUCAGGAAGAUCCCUCGCAGCCAGGAAAGGAAGCACCCUGA

XM_015151321 CCGGAGAGCUCUGCUCAGGAAGAUCCCUCGCAGCCAGGAAAGGAAGCACCCUGA

XM_011759130 CCGGAGAGCUCUGCUCAGGAAGAUCCCUCGCAGCCAGGAAAGGAAGCACCCUGA

XM_007967990 CCGGAGAGCUCUGCUCAGGAAGAUCCCUCGCAGCCAGGAAAGGAAGCACCCUGA

XM_023209042 CCGGAGAGCUCUGCUCAGGAAGAUCCCUCGCAGCCAGGAAAGGAAGCCCCCUGA

XM_025402281 CCGGAGAGCUCUGCUCAGGAAGAUCCCUCGCAGCCAGGAAAGGAAGCACCCUGA

XM_017507035 CCGGAGAGCUCUGCUCAGGAAGAUCCCUCGCAGCCAGGAAAGGAAGCCCCCUGA

XM_012739537 CCGGAGAGCUCCGCUCAGGAAGACCCCUCGCAGCCGGGAAAGGAAGGCCCCUGA

XM_007446307 CCGGAGAGCUCCGCUCAGGAAGAUCCCUCGCAGCCAGGAAAGGAAACCCCCUGA

XM_024128992 CCGGAGAGCUCCGCUCAGGAAGAUCCCUCGCAGCCAGGAAAGGAAACCCCCUGA

XM_027129408 CCGGAGAGCUCCGCUCGGGAAGAUCCCUCGCAGCCAGGAAAGGAAACCCCCUGA

XM_025879601 CCGGAGAGCUCUGCUCAGGAAGAUCCCUCGCAGCCAGGAAAGGAAACCCACUGA

XM_022577811 CCGGAGAGCUCCUCUCAGGAAGAUCCGUCGCAGCCAGGAAAGGAAACCCCCUGA

XM_019936346 CCGGAGAGCUCCGCUCGGGAAGAUCCCUCGCAGCCAGGAAAGGAAACCCCCUGA

XM_027593397 CCGGAGAGCUCUGCUCAGGAAGAUCCCUCGCAGCCAGGAAAGGAAACCCACUGA

XM_015093964 CCGGAGAGCUCUGCUCAGGAAGAUCCCUCGCAGCCAGCAAAGGAAACCCUCUGA

XM_019452268 CCGGAGAGCUCUGCUCAGGAAGAUCCCUCGCAGCCAGGAAAGGAGACCCCCUGA

XM_004270956 CCGGAGAGCUCCGCUCGGGAAGAUCCCUCGCAGCCAGGAAAGGAAACCCCCUGA

XM_027541573 CCGGAGAGCUCUGCUCAGGAAGAUCCCUCGCAGCCAGCAAAGGAAACCCUCUGA

XM_027934162 CCGGAGAGCUCUGCUCAGGAAGAUUCCUCACAGCCAGGAAAGGAAGCCCCCUGA

XM_003355541 CCAGAGAGCUCCGCUCAGGAAGAUCACUCGCAGCCAGGAAAGGAAAGCCCCUGA

XM_016119294 CCGGAGAGCUCUGCUCAGGAAGCUCCCUCGCAGCCAGGGAAGGAAACUCCCUGA

XM_006127674 AGUGACCUUCGCUCCCCGGAAGAUCUUUUGCAGUCCGGAAAGGAAAGCCCCUGA
