# Supplementary material for: Unexpected predicted length variation for the coding sequence of the sleep related gene, BHLHE41 in gorilla amidst strong purifying selection across mammals
Source: PLoS One. 2020 Apr 14;15(4):e0223203. doi: 10.1371/journal.pone.0223203 (PMC7156063; doi:10.1371/journal.pone.0223203)
Supplement: S4 Table — (DOCX) [file pone.0223203.s007.docx]

S4 Table. *BHLHE41* mammalian amino acid alignment with reptile outgroup.

NM_030762 ------------------------------------------------------------

XM_520805 ------------------------------------------------------------

XM_019037881 MVRSARELGAGLAGDARALWPNTAWSESKLPAQWGRRECACVCVCVCERGGGGDQLLHTF

XM_002823045 ------------------------------------------------------------

XM_005570417 ------------------------------------------------------------

XM_012093655 ------------------------------------------------------------

XM_011759130 ------------------------------------------------------------

XM_015151321 ------------------------------------------------------------

XM_007967990 ------------------------------------------------------------

XM_025402281 ------------------------------------------------------------

XM_023209042 ------------------------------------------------------------

XM_017507035 ------------------------------------------------------------

XM_019452268 ------------------------------------------------------------

XM_003355541 ------------------------------------------------------------

XM_027934162 ------------------------------------------------------------

XM_012739537 ------------------------------------------------------------

XM_025879601 ------------------------------------------------------------

XM_027593397 ------------------------------------------------------------

XM_016119294 ------------------------------------------------------------

XM_027129408 ------------------------------------------------------------

XM_022577811 ------------------------------------------------------------

XM_019936346 ------------------------------------------------------------

XM_015093964 ------------------------------------------------------------

XM_007446307 ------------------------------------------------------------

XM_024128992 ------------------------------------------------------------

XM_004270956 ------------------------------------------------------------

XM_027541573 ------------------------------------------------------------

XM_006127674 ------------------------------------------------------------

NM_030762 ----------------------------------------------MDEGIPHLQERQLL

XM_520805 ----------------------------------------------MDEGIPHLQERQLL

XM_019037881 NTALKRESERETGDAQIPPRSPKPTVPQIIVQSPKNRNRGNEQQLNMDEGIPHLQERQLL

XM_002823045 ----------------------------------------------MDEGIPHLQERQLL

XM_005570417 ----------------------------------------------MDEGIPHLQERQLL

XM_012093655 ----------------------------------------------MDEGIPHLQERQLL

XM_011759130 ----------------------------------------------MDEGIPHLQERQLL

XM_015151321 ----------------------------------------------MDEGIPHLQERQLL

XM_007967990 ----------------------------------------------MDEGIPHLQERQLL

XM_025402281 ----------------------------------------------MDEGIPHLQERQLL

XM_023209042 ----------------------------------------------MDEGIPHLQERQLL

XM_017507035 ----------------------------------------------MDEGIPHLQERQLL

XM_019452268 ----------------------------------------------MDEGIPHLQERQLL

XM_003355541 ----------------------------------------------MDEGIPHLQERQLL

XM_027934162 ----------------------------------------------MDEGIPHLQERQLL

XM_012739537 ----------------------------------------------MDEGIPHLQERQLL

XM_025879601 ----------------------------------------------MDEGIPHLQERQLL

XM_027593397 ----------------------------------------------MDEGIPHLQERQLL

XM_016119294 ----------------------------------------------MDEGIPHLQERQLL

XM_027129408 ----------------------------------------------MDEGIPHLQERQLL

XM_022577811 ----------------------------------------------MDEGIPHLQERQLL

XM_019936346 ----------------------------------------------MDEGIPHLQERQLL

XM_015093964 ----------------------------------------------MDEGIPHLQERQLL

XM_007446307 ----------------------------------------------MDEGIPHLQERQLL

XM_024128992 ----------------------------------------------MDEGIPHLQERQLL

XM_004270956 ----------------------------------------------MDEGIPHLQERQLL

XM_027541573 ----------------------------------------------MDEGIPHLQERQLL

XM_006127674 ----------------------------------------------MDEGIPRLPDRQLL

NM_030762 EHRDFIGLDYSSLYMCKPKRSMKRDDTKDTYKLPHRLIEKKRRDRINECIAQLKDLLPEH

XM_520805 EHRDFIGLDYSSLYMCKPKRSMKRDDTKDTYKLPHRLIEKKRRDRINECIAQLKDLLPEH

XM_019037881 EHRDFIGLDYSSLYMCKPKRSMKRDDTKDTYKLPHRLIEKKRRDRINECIAQLKDLLPEH

XM_002823045 EHRDFIGLDYSSLYMCKPKRSMKRDDTKDTYKLPHRLIEKKRRDRINECIAQLKDLLPEH

XM_005570417 EHRDFIGLDYSSLYMCKPKRSMKRDDTKDTYKLPHRLIEKKRRDRINECIAQLKDLLPEH

XM_012093655 EHRDFIGLDYSSLYMCKPKRSMKRDDTKDTYKLPHRLIEKKRRDRINECIAQLKDLLPEH

XM_011759130 EHRDFIGLDYSSLYMCKPKRSMKRDDTKDTYKLPHRLIEKKRRDRINECIAQLKDLLPEH

XM_015151321 EHRDFIGLDYSSLYMCKPKRSMKRDDTKDTYKLPHRLIEKKRRDRINECIAQLKDLLPEH

XM_007967990 EHRDFIGLDYSSLYMCKPKRSMKRDDTKDTYKLPHRLIEKKRRDRINECIAQLKDLLPEH

XM_025402281 EHRDFIGLDYSSLYMCKPKRSMKRDDTKDTYKLPHRLIEKKRRDRINECIAQLKDLLPEH

XM_023209042 EHRDFIGLDYSSLYMCKPKRSMKRDDTKDTYKLPHRLIEKKRRDRINECIAQLKDLLPEH

XM_017507035 EHRDFIGLDYSALYMCKPKRSMKRDDTKDTYKLPHRLIEKKRRDRINECIAQLKDLLPEH

XM_019452268 EHRDFIGLDYSSLYMCKPKRSMKRDDSKDTYKLPHRLIEKKRRDRINECIAQLKDLLPEH

XM_003355541 EHRDFIGLDYSSLYMCKPKRSMKRDDSKDTYKLPHRLIEKKRRDRINECIAQLKDLLPEH

XM_027934162 EHRDFIGLDYSSLYMCKPKRSMKRDDSKDTYKLPHRLIEKKRRDRINECIAQLKDLLPEH

XM_012739537 EHRDFIGLDYSSLYMCKPKRSMKRDDSKDTYKLPHRLIEKKRRDRINECIAQLKDLLPEH

XM_025879601 EHRDFIGLDYSSLYMCKPKRSMKRDDSKDTYKLPHRLIEKKRRDRINECIAQLKDLLPEH

XM_027593397 EHRDFIGLDYSSLYMCKPKRSMKRDDSKDTYKLPHRLIEKKRRDRINECIAQLKDLLPEH

XM_016119294 EHRDFIGLDYSSLYMCKPKRSMKRDDSKDTYKLPHRLIEKKRRDRINECIAQLKDLLPEH

XM_027129408 EHRDFIGLDYPSLYMCKPKRSMKRDDSKDTYKLPHRLIEKKRRDRINECIAQLKDLLPEH

XM_022577811 EHRDFIGLDYPSLYMCKPKRSMKRDDSKDTYKLPHRLIEKKRRDRINECIAQLKDLLPEH

XM_019936346 EHRDFIGLDYPSLYMCKPKRSMKRDDSKDTYKLPHRLIEKKRRDRINECIAQLKDLLPEH

XM_015093964 EHRDFIGLDYPSLYMCKPKRSMKRDDSKDTYKLPHRLIEKKRRDRINECIAQLKDLLPEH

XM_007446307 EHRDFIGLDYPSLYMCKPKRSMKRDDSKDTYKLPHRLIEKKRRDRINECIAQLKDLLPEH

XM_024128992 EHRDFIGLDYPSLYMCKPKRSMKRDDSKDTYKLPHRLIEKKRRDRINECIAQLKDLLPEH

XM_004270956 EHRDFIGLDYPSLYMCKPKRSMKRDDSKDTYKLPHRLIEKKRRDRINECIAQLKDLLPEH

XM_027541573 EHRDFIGLDYPSLYMCKPKRSMKRDDSKDTYKLPHRLIEKKRRDRINECIAQLKDLLPEH

XM_006127674 EHVDFIGLDYPSLYLCKPKRGMKRDESKETYKLPHRLIEKKRRDRINECIAQLKDLLPEH

NM_030762 LKLTTLGHLEKAVVLELTLKHLKALTALTEQQHQKIIALQNGERSLKSPIQSDLDAFHSG

XM_520805 LKLTTLGHLEKAVVLELTLKHLKALTALTEQQHQKIIALQNGERSLKSPIQSDLDAFHSG

XM_019037881 LKLTTLGHLEKAVVLELTLKHLKALTALTEQQHQKIIALQNGERSLKSPIQSDLDAFHSG

XM_002823045 LKLTTLGHLEKAVVLELTLKHLKALTALTEQQHQKIIALQNGERSLKSPIQSDLDAFHSG

XM_005570417 LKLTTLGHLEKAVVLELTLKHLKALTALTEQQHQKIIALQNGERSLKSPIQSDLDAFHSG

XM_012093655 LKLTTLGHLEKAVVLELTLKHLKALTALTEQQHQKIIALQNGERSLKSPIQSDLDAFHSG

XM_011759130 LKLTTLGHLEKAVVLELTLKHLKALTALTEQQHQKIIALQNGERSLKSPIQSDLDAFHSG

XM_015151321 LKLTTLGHLEKAVVLELTLKHLKALTALTEQQHQKIIALQNGERSLKSPIQSDLDAFHSG

XM_007967990 LKLTTLGHLEKAVVLELTLKHLKALTALTEQQHQKIIALQNGERSLKSPIQSDLDAFHSG

XM_025402281 LKLTTLGHLEKAVVLELTLKHLKALTALTEQQHQKIIALQNGERSLKSPIQSDLDAFHSG

XM_023209042 LKLTTLGHLEKAVVLELTLKHLKALTALTEQQHQKIIALQNGERSLKSPIQSDLDAFHSG

XM_017507035 LKLTTLGHLEKAVVLELTLKHLKALTALTEQQHQKIIALQNGERSLKSPIQSDLDAFHSG

XM_019452268 LKLTTLGHLEKAVVLELTLKHLKALTALTEQQHQKIIALQNGERSLKSPIQSDLDAFHSG

XM_003355541 LKLTTLGHLEKAVVLELTLKHLKALTALTEQQHQKIIALQNGERSLKSPIQSDLDAFHSG

XM_027934162 LKLTTLGHLEKAVVLELTLKHLKALTALTEQQHQKIIALQNGERSLKSPIQSDLDAFHSG

XM_012739537 LKLTTLGHLEKAVVLELTLKHLKALTALTEQQHQKIIALQNGERSLKSPIQSDLDAFHSG

XM_025879601 LKLTTLGHLEKAVVLELTLKHLKALTALTEQQHQKIIALQNGERSLKSPIQSDLDAFHSG

XM_027593397 LKLTTLGHLEKAVVLELTLKHLKALTALTEQQHQKIIALQNGERSLKSPIQSDLDAFHSG

XM_016119294 LKLTTLGHLEKAVVLELTLKHLKALTALTEQQHQKIIALQNGERSLKSPIQSDLDAFHSG

XM_027129408 LKLTTLGHLEKAVVLELTLKHLKALTALTEQQHQKIIALQNGERSLKSPIQSDLDAFHSG

XM_022577811 LKLTTLGHLEKAVVLELTLKHLKALTALTEQQHQKIIALQNGERSLKSPIQSDLDAFHSG

XM_019936346 LKLTTLGHLEKAVVLELTLKHLKALTALTEQQHQKIIALQNGERSLKSPIQSDLDAFHSG

XM_015093964 LKLTTLGHLEKAVVLELTLKHLKALTALTEQQHQKIIALQNGERSLKSPIQSDLDAFHSG

XM_007446307 LKLTTLGHLEKAVVLELTLKHLKALTALTEQQHQKIIALQNGERSLKSPIQSDLDAFHSG

XM_024128992 LKLTTLGHLEKAVVLELTLKHLKALTALTEQQHQKIIALQNGERSLKSPIQSDLDAFHSG

XM_004270956 LKLTTLGHLEKAVVLELTLKHLKALTALTEQQHQKIIALQNGERSLKSPIQSDLDAFHSG

XM_027541573 LKLTTLGHLEKAVVLELTLKHLKALTALTEQQHQKIIALQNGERSLKSPIQSDLDAFHSG

XM_006127674 LKLTTLGHLEKAVVLELTLKHLKALTALTEQQHQNIIALQNGERAMKSPIQCDLDAFHSG

NM_030762 FQTCAKEVLQYLSRFESWTPREPRCVQLINHLHAVATQFLPTPQLLTQQVPLSKGTGAPS

XM_520805 FQTCAKEVLQYLSRFESWTPREPRCVQLINHLHAVATQFLPTPQLLTQQVPLSKGTGAPS

XM_019037881 FQTCAKEVLQYLSRFESWTPREPRCVQLINHLHAVATQFLPTPQLLTQQVPLSKGTGAPS

XM_002823045 FQTCAKEVLQYLSRFESWTPREPRCVQLINHLHAVATQFLPTPQLLTQQVPLSKGTGAPS

XM_005570417 FQTCAKEVLQYLSRFESWTPREPRCVQLINHLHAVATQFLPTPQLLTQQVPLSKGTGAPS

XM_012093655 FQTCAKEVLQYLSRFESWTPREPRCVQLINHLHAVATQFLPTPQLLTQQVPLSKGTGAPS

XM_011759130 FQTCAKEVLQYLSRFESWTPREPRCVQLINHLHAVATQFLPTPQLLTQQVPLSKGTGAPS

XM_015151321 FQTCAKEVLQYLSRFESWTPREPRCVQLINHLHAVATQFLPTPQLLTQQVPLSKGTGAPS

XM_007967990 FQTCAKEVLQYLSRFESWTPREPRCVQLINHLHAVATQFLPTPQLLTQQVPLSKGTGAPS

XM_025402281 FQTCAKEVLQYLSRFESWTPREPRCVQLINHLHAVATQFLPTPQLLTQQVPLSKGTGAPS

XM_023209042 FQTCAKEVLQYLSRFESWTPREPRCVQLINHLHAVATQLLPTPQLLTQQVPLSKGTGAPS

XM_017507035 FQTCAKEVLQYLSRFESWTPREPRCVQLINHLHAVATQFLPTPQLLTQQVPLSKGTGAPS

XM_019452268 FQTCAKEVLQYLSRFESWTPREQRCVQLINHLHAVATQFLPTPQLLTQQVPLSKGTGAPS

XM_003355541 FQTCAKEVLQYLARFESWTPREPRCVQLINHLHAVATQFLPTPQLLTQQVPLSKGTGAPT

XM_027934162 FQTCAKEVLQYLSRFESWTPREPRCVQLINHLHAVATQFLPTPQLLTQQVPLSKGTGAPS

XM_012739537 FQTCAKEVLQYLSRFESWTPREPRCVQLINHLHAVATQFLPTPQLLTQQVPLSKGAGAAS

XM_025879601 FQTCAKEVLQYLSRFESWTPREQRCVQLINHLHAVATQFLPTPQLLTQQVPLSKGTGAPS

XM_027593397 FQTCAKEVLQYLSRFESWTPREQRCVQLINHLHAVATQFLPTPQLLTQQVPLSKGTGAPS

XM_016119294 FQTCAKEVLQYLSRFESWTPREPRCVQLINHLHAVATQFLPTPQLLTQQVPLSKGTGAPS

XM_027129408 FQTCAKEVLQYLARFESWTPREPRCVQLINHLHAVATQFLPTPQLLTQQVPLSKGTGVPS

XM_022577811 FQTCAKEVLQYLARFESWTPREPRCVQLINHLHAVATQFLPTPQLLTQQVPLSKGTGVPS

XM_019936346 FQTCAKEVLQYLARFESWTPREPRCVQLINHLHAVATQFLPTPQLLTQQVPLSKGTGVPS

XM_015093964 FQTCAKEVLQYLARFESWTPREPRCVQLINHLHAVATQFLPTPQLLTQQVPLSKGTGAPT

XM_007446307 FQTCAKEVLQYLARFESWTPREPRCVQLINHLHAVATQFLPTPQLLTQQVPLSKGTGVPS

XM_024128992 FQTCAKEVLQYLARFESWTPREPRCVQLINHLHAVATQFLPTPQLLTQQVPLSKGTGVPS

XM_004270956 FQTCAKEVLQYLARFESWTPREPRCVQLINHLHAVATQFLPTPQLLTQQVPLSKGTGVPS

XM_027541573 FQTCAKEVLQYLARFESWTPREPRCVQLINHLHAVATQFLPTPQLLTQQVPLSKGTAAPT

XM_006127674 FQTCAKEVLQYLSRFESWTPREQRCAQLVNHLHAVSTQFLPSPQLLTPQVPASKGS----

NM_030762 A---AGSAAAPCLERAGQKLEPLAYCVPVIQRTQPSAEL-AAENDTDTDSGYGGEAEARP

XM_520805 A---AGSAAAPCLERAGQKLEPLAYCVPVIQRTQPSAEL-AAENDTDTDSGYGGEAEARP

XM_019037881 A---AGSAAAPCLERAGQKLEPLAYCVPVIQRTQPSAEL-AAENDTDTDSGYGGEAEARP

XM_002823045 A---AGSAAAPCLERAGQKLEPLAYCVPVIQRTQPSAEL-AAENDTDTDSGYGGEAEARP

XM_005570417 A---AGSAAAPCLERAGQKLEPLAYCVPVIQRTQPSAEL-AAENDTDTDSGYGGEAEARP

XM_012093655 A---AGSAAAPCLERAGQKLEPLAYCVPVIQRTQPSAEL-AAENDTDTDSGYGGEAEARP

XM_011759130 A---AGSAAAPCLERAGQKLEPLAYCVPVIQRTQPSAEL-AAENDTDTDSGYGGEAEARP

XM_015151321 A---AGSAAAPCLERAGQKLEPLAYCVPVIQRTQPSAEL-AAENDTDTDSGYGGEAEARP

XM_007967990 A---AGSAAAPCLERAGQKLEPLAYCVPVIQRTQPSAEL-AAENDTDTDSGYGGEAEARP

XM_025402281 A---AGSAAAPCLERAGQKLEPLAYCVPVIQRTQPSAEL-AAENDTDTDSGYGGEAEARP

XM_023209042 A---AGSAAAPCLERTGQKLEPLAYCVPVIQRTQPNAEL-AAENDTDTDSGYGGEAEARP

XM_017507035 A---AGSATAPCLERAAQKLEPLAHCVPVIQRTQPSAEL-AAENDTDTDSGYGGEAEARP

XM_019452268 AAAPAGSAAAPCLERAGQKLEPLAHCVPVIQRTQPSAEL-AAENDTDTDSGYGGEAEARP

XM_003355541 TA-PAGSVAAACLERAGQKLEPLAHCVPVIQRTQPSAEL-AAENDTDTDSGYGGEAEARP

XM_027934162 APAPTGSTAAPCLERAGQKLEPLAHCVPVIQRTQPSAEL-AAENDTDTDSGYGGEAEARP

XM_012739537 AAAPAGSAAAPCLERAGQKLEPLAHCVPVIQRTQPSAEL-AAENDTDTDSGYGGEAEARP

XM_025879601 AAAPTGSAAAPCLERAGQKLEPLAHCVPVIQRTQPSAEL-AAENDTDTDSGYGGEAEARP

XM_027593397 AAAPTGSAAAPCLERAGQKLEPLAHCVPVIQRTQPSAEL-AAENDTDTDSGYGGEAEARP

XM_016119294 ASAPAGSAAAPCLERAGQKLEPLAHCVPVIQRTQPSAEL-AAENDTDTDSGYGGEAEARP

XM_027129408 APTPAGSGAAPCLERAGQKLEPLAHCVPVIQRTQPSAEL-AAENDTDTDSGYGGEAEARP

XM_022577811 AATPAGSGAAPCLERAGQKLEPLAHCVPVIQRTQPSAEL-AAENDTDTDSGYGGEAEARP

XM_019936346 AATPAGSGAAPCLERAGQKLEPLAHCVPVIQRTQPSAEL-AAENDTDTDSGYGGEAEARP

XM_015093964 AAAPAGSGAAPCLERAGQKLEPLAHCVPVIQRTQPSAELAAAENDTDTDSGYGGEAEARP

XM_007446307 AATPAGSGAAPCLERAGQKLEPLAHCVPVIQRTQPSAEL-AAENDTDTDSGYGGEAEARP

XM_024128992 AAAPAGSGAAPCLERAGQKLEPLAHCVPVIQRTQPSAEL-AAENDTDTDSGYGGEAEARP

XM_004270956 AATPAGSGAAPCLERAGQKLEPLAHCVPVIQRTQPSAEL-AAENDTDTDSGYGGEAEARP

XM_027541573 AAAPAGSGAAPCLERAGQKLEPLAHCVPVIQRTQPSSELAAAENDTDTDSGYGGEAEARP

XM_006127674 ------SSSSCAQDRTGQKLEAQTNCVPVIQRTHPPAEL-SGENDTDTDSGYGGESEGRP

NM_030762 DREKGKGAGASRVTIKQEPPGEDSPAPKRMKLDSRGGGSGGGPGGGAAAAAAALLGPDPA

XM_520805 DREKGKGAGASRVTIKQEPPGEDSPAPKRMKLDSRGGGSGGGPGGGAAAAAAALLGPDPA

XM_019037881 DREKGKGAGASRVTIKQEPPGEDSPAPKRMKLDSR----GGGPGGGAAAAAAALLGPDPA

XM_002823045 DREKGKGAGASRVTIKQEPPGEDSPAPKRMKLDSRGGGSGGGPGGGAAAAAAALLGPDPA

XM_005570417 DREKGKGAGASRVTIKQEPPGEDLPAPKRMKLDSRGGGSGGGPGGGAAAAAAALLGPDPA

XM_012093655 DREKGKGAGASRVTIKQEPPGEDLPAPKRMKLDSRGGGSGGGPGGGAAAAAAALLGPDPA

XM_011759130 DREKGKGAGASRVTIKQEPPGEDLPAPKRMKLDSRGGGSGGGPGGGAAAAAAALLGPDPA

XM_015151321 DREKGKGAGASRVTIKQEPPGEDLPAPKRMKLDSRGGGSGGGPGGGAAAAAAALLGPDPA

XM_007967990 DREKGKGAGASRVTIKQEPPGEDLPAPKRMKLDTRGGGSGGGPGGGAAAAAAALLGPDPA

XM_025402281 DREKGKGAGASRVTIKQEPPGEDLPAPKRMKLDSR----GGGPGGGAAAAAAALLGPDPA

XM_023209042 DREKGKGAGASRVTIKQEPPGEDLPAPKRMKLDSR----GGGPGGGAAAAAAALLGPDPA

XM_017507035 DREKGKGAGASRVTIKQEPPGEDSPAPKRMKLDSRGG--GGGPGGGAAAAAAALLGPDPA

XM_019452268 DREKGKGAGASRVTIKQEPPGEDSPAPKRMKLDSRG---GGGLGGGAAAAAAALLGPDPA

XM_003355541 DREKGKGAGASRVTIKQEPPGEDSPAPKRMKLDSRGG--GGGPGGGAAAAAAALLGPDPA

XM_027934162 DREKSKGAGASRVTIKQEPPGEDSPAPKRMKLDSRG---GGGPGGGAAAAAAALLGPDPA

XM_012739537 DRGKSKGAGASRVTIKQEPPGEDSPAPKRMRLDSRGGAAGGGGPGGAARAAAALLGPDPA

XM_025879601 DREKGKGSGTGRVTIKQEPPGEDSPAPKRMKLDSRGG--GGGLGGGAAAAAAALLGPDPA

XM_027593397 DREKGKGSGTGRVTIKQEPPGEDSPAPKRMKLDSRGG--GGGLGGGAAAAAAALLGPDPA

XM_016119294 DREKGKSAGASRVTIKQEPPGEDSPAPKRMRLDSR-----GGAGGGAAAAAAALLGPDPA

XM_027129408 DREKGKGAGASRVTIKQEPPGEDSPAPKRMKLDSRS---GGGLGGGAAAAAAALLGPDPA

XM_022577811 DREKGKGAGTSRVTIKQEPPGEDSPAPKRMKLDSRS---GGGLGGGAAAAAAALLGPDPA

XM_019936346 DREKGKGAGASRVTIKQEPPGEDSPAPKRMKLDSRS---GGGLGGGAAAAAAALLGPDPA

XM_015093964 DREKGKGAGTSRVTIKQEPPGEDLPAPKRMRLDTRGGG-GGGPGGGAAAAAAALLGPDPA

XM_007446307 DREKGKGAGASRVTIKQEPPGDDSPAPKRMKLDSRSG--GGGLGGGAAAAAAALLGPDPA

XM_024128992 DREKGKGAGASRVTIKQEPPGEDSPAPKRMKLDSRS---GGGLGGGAAAAAAALLGPDPA

XM_004270956 DREKGKGAGASRVTIKQEPPGEDSPAPKRMKLDSRS---GGGLGGGAAAAAAALLGPDPA

XM_027541573 DREKGKGAGASRVTIKQEPPGEDSPAPKRMRLDTRGCG-GGGPGGGAAAAAAALLGPDPT

XM_006127674 DREKGQAARLPSLTIKQEPAGDEAPAPKRLKLDCTSS-SSSSNSHSAPLPSAAALSPDPA

NM_030762 AAAALLRPDAALLSSLVAFGGGG-GAPFPQPAA---AAAPFCLPFCFLSPSAAAAYVQPF

XM_520805 AAAALLRPDAALLSSLVAFGGGG-GAPFPQPAA---AAAPFCLPFCFLSPSAAAAYVQPF

XM_019037881 AAAALLRPDAALLSSLVAFGGGG-GAPFPQPA----------------------------

XM_002823045 AAAALLRPDAALLSSLVAFGGGG-GAPFPQPAA---AAAPFCLPFCFLSPSAAAAYVQPF

XM_005570417 AAAALLRPDAALLSSLVAFGGGG-GAPFPQPAA---AAAPFCLPFCFLSPSAAAAYVQPF

XM_012093655 AAAALLRPDAALLSSLVAFGGGG-GAPFPQPAA---AAAPFCLPFCFLSPSAAAAYVQPF

XM_011759130 AAAALLRPDAALLSSLVAFGGGG-GAPFPQPAA---AAAPFCLPFCFLSPSAAAAYVQPF

XM_015151321 AAAALLRPDAALLSSLVAFGGGG-GAPFPQPAA---AAAPFCLPFCFLSPSAAAAYVQPF

XM_007967990 AAAALLRPDAALLSSLVAFGGGG-GAPFPQPAA---AAAPFCLPFCFLSPSAAAAYVQPF

XM_025402281 AAAALLRPDAALLSSLVAFGGGG-GAPFPQPAA---AAAPFCLPFCFLSPSAAAAYVQPF

XM_023209042 AAAALLRPDAALLSSLVAFGGGG-GAPFPQPAA---AAAPFCLPFCFLSPSAAAAYVQPF

XM_017507035 AAAALLRPDAALLSSLVAFGGGG-GAPFPQPAA---AAAPFCLPFYFLSPSAAAAYVQPF

XM_019452268 AAAALLRPDAALLSSLVAFGGGG-GAPFAQPAAAAAAAAPFCLPFYFLSPSAAAAYVQPF

XM_003355541 AAAALLRPDAALLSSLVAFGGGG-GAPFAQPAA---AAAPFCLPFYFLSPSAAAAYVQPF

XM_027934162 AAAALLRPDAALLSSLVAFGGGG-GAPFAQPA----AAAPFCLPFYFLSPSAAAAYVQPF

XM_012739537 AAAALLRPDAALLSSLVAFGGGG-GAPFAQPAA---AAAPFCLPFYFLSPSAAAAYVQPF

XM_025879601 AAAALLRPDAALLSSLVAFGGGG-GAPFAQPAAAAAAAAPFCLPFYFLSPSAAAAYVQPF

XM_027593397 AAAALLRPDAALLSSLVAFGGGG-GAPFAQPAAAAAAAAPFCLPFYFLSPSAAAAYVQPF

XM_016119294 AAAALLRPDAALLSSLVAFGGGG-GAPFAQPAA---AAAPFCLPFYFLSPSAAAAYVQPF

XM_027129408 APAALLRPDAALLSSLVAFGGGG-GAPFAQPAA---AAAPFCLPFYFLSPSAAAAYVQPF

XM_022577811 APAALLRPDAALLSSLVAFGGGG-GAPFAQPAA---AAAPFCLPFYFLSPSAAAAYVQPF

XM_019936346 APAALLRPDAALLSSLVAFGGGG-GAPFAQPAA---AAAPFCLPFYFLSPSAAAAYVQPF

XM_015093964 AAAALLRPDAALLSSLVAFGGGG-GAPFAQPAA---AAAPFCLPFYFLSPSAAAAYVQPF

XM_007446307 TPAALLRPDAALLSSLVAFGGGG-GAPFAQPAA---AAAPFCLPFYFLSPSAAAAYVQPF

XM_024128992 APAALLRPDAALLSSLVAFGGGG-GAPFAQPAA---AAAPFCLPFYFLSPSAAAAYVQPF

XM_004270956 APAALLRPDAALLSSLVAFGGGG-GAPFAQPAA---AAAPFCLPFYFFSPSAAAAYVQPF

XM_027541573 AAAALLRPDAALLSSLVAFGGGG-GAPFAQPAA---AAAPFCLPFYFLSPSAAAAYVQPF

XM_006127674 A-AALLRPDAALLSSLLAFGGGGAGAPFGQQA-----AAPLCLPFYFLSPSAAAAYMQPL

NM_030762 LDKSGLEKYLYPAAAAAPFPLLYPGIPAPAAAAAAAAAAAAAAAAFPCLSSVLSPPPEK-

XM_520805 LDKSGLEKYLYPAAAAAPFPLLYPGIPAPAAAAAAAAAAAAAAAAFPCLSSVLSPPPEK-

XM_019037881 -------------------------------------AAAAAAAAFPCLSSVLSPPPEK-

XM_002823045 LDKSGLEKYLYPAAAAAPFPLLYPGIPAPAAAAAAAAAAAAAAAAFPCLSSVLSPPPEK-

XM_005570417 LDKSGLEKYLYPAAAAAPFPLLYPGIPAPAAAAAAAAAAAAAAAAFPCLSSVLSPPPEK-

XM_012093655 LDKSGLEKYLYPAAAAAPFPLLYPGIPAPAAAAAAAAAAAAAAAAFPCLSSVLSPPPEK-

XM_011759130 LDKSGLEKYLYPAAAAAPFPLLYPGIPAPAAAAAAAAAAAAAAAAFPCLSSVLSPPPEK-

XM_015151321 LDKSGLEKYLYPAAAAAPFPLLYPGIPAPAAAAAAAAAAAAAAAAFPCLSSVLSPPPEK-

XM_007967990 LDKSSLEKYLYPAAAAAPFPLLYPGIPAPAAAAAAAAAAAAAAAAFPCLSSVLSPPPEK-

XM_025402281 LDKSGLEKYLYPAAAAAPFPLLYPGIPAPAAAAAAAAAAAAAAAAFPCLSSVLSPPPEK-

XM_023209042 LDKSGLEKYLYPAAAATPFPLLYPGIPAPAAAAAAAAAAAAAAAAFPCLSSVLSPPPEK-

XM_017507035 LDKSGLEKYLYPAAAAAPFPLLYPGIPAPAA-AAAAAAAAAAAAAFPCLSSVLSPPPEK-

XM_019452268 LDKSGLEKYLYPAAAAAPFPLLYPGIPAPAA-AAAAAAAAAAAAAFPCLSSVLSPPPEK-

XM_003355541 LDKSGLEKYLYPAAAAAPFPLLYPGIPAPAA-AAAAAAAAAAAAAFPCLSSVLSPPPEK-

XM_027934162 LDKSGLEKYLYPTAAAAPFPLLYPGIPAPAA-AAAAAAAAAAAAAFPCLSSVLSPPPEK-

XM_012739537 LDKSGLEKYLYPAAAAAPFPLLYPGIPAPAA-AAAAAAAAAAAAAFPCLSSVLSPPPEK-

XM_025879601 LDKSGLEKYLYPAAAAAPFPLLYPGIPAPAA-AAAAAAAAAAAAAFPCLSSVLSPPPEK-

XM_027593397 LDKSGLEKYLYPAAAAAPFPLLYPGIPAPAA-AAAAAAAAAAAAAFPCLSSVLSPPPEK-

XM_016119294 LDKSSLEKYLYPAAAAAPFPLLYPGIPAPAA-AAAAAAAAAAAAAFPCLSSVLSPPPEK-

XM_027129408 LDKSGLEKYLYPAAAAAPFPLLYPGIPAPAA-AAAAAAAAAAAAAFPCLSSVLSPPPEK-

XM_022577811 LDKSGLEKYLYPAAAAAPFPLLYPGIPAPAAAAAAAAAAAAAAAAFPCLSSVLSPPPEK-

XM_019936346 LDKSGLEKYLYPAAAAAPFPLLYPGIPAPAA-AAAAAAAAAAAAAFPCLSSVLSPPPEK-

XM_015093964 LDKSGLEKYLYPAAAAAPFPLLYPGIPAPAA-AAAAAAAAAAAAAFPCLSSVLSPPPEKA

XM_007446307 LDKSGLEKYLYPAAAAAPFPLLYPGIPAPAA-AAAAAAAAAAAAAFPCLSSVLSPPPEK-

XM_024128992 LDKSGLEKYLYPAAAAAPFPLLYPGIPAPAA-AAAAAAAAAAAAAFPCLSSVLSPPPEKA

XM_004270956 LDKSGLEKYLYPAAAAAPFPLLYPGIPAPA--AAAAAAAAAAAAAFPCLSSVLSPPPEK-

XM_027541573 LDKSGLEKYLYPAAAAAPFPLLYPGIPAPAA-AAAAAAAAAAAAAFPCLSSVLSPPPEKA

XM_006127674 LDKSNLEKYLYPAAA--PIPLLYPGIP------------AQAAAAFPCLSSVLA-SAEKA

NM_030762 -AGAAAATLLPHEVAPLGAPHPQHPHGRTHLPFAGPREPGNPESSAQEDPSQPGKEAP

XM_520805 -AGAAAATLLPHEVAPLGAPHPQHPHGRTHLPFAGPREPGNPESSAQEDPSQPGKEAP

XM_019037881 -AGAAAATLLPHEVAPLGAPHPQHPHGRTHLPFAGPREPGNPESSAQEDPSQPGKEAP

XM_002823045 -AGAAAATLLPHEVAPLGAPHPQHPHGRTHLPFAGPREPGNSESSAQEDPSQPGKEAP

XM_005570417 -AGAAAATLLPHEVAPLGAPHPQHPHGRTHLPFAGPREPGNPESSAQEDPSQPGKEAP

XM_012093655 -AGAAAATLLPHEVAPLGAPHPQHPHGRTHLPFAGPREPGNPESSAQEDPSQPGKEAP

XM_011759130 -AGAAAATLLPHEVAPLGAPHPQHPHGRTHLPFAGPREPGNPESSAQEDPSQPGKEAP

XM_015151321 -AGAAAATLLPHEVAPLGAPHPQHPHGRTHLPFAGPRESGNPESSAQEDPSQPGKEAP

XM_007967990 -AGAAAATLLPHEVAPLGAPHPQHPHGRTHLPFAGPREPGNPESSAQEDPSQPGKEAP

XM_025402281 -AGAAAATLLPHEVAPLGAPHPQHPHGRTHLPFAGPREPGNPESSAQEDPSQPGKEAP

XM_023209042 -AGAAAATLLPHEVAPLGAPHPQHPHGRTHLPFAGPREPGNPESSAQEDPSQPGKEAP

XM_017507035 -AGAAAATLLPHEVAPPGAPHPPHPHGRTHLPFAGPREPGNPESSAQEDPSQPGKEAP

XM_019452268 -AGAAAATLLPHEVAPPGALHPPHPHGRTHLSFAGAREPGNPESSAQEDPSQPGKETP

XM_003355541 AAAAAAATLLPHEVASPGALHPPHPHGRTHLPFAGPREPGNPESSAQEDHSQPGKESP

XM_027934162 AGAAAAATLLSHEVAPPGALHPPHPHGRTHLPFAGPREPGNPESSAQEDSSQPGKEAP

XM_012739537 AAGAAAATLLPHEVAPPAALHAPHPHGRTHLPFAGPREPGNPESSAQEDPSQPGKEGP

XM_025879601 -ASAAAATLLPHEVAPPGALHPPHPHGRTHLSFAGAREPGNPESSAQEDPSQPGKETH

XM_027593397 -ASAAAATLLPHEVAPPGALHPPHPHGRTHLSFAGAREPGNPESSAQEDPSQPGKETH

XM_016119294 VGAAAAATLLPHEVAPPGSLHPPHPHGRTHLPFAGPREHGNPESSAQEAPSQPGKETP

XM_027129408 AAAAAAATLLPHEVAPPGALHPPPPHGRTHLPFAGRSEPGNPESSAREDPSQPGKETP

XM_022577811 AAAAAAATLLPHEVAPPGALHPPPPHGRTHLPFAGRSEPGNPESSSQEDPSQPGKETP

XM_019936346 AAAAAAATLLPHEVAPPGALHPPPPHGRTHLPFAGRSEPGNPESSAREDPSQPGKETP

XM_015093964 AAAAAAATLLPHEVAPPGALHPAHPHGRTHLPFAGAREPGNPESSAQEDPSQPAKETL

XM_007446307 AAAAAAATLLPHEVAPPGALHPAPPHGRTHLPFAVRSEPGNPESSAQEDPSQPGKETP

XM_024128992 AAAAAAATLLPHEVAPPGALRPPPPHGRTHLPFAGRSEPGNPESSAQEDPSQPGKETP

XM_004270956 AAAAAAATLLPHEVAPPGALHPPPPHGRTHLPFAGRSEPGNPESSAREDPSQPGKETP

XM_027541573 AAAAAAATLLPHEVAPPGALHPAHPHGRTHLPFAGAREPGNPESSAQEDPSQPAKETL

XM_006127674 NAAAAASALLPLDVVSP-SPHLPHPFAAA---CETGTSVGDSDLRSPEDLLQSGKESP
